# Supplementary material for: A methylation clock model of mild SARS‐CoV‐2 infection provides insight into immune dysregulation
Source: Mol Syst Biol. 2023 Mar 15;19(5):e11361. doi: 10.15252/msb.202211361 (PMC10167476; doi:10.15252/msb.202211361)
Supplement: Supplementary file 1 — Appendix [file MSB-19-e11361-s003.pdf]

Appendix to:

A methylation clock model of mild SARS-CoV-2 infection provides insight into immune dysregulation

Weiguang Mao, Clare M. Miller, Venugopalan D. Nair, Yongchao Ge, Mary Anne S. Amper, Antonio Cappuccio, Mary-Catherine George, Carl W. Goforth, Kristy Guevara, Nada Marjanovic, German Nudelman, Hanna Pincas, Irene Ramos, Rachel S. G. Sealfon, Alessandra Soares-Schanoski, Sindhu Vangeti, Mital Vasoya, Dawn L. Weir, Elena Zaslavsky, *Biobank team*, Seunghee Kim-Schulze, Sacha Gnjatic, Miriam Merad, Andrew G. Letizia, Olga G. Troyanskaya, Stuart C. Sealfon, Maria Chikina

Table of contents

1. **Appendix Figures S1-9** ………………………………………………………… p.2-17
   - **Appendix Fig. S1** …………………………………………………...…....... p.3-4
   - **Appendix Fig. S2**……………………………………………………..……..... p.5-6
   - **Appendix Fig. S3** ……………………………………………………………..… p.7-8
   - **Appendix Fig. S4** …………………………………………………………...… p.9-10
   - **Appendix Fig. S5** ………………………………………….…………………. p.11
   - **Appendix Fig. S6** ………………………………………..………......... p.12-13
   - **Appendix Fig. S7** ……………………….……………………………………. p.14-15
   - **Appendix Fig. S8** ……………………………………...……………….... p.16-17
   - **Appendix Fig. S9** ……………………………………………………..………. p.17

2. **References** ………………………………………………………………...... p.18

1. Appendix Figures S1-9

a

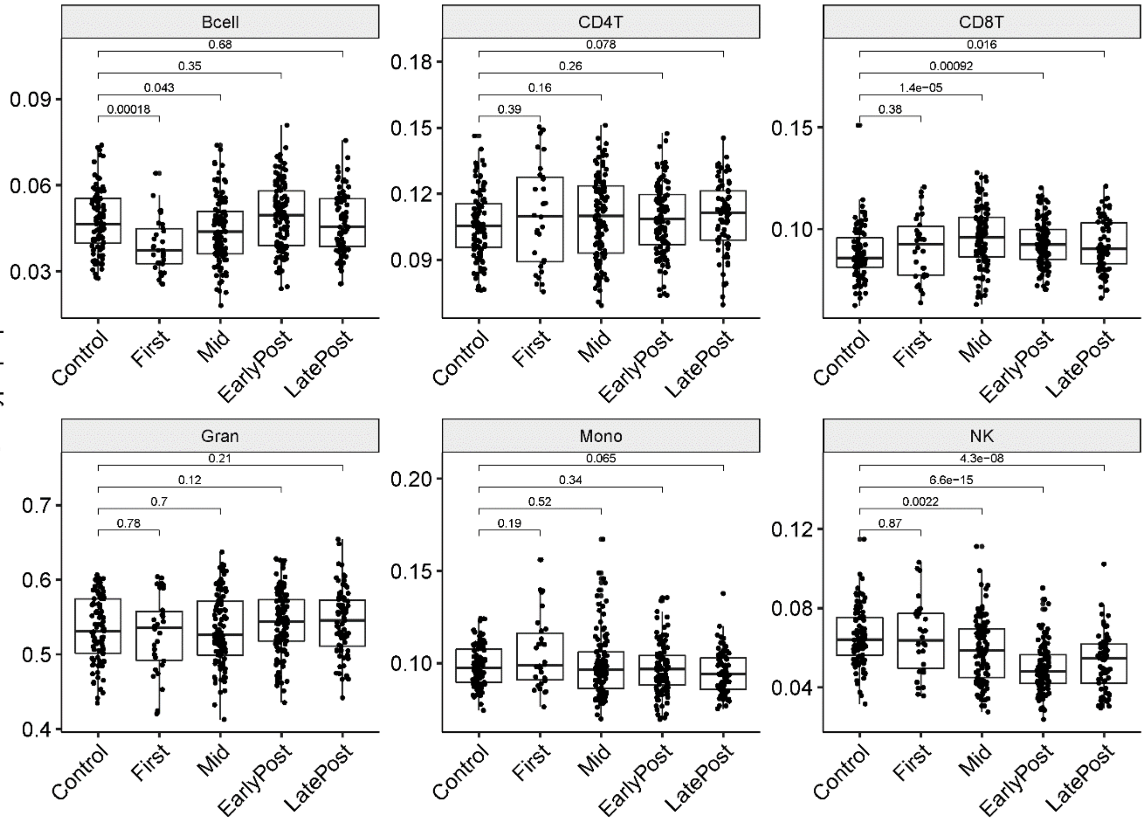
Cell type proportions
Bcell
0.68
0.35
0.043
0.00018
CD4T
0.078
0.26
0.16
0.39
CD8T
0.016
0.00092
1.4e-05
0.38
Gran
0.21
0.12
0.7
0.78
Mono
0.065
0.34
0.52
0.19
NK
4.3e-08
6.6e-15
0.0022
0.87
Control
First
Mid
EarlyPost
LatePost

b

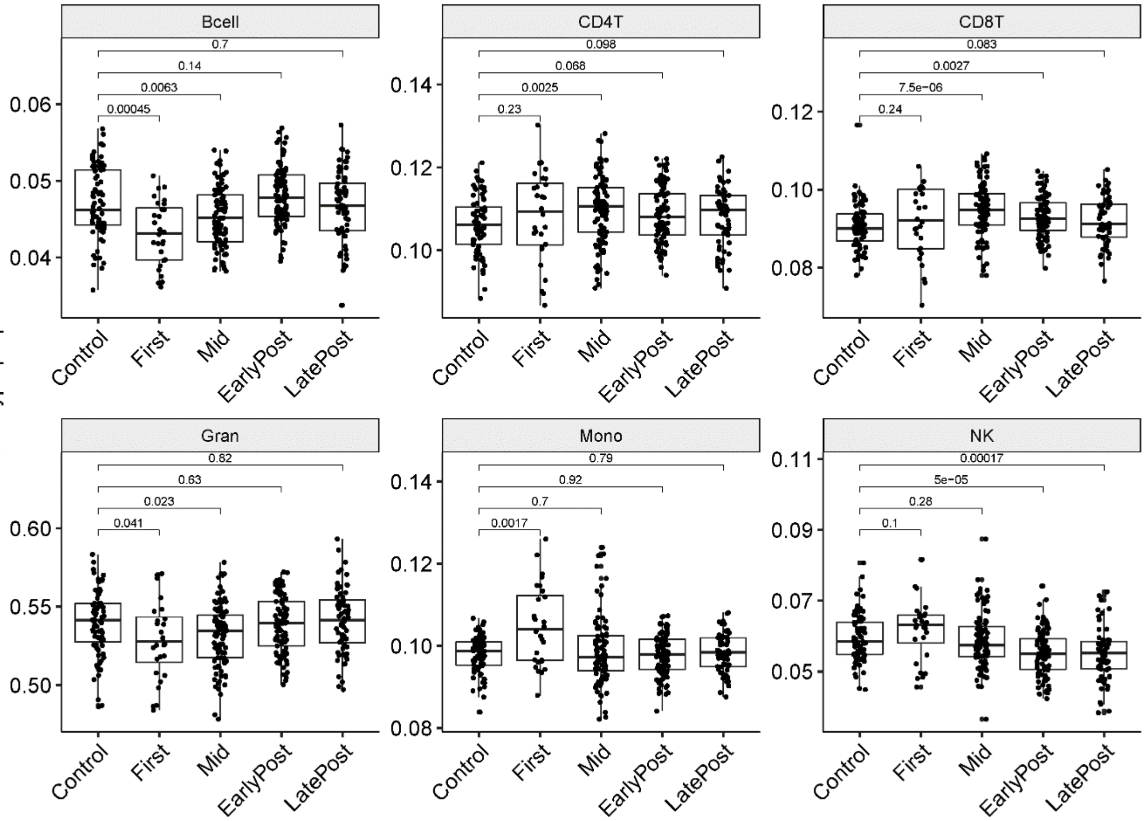
Cell type proportions
Bcell
0.7
0.14
0.0063
0.00045
CD4T
0.098
0.068
0.0025
0.23
CD8T
0.083
0.0027
7.5e-06
0.24
Gran
0.82
0.63
0.023
0.041
Mono
0.79
0.92
0.7
0.0017
NK
0.00017
5e-05
0.28
0.1
Control
First
Mid
EarlyPost
LatePost

Appendix Figure S1: Cell type proportion variation across periods relative to first PCR positive test. a, Boxplot of inferred cell type proportions based on all methylation samples. Cell type proportions at each period after infection were compared with pre-infection cell type proportions using two-tailed Wilcoxon rank sum test. b, Boxplot of inferred cell type proportions based on all RNA-seq samples. Cell type proportions at each period after infection were compared with pre-infection cell type proportions using two-tailed Wilcoxon rank sum test. See Methods for further information on this analysis.

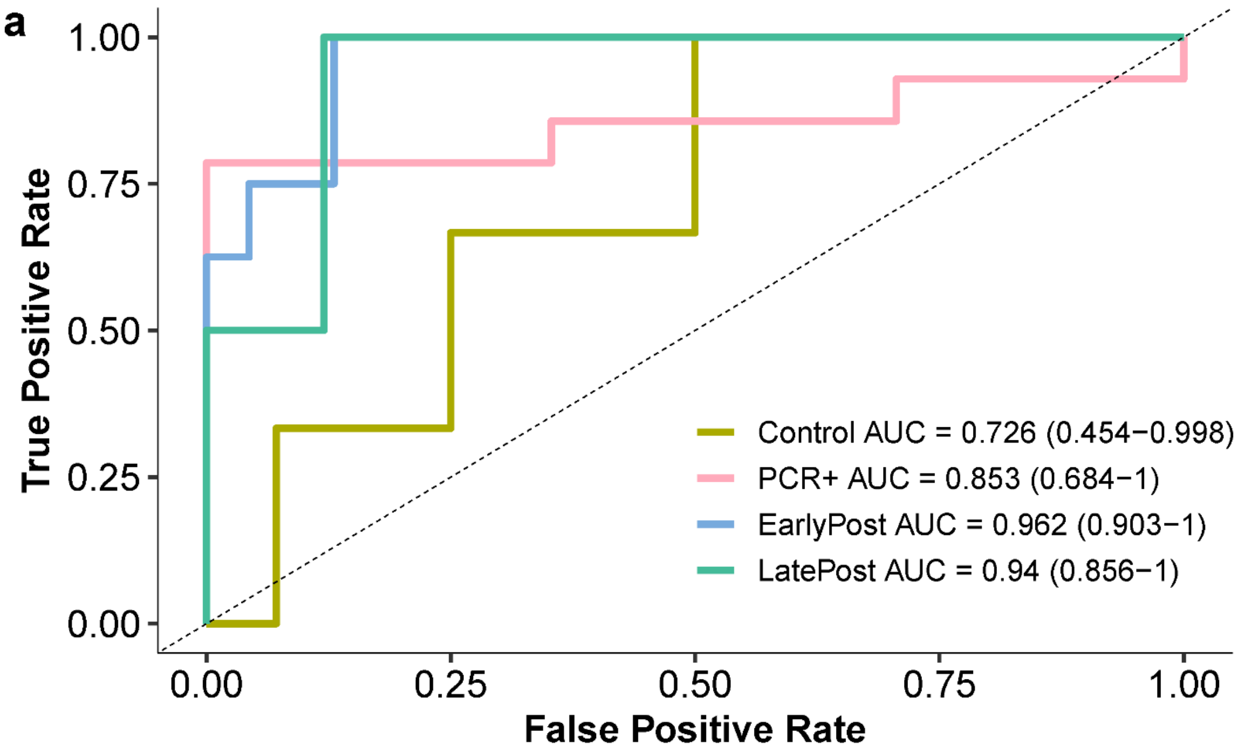
a
1.00
0.75
0.50
0.25
0.00
True Positive Rate
0.00
0.25
0.50
0.75
1.00
False Positive Rate
Control AUC = 0.726 (0.454−0.998)
PCR+ AUC = 0.853 (0.684−1)
EarlyPost AUC = 0.962 (0.903−1)
LatePost AUC = 0.94 (0.856−1)

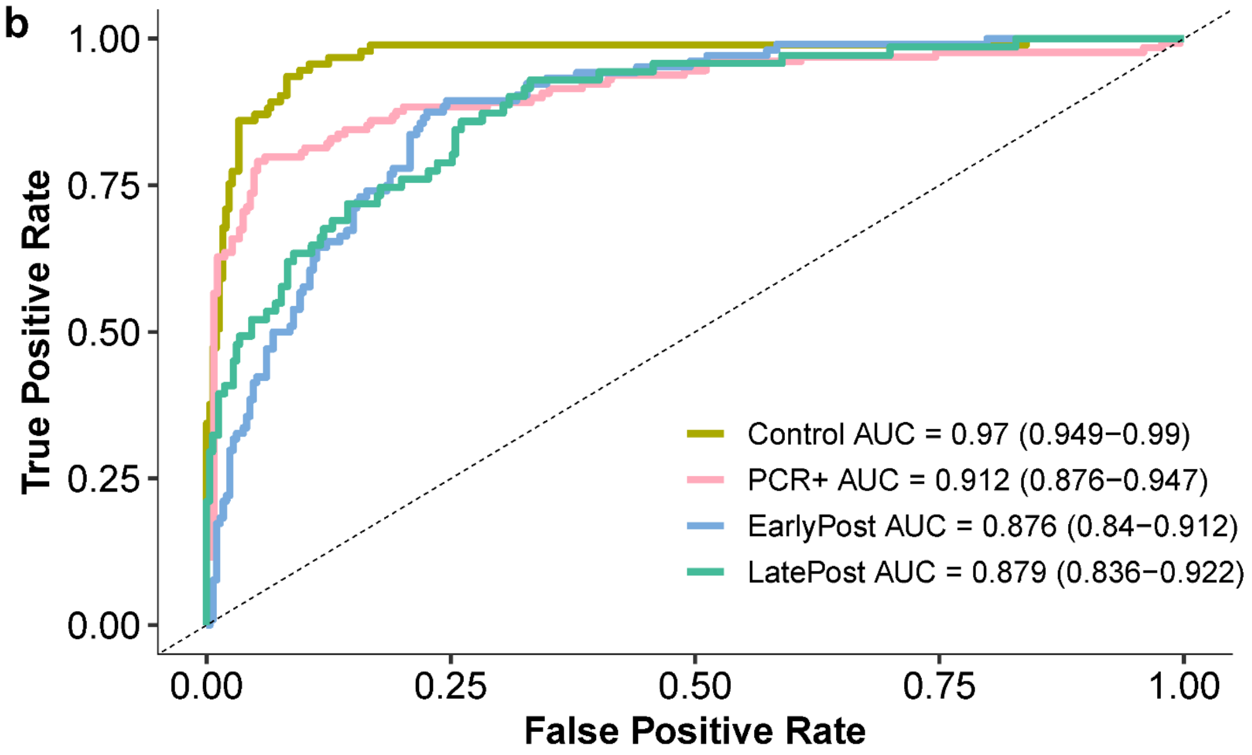
b
1.00
0.75
0.50
0.25
0.00
True Positive Rate
0.00
0.25
0.50
0.75
1.00
False Positive Rate
Control AUC = 0.97 (0.949−0.99)
PCR+ AUC = 0.912 (0.876−0.947)
EarlyPost AUC = 0.876 (0.84−0.912)
LatePost AUC = 0.879 (0.836−0.922)

Appendix Figure S2: Comparison of multiclass classifier performance on samples from male and female participants. a, Receiver operator curve obtained from multiclass classifier applied to samples from female participants. The 95% confidence intervals are indicated in the key. **b**, Receiver operator curve obtained from multiclass classifier applied to samples from male participants. The 95% confidence intervals are indicated in the key.

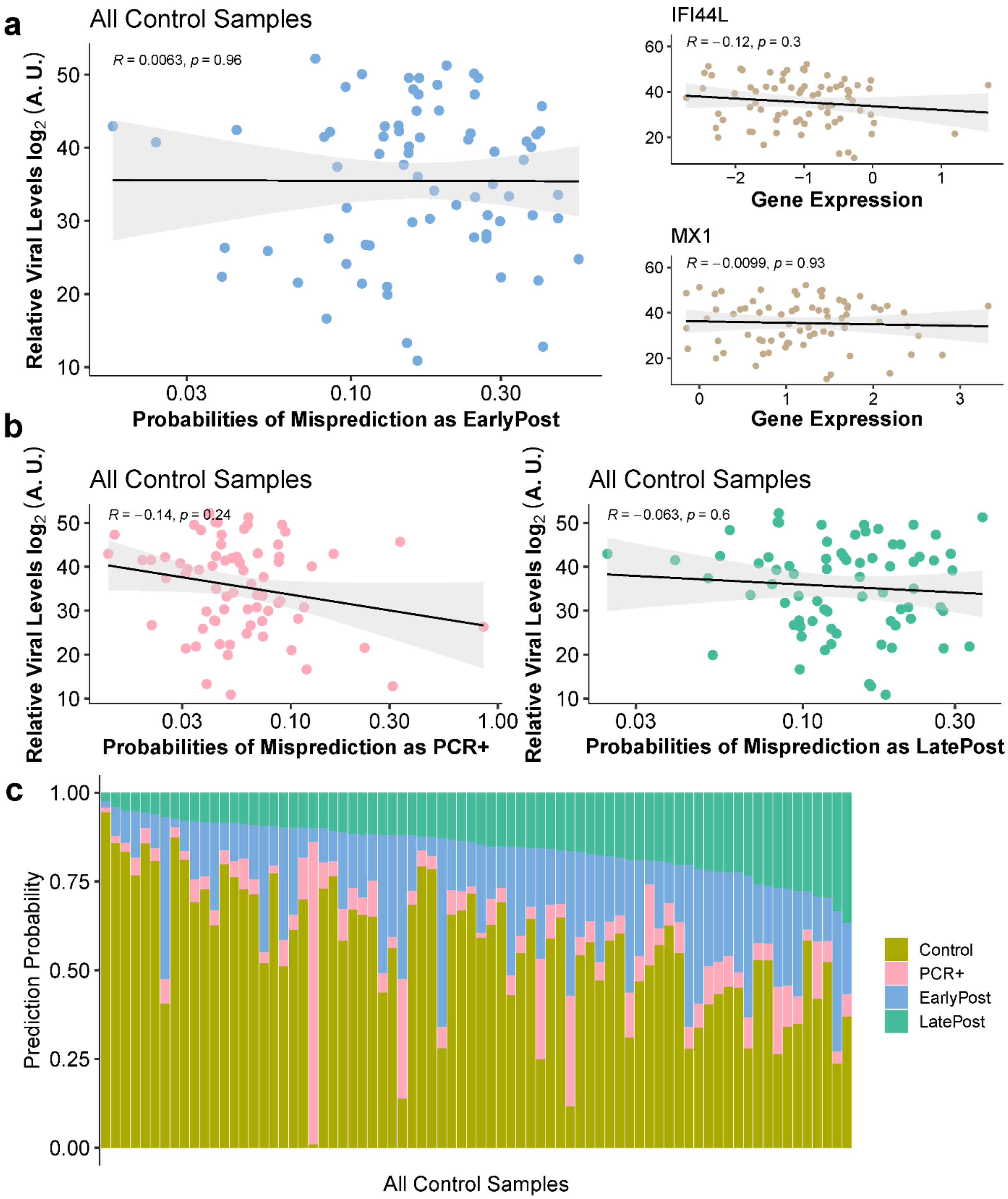

Appendix Figure S3: Gene expression-based multiclass classifier predictions of control samples do not anticipate virus levels. a, Correlation between maximum relative viral level during infection and i) the probabilities of misclassification as EarlyPost (*Left*) using the gene

expression-based multiclassifier model built in the same way as Figure 5a; ii) two upregulated genes, with uncorrected p values (*Right*). A.U., arbitrary units calculated as 80-(minimum cycle threshold PCR result) for each participant. **b**. Correlation plot of maximum relative viral levels measured during infection with the probabilities of misclassification as PCR-positive or LatePost using the classifier from panel a of the control samples prior to infection from the same participant. A.U., arbitrary unit calculated as 80-(minimum cycle threshold PCR result) for each participant. **c**. Prediction probabilities generated by the multiclass classifier for all control samples are shown by bar plot. The results are in increasing order of the prediction probability obtained that each control sample is LatePost.

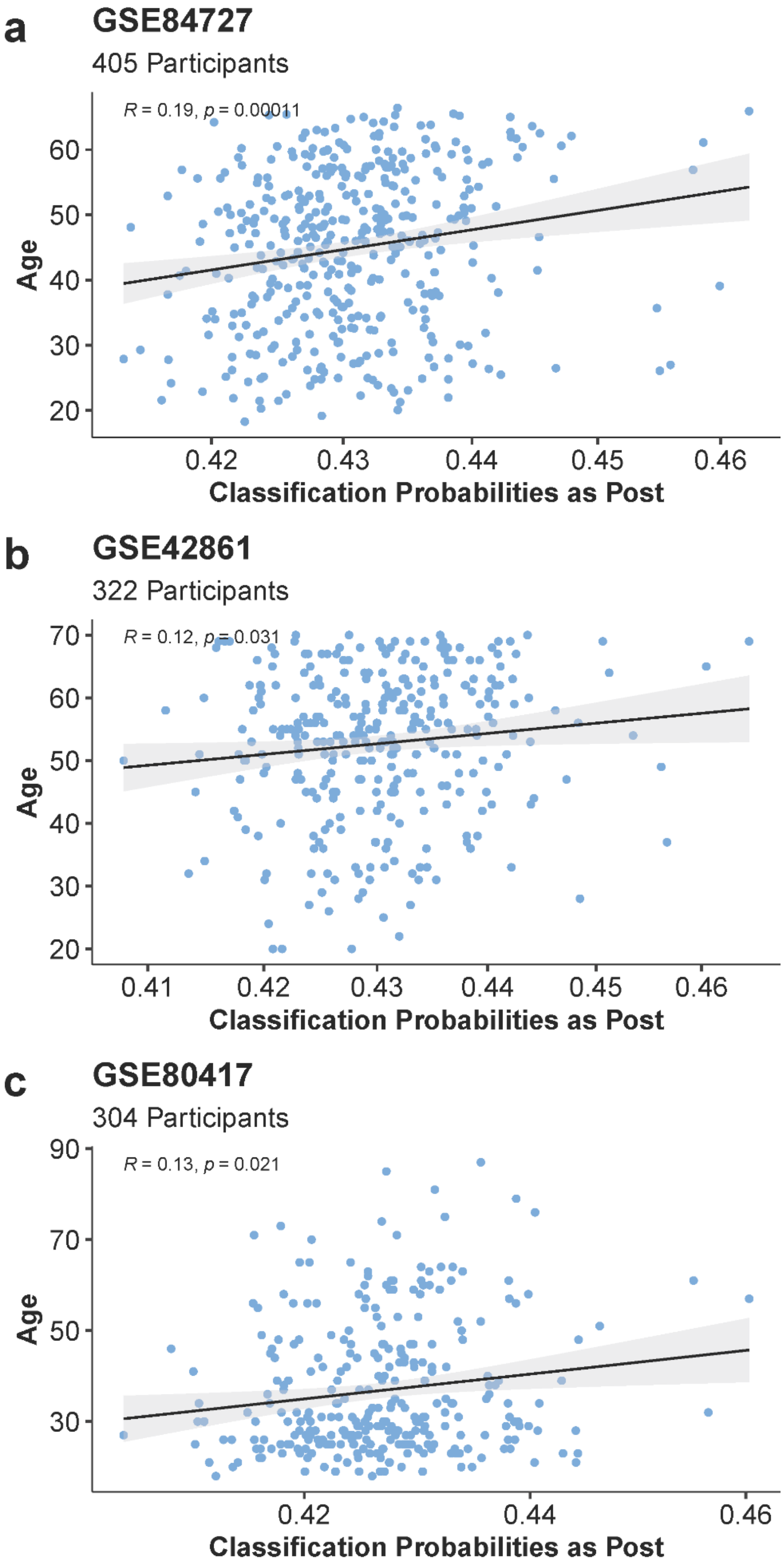
a
GSE84727
405 Participants
R = 0.19, p = 0.00011
Age
60
50
40
30
20
0.42
0.43
0.44
0.45
0.46
Classification Probabilities as Post
b
GSE42861
322 Participants
R = 0.12, p = 0.031
Age
70
60
50
40
30
20
0.41
0.42
0.43
0.44
0.45
0.46
Classification Probabilities as Post
c
GSE80417
304 Participants
R = 0.13, p = 0.021
Age
90
70
50
30
0.42
0.44
0.46
Classification Probabilities as Post

Appendix Figure S4. Post-infection probabilities are associated with age. Applying the multiclass classifier on several cohorts of methylation data shows a significant positive correlation between age and prediction probabilities as Post.

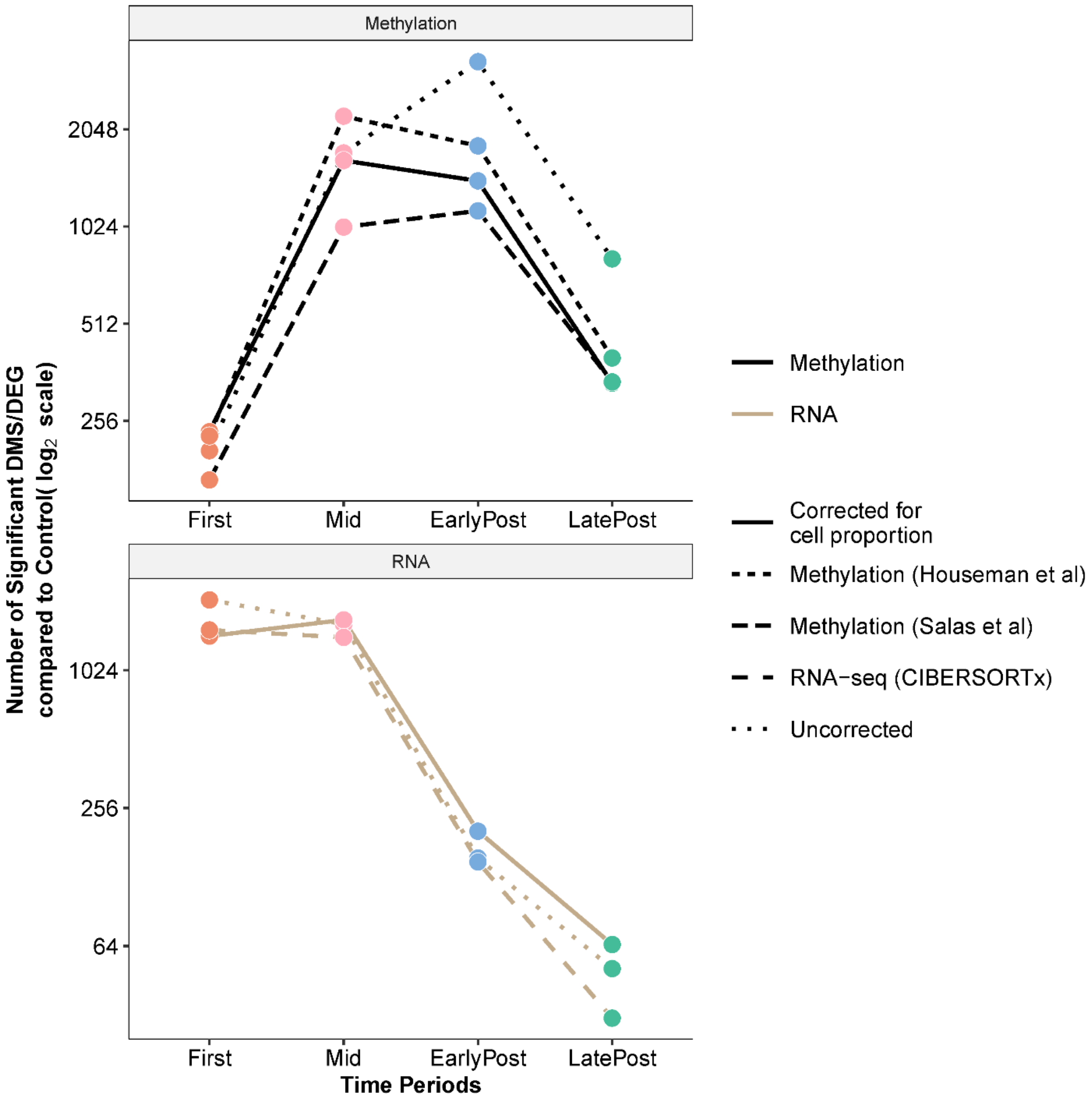

Appendix Figure S5: Timing of methylation and RNA changes using different proportion estimation methods. Number of DMS or DEG in each pseudotime period vs. pre-infection controls (nominal p<10-4). Numbers were either corrected for cell type proportions using the methods indicated (Houseman *et al*, 2012; Newman *et al*, 2019; Salas *et al*, 2022) or uncorrected. See also Figure 1b, Figure EV2 and Appendix Figure S6.

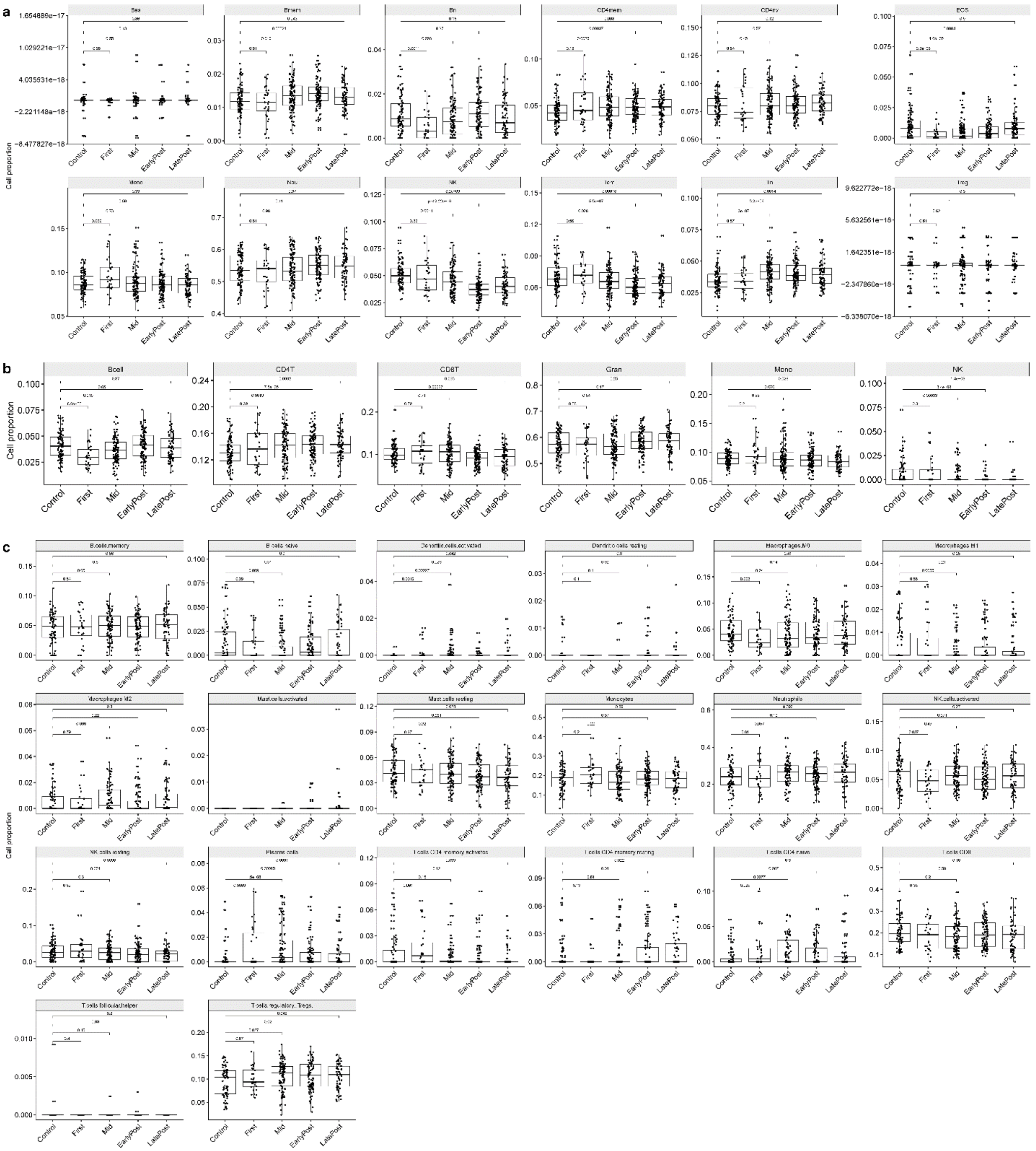

Appendix Figure S6. Visualization of cell proportion differences across time points. Cell type proportion variation across intervals relative to the first PCR positive test in the young adult longitudinal study. Cell type proportions at each period after infection were compared with pre-infection cell type proportions using two-tailed Wilcoxon rank sum test. **a**, Boxplot of inferred

cell type proportions based on all methylation samples and the deconvolution basis was from the Salas et al. (Salas *et al.*, 2022). **b**, Boxplot of inferred cell type proportions based on all methylation samples and the deconvolution basis was from Houseman et al. (Houseman *et al.*, 2012). **c**, Boxplot of inferred cell type proportions based on all RNA-seq samples and the deconvolution basis was the LM22 signature matrix from CIBERSORTx (Newman *et al.*, 2019).

a

Name	Normalization Pipeline	SSE
Raw	No regression	/
B2M2B (current)	Raw → converted to M value → regress out the cell type proportion → converted to beta values.	29850
B_regress	Raw → regress out the cell type proportion directly in the beta value space	30730

b

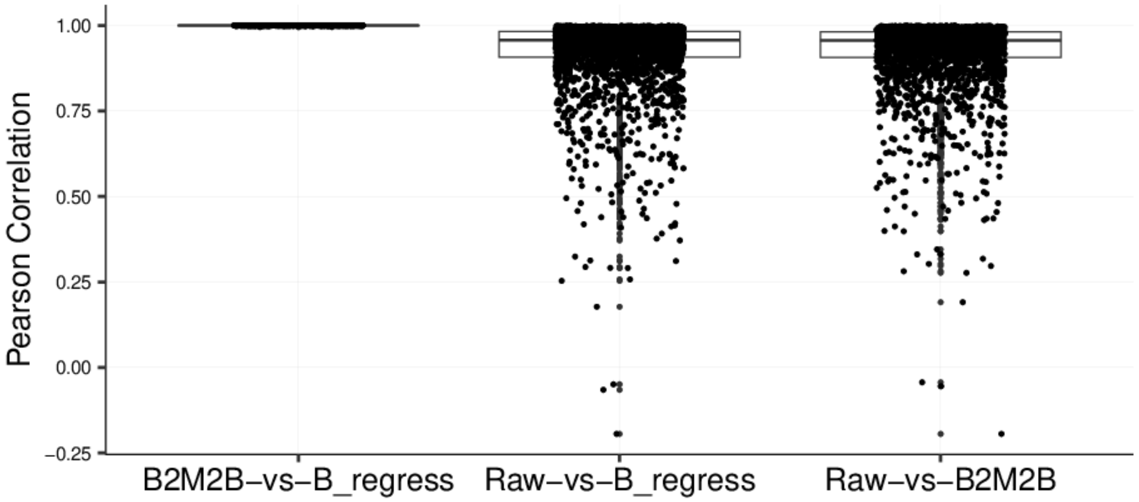

c

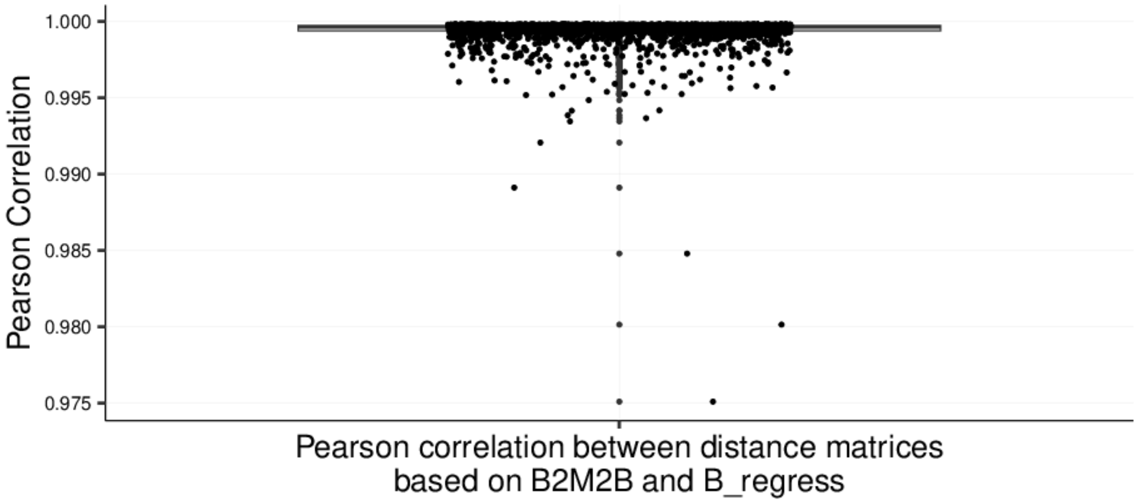

Appendix Figure S7. B2M2B and B_regress are highly correlated as two different normalization approaches to eliminate the cell type proportion variations as confounders. **a**, Detailed normalization flowcharts for B2M2B and B_regress. Total within-cluster variance (SSE) for each approach is provided in the last column. **b**, Pearson correlations between

corresponding methylation samples across all 707,361 CpGs following different normalization pipelines. **c**, Comparison of distance matrices (dynamic warping distance), the measure used for the clustering in Fig. 2A, calculated using B2M2B and B_regress.

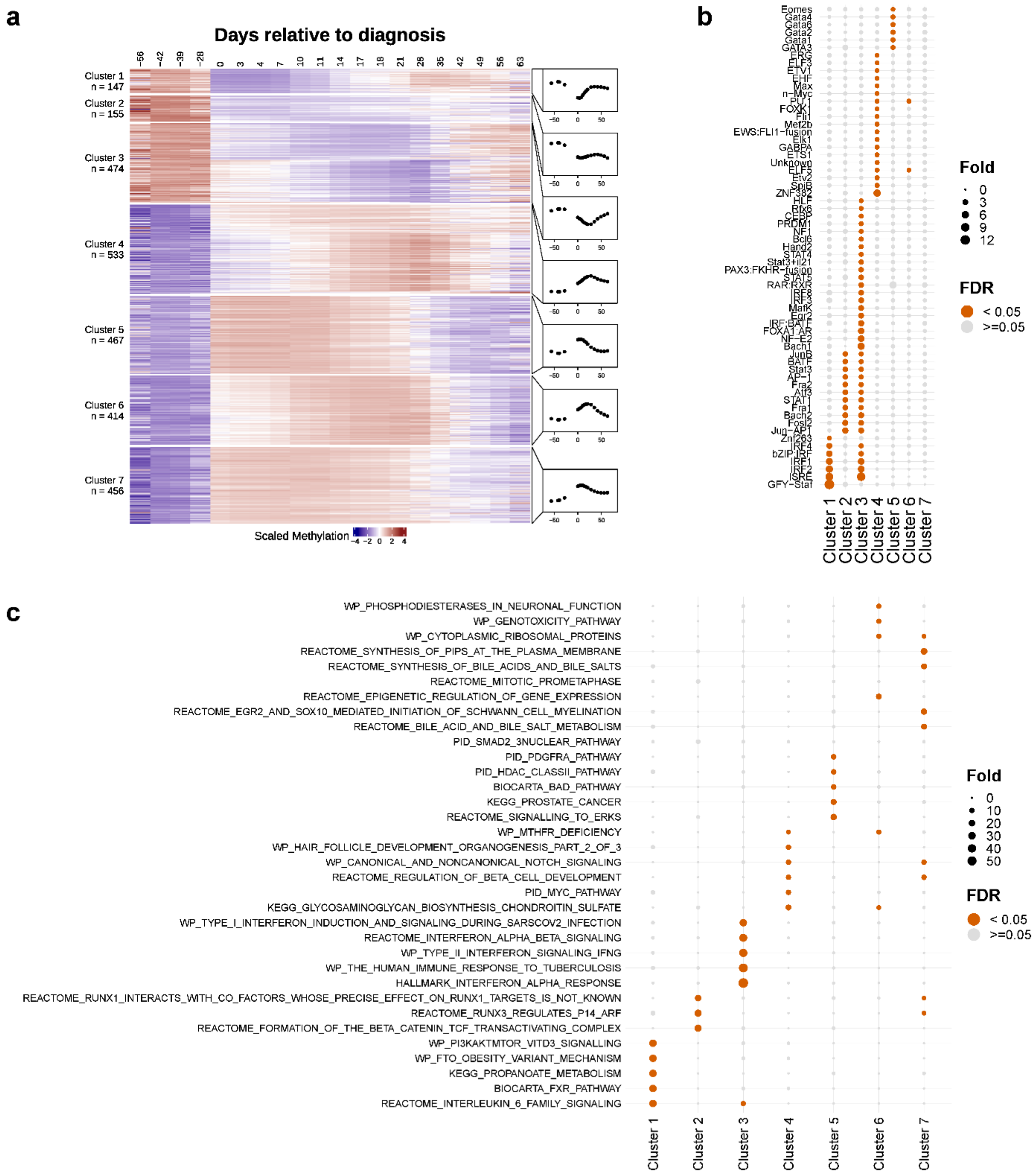

Appendix Figure S8. Characteristics of differential methylation following SARS-CoV-2 infection using B_regress. This is a remake of Figure 2 in the manuscript using the B_regress analysis **a**, Z-scored levels at DMS clustered by temporal trajectory relative to the first PCR-

positive test. Plotted is the average of each cluster over time. **b**, Enrichment of TFBS by cluster within a 200-bp window centered at each DMS, FDR <.05 for at least one cluster. Fold, fold enrichment. **c**, Top five pathways showing enrichment of DMS-associated genes in each cluster. b,c, FDR <.05 for at least one cluster. Fold, fold enrichment. Unlike the results obtained with the B2M2B analysis shown in Fig. 2, the TF enrichment indicates that the results with B_regress are clusters that have less biological coherence. Using B_regress, as shown above, there is less distinction of the TFs between clusters, and several of the clusters in the TF enrichment lack any annotation at all. In the B2M2B analysis shown in main Fig. 2, the clusters are tighter (see Fig. S7A) and are more biologically distinct, as assessed by the TF enrichment analysis.

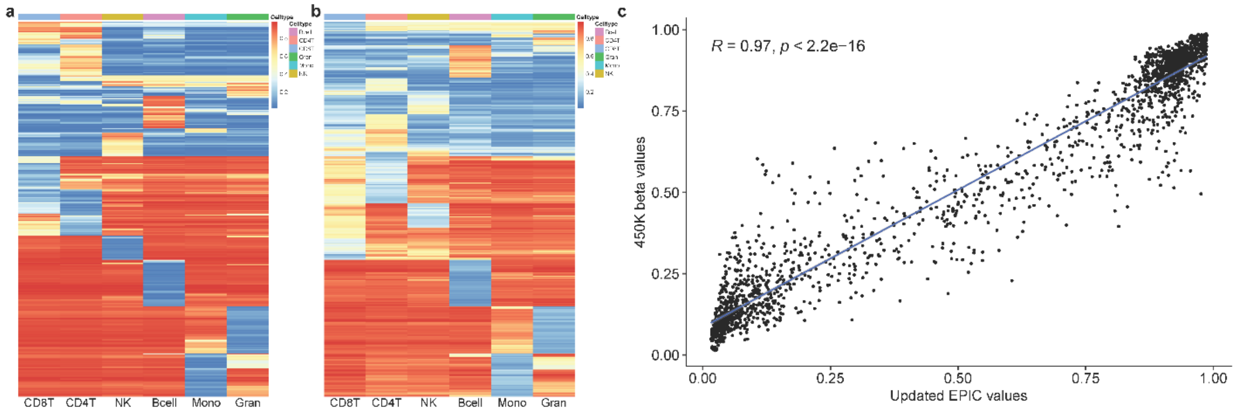

Appendix Figure S9. Updated basis matrix for the EPIC array is highly correlated with the standard Houseman et al. basis. a, Heatmap of the updated basis matrix for the EPIC array. **b**, For comparison, the heatmap using the standard Houseman et al. (Houseman *et al*, 2012) basis for the 450K array is shown. The update basis matrix generates somewhat better cell type discrimination **c**, Scatter plot of the 450K values against the updated EPIC basis values are highly correlated.

2. References

Houseman EA, Accomando WP, Koestler DC, Christensen BC, Marsit CJ, Nelson HH, Wiencke JK, Kelsey KT (2012) DNA methylation arrays as surrogate measures of cell mixture distribution. *BMC Bioinformatics* 13: 86

Newman AM, Steen CB, Liu CL, Gentles AJ, Chaudhuri AA, Scherer F, Khodadoust MS, Esfahani MS, Luca BA, Steiner D *et al* (2019) Determining cell type abundance and expression from bulk tissues with digital cytometry. *Nat Biotechnol* 37: 773-782

Salas LA, Zhang Z, Koestler DC, Butler RA, Hansen HM, Molinaro AM, Wiencke JK, Kelsey KT, Christensen BC (2022) Enhanced cell deconvolution of peripheral blood using DNA methylation for high-resolution immune profiling. *Nat Commun* 13: 761
