# Supplementary material for: A methylation clock model of mild SARS‐CoV‐2 infection provides insight into immune dysregulation
Source: Mol Syst Biol. 2023 Mar 15;19(5):e11361. doi: 10.15252/msb.202211361 (PMC10167476; doi:10.15252/msb.202211361)
Supplement: Supplementary file 2 — Expanded View Figures PDF [file MSB-19-e11361-s001.pdf]

Expanded View Figures

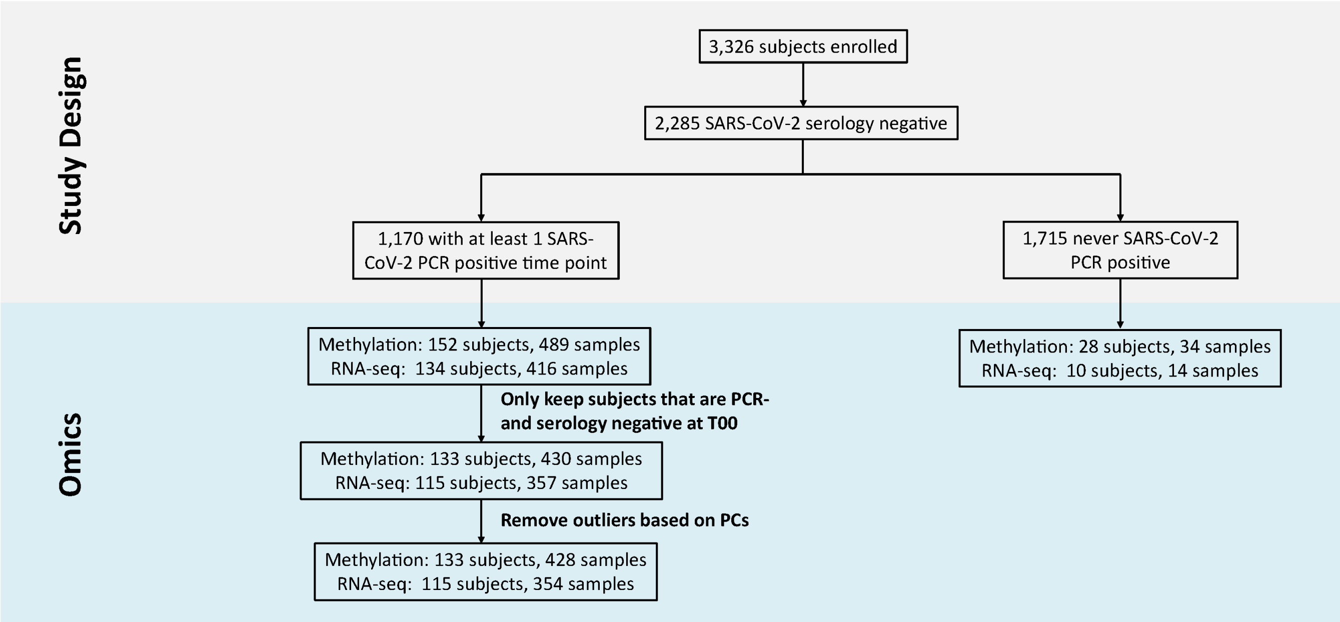

Sample Details

	Subject-wise		Sample-wise	
	**Methylation**	**RNA-seq**	**Methylation**	**RNA-seq**
**Total**	133	115	428	354
**Gender**	Male: 122<br>Female: 11	Male: 104<br>Female: 11	Male: 397<br>Female: 31	Male: 325<br>Female: 29
**Age**	Overall: 19.77 ± 2.45<br>Male: 19.81 ± 2.52<br>Female: 19.36 ± 1.57	Overall: 19.88 ± 2.58<br>Male: 19.93 ± 2.66<br>Female: 19.36 ± 1.57	n.a.	n.a.
**Race**	White: 94<br>Black: 21<br>Multi-racial: 5<br>AI/AN: 3<br>Asian: 3<br>Hawaiian/OPI: 1<br>Other: 1<br>NA: 5	White: 80<br>Black: 19<br>Multi-racial: 4<br>AI/AN: 3<br>Asian: 3<br>Other: 1<br>NA: 5	White: 306<br>Black: 64<br>Multi-racial: 14<br>AI/AN: 11<br>Asian: 8<br>Hawaiian/OPI: 4<br>Other: 3<br>NA: 18	White: 252<br>Black: 55<br>Multi-racial: 10<br>AI/AN: 10<br>Asian: 8<br>Other: 3<br>NA: 16
**Ethnicity**	Non-Hispanic: 57<br>Hispanic: 44<br>NA: 32	Non-Hispanic: 48<br>Hispanic: 39<br>NA: 28	Non-Hispanic: 172<br>Hispanic: 147<br>NA: 109	Non-Hispanic: 136<br>Hispanic: 124<br>NA: 94
**Symptom**	Asymptomatic: 68<br>Symptomatic: 65	Asymptomatic: 60<br>Symptomatic: 55	n.a.	n.a.

***n.a.** Not Applicable; **NA** Not Available

Figure EV1. CHARM study description.

Participants and samples are summarized by gender, race, ethnicity, and reported symptoms. All analyses of methylation changes associated with SARS-CoV-2 infection used preinfection samples as the Control group. The methylation data from the 28 never infected participants were used for the model evaluation of this group shown in Fig 4C. n.a., not applicable; NA, not available.

© 2023 The Authors

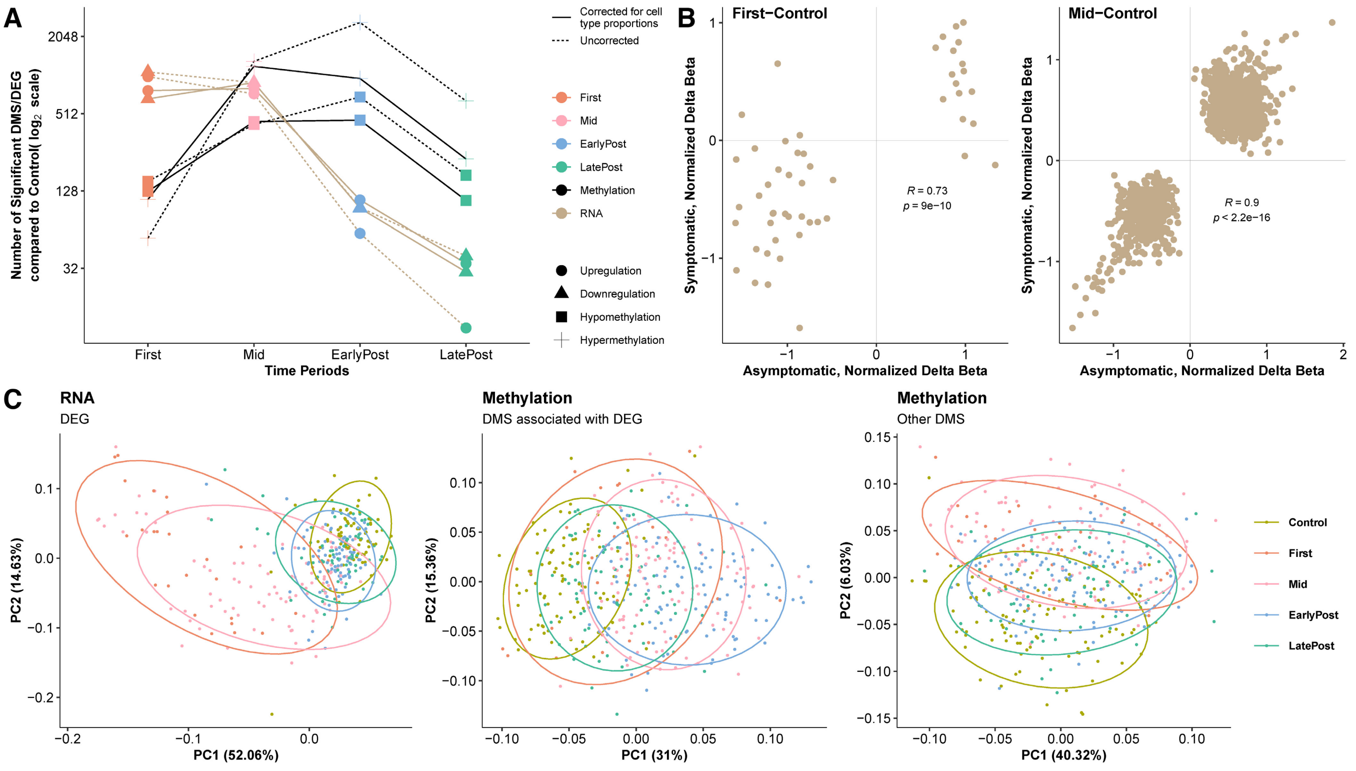

Figure EV2. Relationship of gene and methylation changes following SARS-CoV-2 infection.

A The number of differentially expressed genes (DEG) and differentially methylated sites (DMS) during each infection period compared with preinfection levels (uncorrected $P < 1e^{-4}$) are plotted separately by direction of regulation. Analysis corrected for cell-type proportions and uncorrected are shown separately.

B Scatter plots comparing the changes in methylation levels compared with control following asymptomatic ($n = 68$) and mildly symptomatic ($n = 65$) infections for the First and Mid time period. These plots correspond to the same analysis shown for EarlyPost and LatePost in Fig 1C.

C Principal component analysis of the Mid vs. Control DEG or DMS (with FDR < 0.05 and fold change > 1.5 for DEG) at all time periods. Other DMS, unannotated DMS. These plots correspond to Fig 1D.

© 2023 The Authors

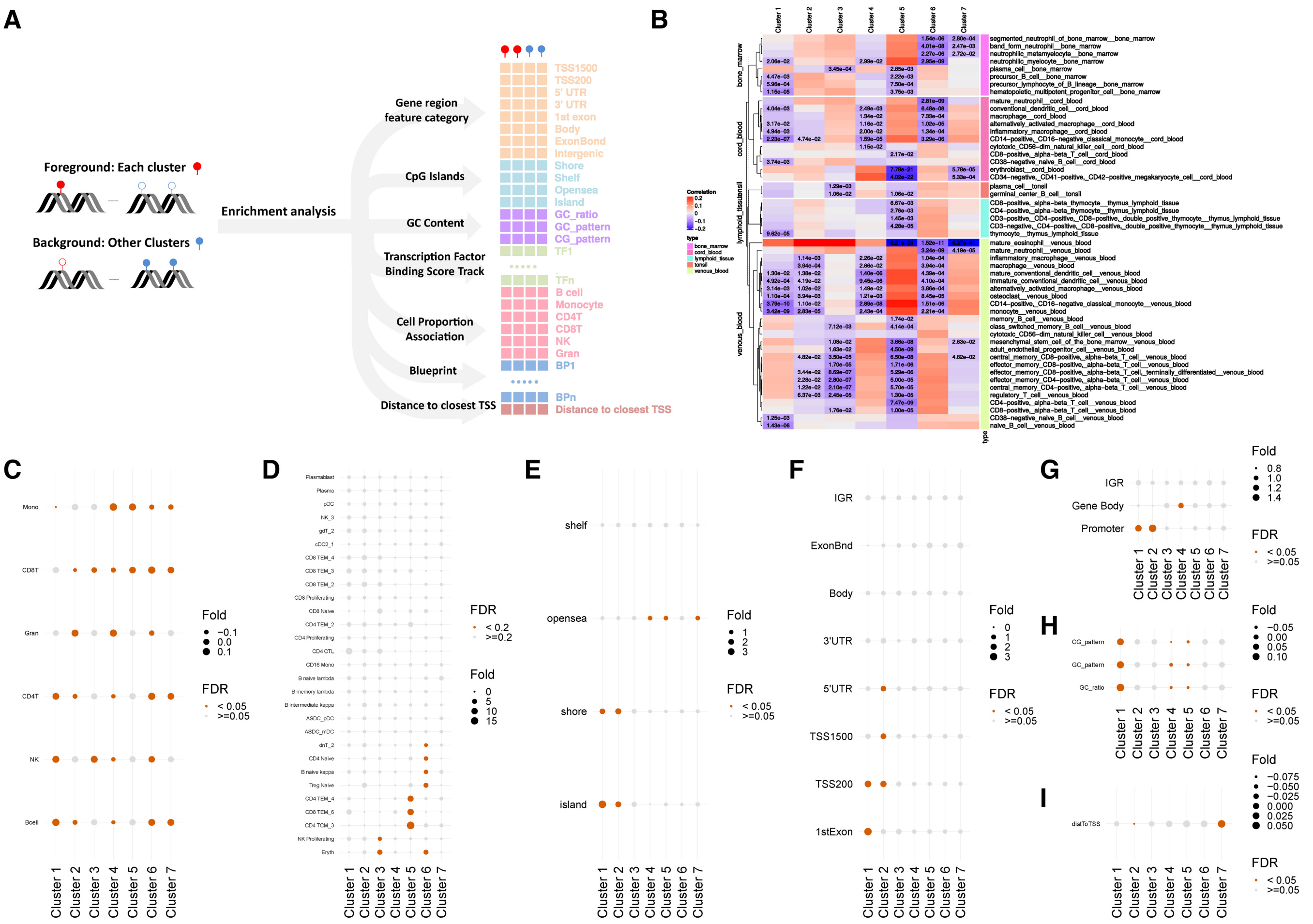

Figure EV3. Analysis of temporal clusters of differentially methylated sites.

A Schematic showing the features evaluated by enrichment analysis for association with postinfection hypomethylated sites in each DMS cluster from Fig 2.

B Correlation of DMS in each cluster with Blueprint cell-type methylation markers. (See Materials and Methods).

C Enrichment analysis with respect to the Pearson correlations of DMS in each cluster with inferred cell-type proportions. Fold enrichment for each cluster is indicated in comparison with all clusters (see Materials and Methods).

D Enrichment analysis showing the top five enriched cell markers from single-cell RNA-seq for DMS in each cluster. Cell markers with FDR < 0.05 are highlighted.

E Enrichment analysis for CpG island categories. Results with FDR < 0.05 are highlighted.

F Enrichment analysis for gene region feature categories. Results with FDR < 0.05 are highlighted.

G Enrichment analysis of gene region feature categories aggregated into promoter region (TSS1500, TSS200, 1st Exon, 5'UTR) and gene body (3'UTR, Body, ExonBnd). IGR is also included. Results with FDR < 0.05 are highlighted.

H Enrichment analysis of CG and GC content categories. Results with FDR < 0.05 are highlighted.

I Enrichment analysis of distance to transcription start site (TSS). Results with FDR < 0.05 are highlighted.

Figure EV4. Data normalization and modeling procedures.

A Schematic of the processing pipeline used for RNA-Seq data normalization.

B Schematic of the processing pipeline used for Methylation data normalization.

C Schematic of the procedure utilized for nested cross-validation of all machine learning models generated. The left panel indicates one outer iteration for developing the model M built from the training set. The right side gives the data summary derived from all outer iterations.

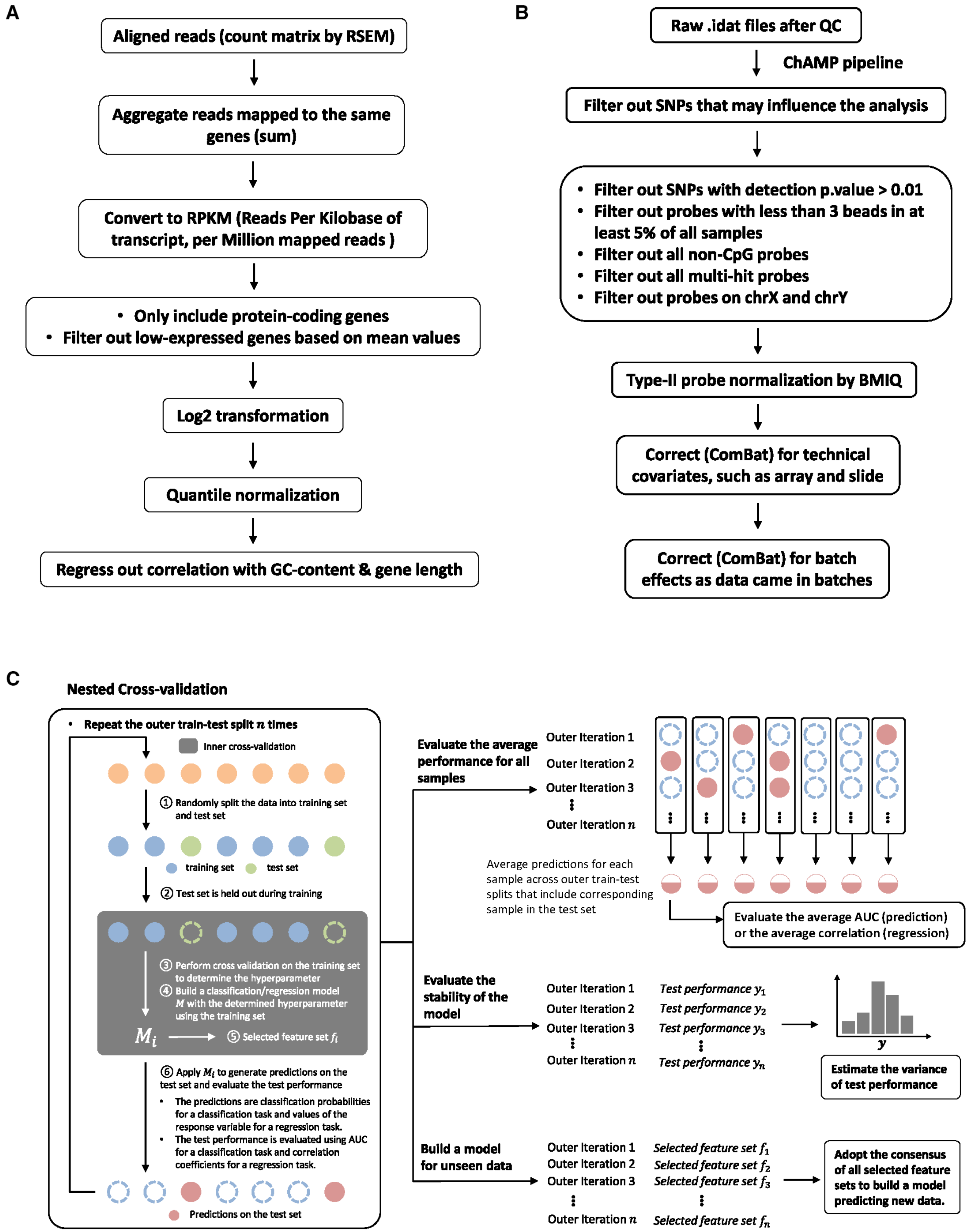

Figure EV4.

© 2023 The Authors

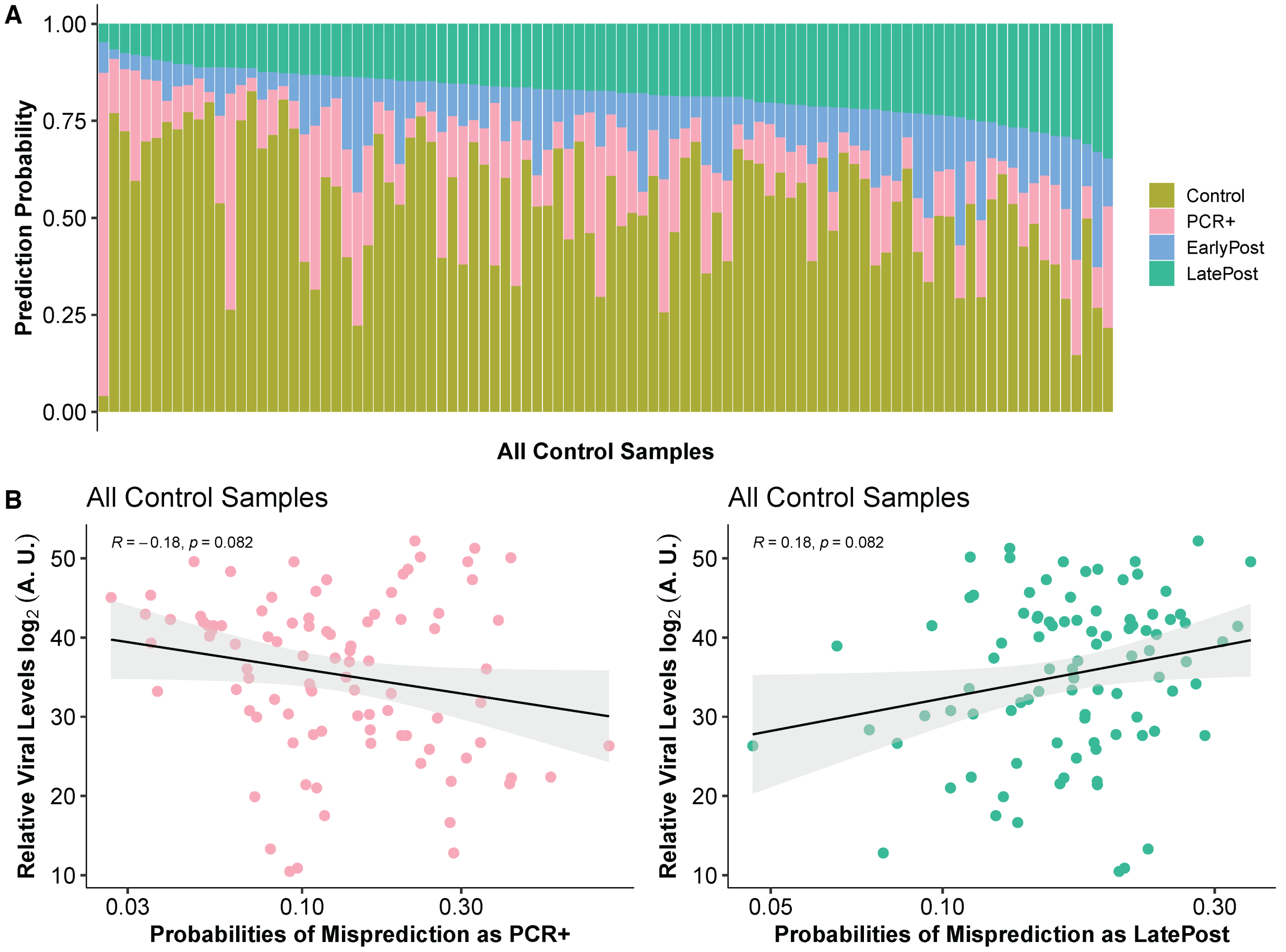

Figure EV5. Multiclass classifier predictions of control samples anticipate virus levels.

A Prediction probabilities generated by the multiclass classifier for all control samples are shown by bar plots. The results are in increasing order of the prediction probability obtained that each control sample is LatePost.

B Correlation plot of maximum relative viral levels measured during infection with the probabilities of misclassification as PCR-positive or LatePost using the classifier from Fig 3D of the control samples prior to infection from the same participants. A.U., arbitrary units calculated as 80-(minimum cycle threshold PCR result) for each participant.

© 2023 The Authors
