# Supplementary material for: A methylation clock model of mild SARS‐CoV‐2 infection provides insight into immune dysregulation
Source: Mol Syst Biol. 2023 Mar 15;19(5):e11361. doi: 10.15252/msb.202211361 (PMC10167476; doi:10.15252/msb.202211361)
Supplement: Supplementary file 14 — PDF+ [file MSB-19-e11361-s006.pdf]

Article

TRANSPARENT PROCESS
OPEN ACCESS

molecular systems biology

A methylation clock model of mild SARS-CoV-2 infection provides insight into immune dysregulation

Weiguang Mao[1,‡], Clare M Miller[2], Venugopalan D Nair[2], Yongchao Ge[2], Mary Anne S Amper[2], Antonio Cappuccio[2], Mary-Catherine George[2], Carl W Goforth[3], Kristy Guevara[2], Nada Marjanovic[2], German Nudelman[2], Hanna Pincas[2], Irene Ramos[2], Rachel S G Sealfon[4], Alessandra Soares-Schanoski[2,§], Sindhu Vangeti[2], Mital Vasoya[2], Dawn L Weir[3], Elena Zaslavsky[2], Biobank Team[5,6,†], Seunghee Kim-Schulze[5,6], Sacha Gnjatic[5,6], Miriam Merad[5,6], Andrew G Letizia[3], Olga G Troyanskaya[4,7,8], Stuart C Sealfon[2,*] & Maria Chikina[1,**]

Abstract

DNA methylation comprises a cumulative record of lifetime exposures superimposed on genetically determined markers. Little is known about methylation dynamics in humans following an acute perturbation, such as infection. We characterized the temporal trajectory of blood epigenetic remodeling in 133 participants in a prospective study of young adults before, during, and after asymptomatic and mildly symptomatic SARS-CoV-2 infection. The differential methylation caused by asymptomatic or mildly symptomatic infections was indistinguishable. While differential gene expression largely returned to baseline levels after the virus became undetectable, some differentially methylated sites persisted for months of follow-up, with a pattern resembling autoimmune or inflammatory disease. We leveraged these responses to construct methylation-based machine learning models that distinguished samples from pre-, during-, and postinfection time periods, and quantitatively predicted the time since infection. The clinical trajectory in the young adults and in a diverse cohort with more severe outcomes was predicted by the similarity of methylation before or early after SARS-CoV-2 infection to the model-defined postinfection state. Unlike the phenomenon of trained immunity, the postacute SARS-CoV-2 epigenetic landscape we identify is antiprotective.

Keywords DNA methylation; machine learning model; SARS-CoV-2; temporal dynamics; trained immunity

Subject Categories Chromatin, Transcription & Genomics; Immunology; Microbiology, Virology & Host Pathogen Interaction

DOI 10.15252/msb.202211361 | Received 23 September 2022 | Revised 16 February 2023 | Accepted 20 February 2023 | Published online 15 March 2023

Mol Syst Biol. (2023) 19: e11361

Introduction

An individual's pattern of DNA methylation contains a lifetime record of environmental exposures and has been associated with increased risk for various autoimmune, neurological, and metabolic diseases. Methylation-based signatures have been reported to have higher predictive value for future health outcomes than polygenic risk scores (preprint: Thompson *et al*, 2022; Yousefi *et al*, 2022). DNA methylation has been used to construct lifelong methylation clocks that predict chronological age and all-cause mortality (Horvath & Raj, 2018; Lu *et al*, 2019). While methylation has been linked to diverse phenotypes in association studies, densely sampled longitudinal data that capture intraindividual methylation changes have been limited (Furukawa *et al*, 2016; Chen *et al*, 2018).

1 Department of Computational and Systems Biology, School of Medicine, University of Pittsburgh, PA, Pittsburgh, USA
2 Department of Neurology, Icahn School of Medicine at Mount Sinai, NY, New York, USA
3 Naval Medical Research Center, MD, Silver Spring, USA
4 Center for Computational Biology, Flatiron Institute, Simons Foundation, NY, New York, USA
5 Precision Immunology Institute, Icahn School of Medicine at Mount Sinai, NY, New York, USA
6 Human Immune Monitoring Center (HIMC), Icahn School of Medicine at Mount Sinai, NY, New York, USA
7 Department of Computer Science, Princeton University, NJ, Princeton, USA
8 Lewis-Sigler Institute for Integrative Genomics, Princeton University, NJ, Princeton, USA
*Corresponding author. Tel: +1 347 866 6317; E-mail: stuart.sealfon@mssm.edu
**Corresponding author. Tel: +1 412 648 3333; E-mail: mchikina@pitt.edu
†Biobank Team members are presented in Appendix 1.
‡Present address: Center for Computational Biology, Flatiron Institute, Simons Foundation, New York, NY, USA
§Present address: Ragon Institute of MGH, MIT, and Harvard, Cambridge, MA, USA

© 2023 The Authors. Published under the terms of the CC BY 4.0 license.

Here, we investigate methylation patterns and dynamics during asymptomatic and mildly symptomatic SARS-CoV-2 infection in healthy young adults. While alterations in blood DNA methylation have been reported after symptomatic SARS-CoV-2 infections (Balnis *et al*, 2021; Castro de Moura *et al*, 2021; Corley *et al*, 2021; Konigsberg *et al*, 2021; Zhou *et al*, 2021), our prospective longitudinal study captures the dynamics of methylation changes following asymptomatic infection, giving insights into the long-term memory of environmental exposure and potential disease associations.

Results

Methylome changes after infection

The prospective COVID-19 Health Action Response for Marines (CHARM) study enrolled new US Marine recruits at the beginning of training between May 11 and September 7, 2020. Study participants were assessed periodically, including testing for SARS-CoV-2 by nasal swab PCR and blood sampling during an initial 2-week supervised quarantine and subsequent basic training (Letizia *et al*, 2021; Fig 1A and see Materials and Methods). The cohort was predominantly of European ancestry, male, and physically fit, with an average age of 19.77 ± 2.45 years (Fig EV1). We analyzed longitudinal blood transcriptome and methylome data obtained from 133 recruits who became infected during the study. All infections were either mildly symptomatic (n = 65) or asymptomatic (n = 68), and none required hospitalization.

The blood samples were grouped relative to the day of first diagnosis into the following periods (see Fig 1A): (i) Control (preinfection), (ii) PCR$^+$, which included First (time of first PCR-positive test) and Mid (period of subsequent PCR-positive tests), (iii) EarlyPost (virus clearance indicated by PCR-negative tests continuing up to 45 days from First), (iv) LatePost (PCR-negative tests more than 45 days from First). Several thousand differentially expressed genes (DEG) were seen at the time of first diagnosis compared with preinfection control levels (Dataset EV1). The number of DEG detected at EarlyPost vs. Control was greatly reduced, and few were detected by LatePost. The total number of differentially methylated sites (DMS) in blood DNA peaked later than the DEG, and a large number of DMS were still observed in the periods after PCR positivity (Fig 1B). Changes in blood cell-type proportions occur during SARS-CoV-2 infection (Liu *et al*, 2020), which may affect the detection of DEG and DMS. Computational cell-type deconvolution of both the RNA-seq and methylation data showed concordant changes in the predicted proportions of B cells, T cell subtypes, and NK cells following infection (Appendix Fig S1). The number of DEG and DMS detected over time were similar when analyzing raw data, when correcting for changes in cell-type proportions, and when summarizing up- and downregulation events separately (Figs 1B and EV2A, and Datasets EV1–EV4). These conclusions were robust to changes in the computational framework used to infer cell proportions (Appendix Figs S5 and S6). However, we cannot exclude the possibility that some of the differences we observe correspond to changes in the frequency of a cell type that is not accounted for in computational cell-type deconvolution methods. Comparison of gene expression and methylation levels between the asymptomatic and symptomatic subgroups at each time period showed a maximum of one DEG at false discovery rate (FDR) < 0.05, no significant methylation differences, and high correlation between the level of regulation (normalized delta beta values, Figs 1C and EV2B, and Datasets EV5 and EV6). Because the molecular responses following mildly symptomatic and asymptomatic infections in this cohort were indistinguishable, these groups were combined for all subsequent analyses. We next examined the changes of the genes and methylation sites that were significantly altered at Mid compared with Control. When these gene and methylation levels were plotted at all time periods, the genes overlapped with Control levels following clearance of the virus (Figs 1D and E, and EV2C). By contrast, the methylation changes were more prolonged both for sites associated with DEG and for sites not associated with DEG (Figs 1D and E, and EV2C).

Methylation site dynamics

When the methylation levels of all DMS were aligned by day relative to the initial PCR-positive test and clustered hierarchically using dynamic time-warping distance, three hypomethylation (Clusters 1–3) and 4 hypermethylation (Clusters 4–7) trajectories were observed (Fig 2A). To evaluate whether the clusters distinguished by time trajectories could reflect different mechanisms, we assessed enrichment for various properties (See schematic, Fig EV3A), including nearby transcription factor binding sites (TFBS), pathways, Blueprint Epigenome project cell-type signatures (Stunnenberg *et al*, 2016), cell-type proportions, association with single-cell sequencing-derived cell-type markers, CpG island categories, gene region feature categories, CG/GC content, and distance to transcription start site (Fig EV3B–I). When the 200-bp regions centered on the DMS in each cluster were analyzed for TFBS enrichment using the HOMER motif database (Duttke *et al*, 2019), each of the three hypomethylation clusters and three of the four hypermethylation clusters showed enrichment of distinct TFBS for each cluster (Fig 2B). We also found that the DMS in each cluster was enriched in Blueprint cell-type markers (Fig EV3B). Among the hypomethylated clusters, early changes were generally associated with myeloid cell signatures and later changes with mature lymphocytes (Fig EV3B). Cluster 3, which contained sites showing prolonged hypomethylation, was enriched in mature B cell lineage signatures, including plasma and germinal center cells (Fig EV3B). This finding was concordant with the TFBS enrichment analysis, which showed the association of Cluster 3 with the germinal center regulator BCL6 (see Fig 2B). In addition, the genes annotated to the DMS in each dynamical cluster were enriched for specific MSigDB canonical (Liberzon *et al*, 2011) and hallmark (Liberzon *et al*, 2015) pathways (Fig 2C). These findings indicate that the temporal dynamics clusters are biologically coherent, and suggest that the regulation of DMS within each cluster involves the activation of different pathways and relies on distinct sets of transcription factors that contribute to the targeting of the methylation regulatory machinery.

SARS-CoV-2 methylation clock

We next investigated the potential for DNA methylation dynamics to predict the time since infection. We used a nested cross-validation procedure to generate an elastic net regression model trained on the methylation data to predict the day since infection. The training procedure for all modeling is shown schematically in Fig EV4C. Model

 © 2023 The Authors

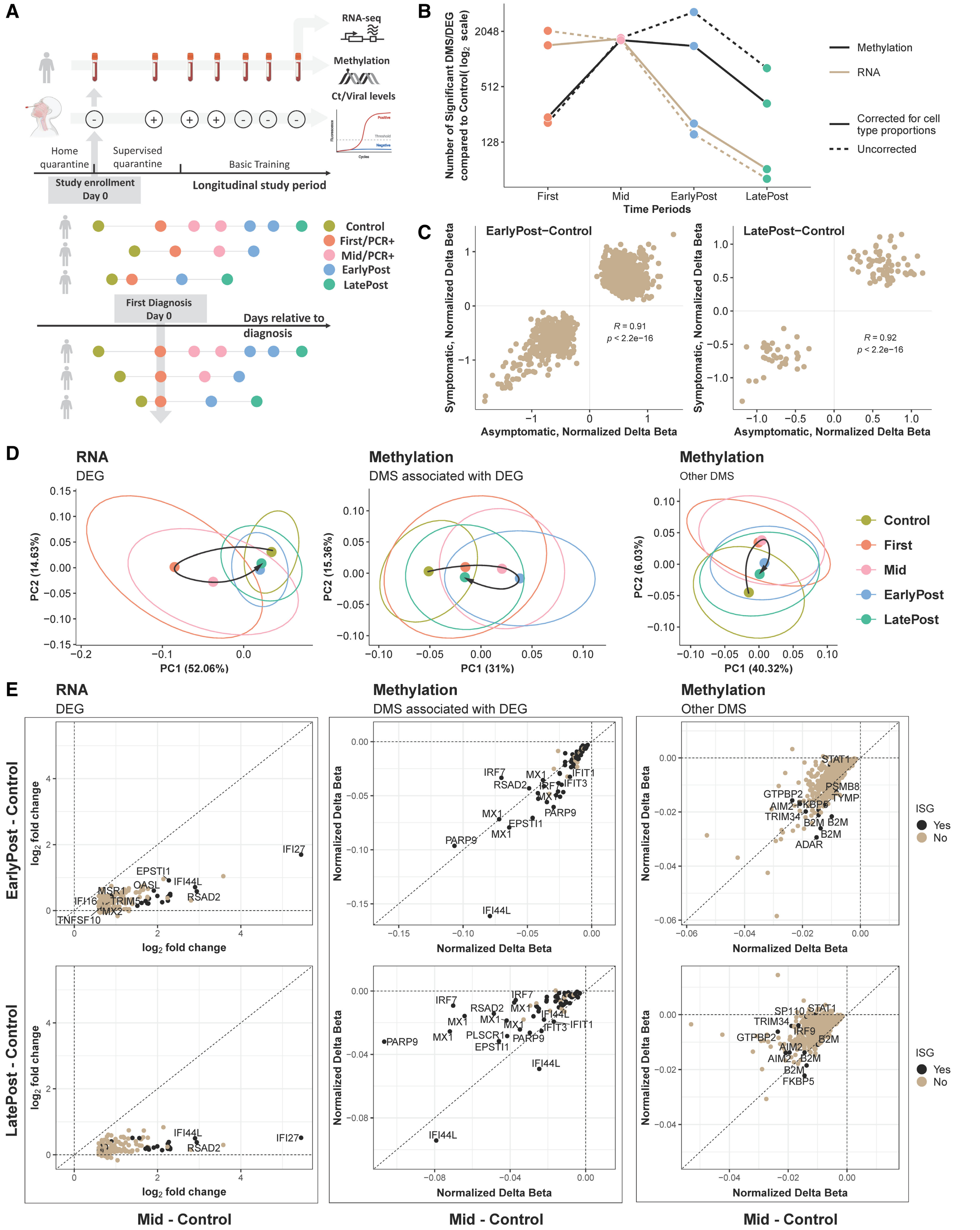
A
RNA-seq
Methylation
Ct/Viral levels
Home quarantine
Supervised quarantine
Basic Training
Study enrollment Day 0
Longitudinal study period
Control
First/PCR+
Mid/PCR+
EarlyPost
LatePost
First Diagnosis Day 0
Days relative to diagnosis
B
Number of Significant DMS/DEG compared to Control(log2 scale)
Time Periods
First
Mid
EarlyPost
LatePost
Methylation
RNA
Corrected for cell type proportions
Uncorrected
C
EarlyPost−Control
LatePost−Control
Symptomatic, Normalized Delta Beta
Asymptomatic, Normalized Delta Beta
R = 0.91
p < 2.2e−16
R = 0.92
p < 2.2e−16
D
RNA
DEG
Methylation
DMS associated with DEG
Methylation
Other DMS
PC1 (52.06%)
PC2 (14.63%)
PC1 (31%)
PC2 (15.36%)
PC1 (40.32%)
PC2 (6.03%)
Control
First
Mid
EarlyPost
LatePost
E
RNA
DEG
Methylation
DMS associated with DEG
Methylation
Other DMS
EarlyPost - Control
LatePost - Control
log2 fold change
Normalized Delta Beta
ISG
Yes
No
Mid - Control
Mid - Control
Mid - Control

Figure 1.

Figure 1. Prolonged blood DNA methylation changes in asymptomatic and mild SARS-CoV-2 infections.

A Schematic of the SARS-CoV-2 study design and alignment of human subjects by infection timing. Examples of 3 subject trajectories are shown arranged by study time (top) and infection pseudotime, aligned by diagnosis (bottom).

B Number of DMS or DEG in each pseudotime period vs. preinfection controls (nominal $P < 10^{-4}$). Numbers were either corrected for cell-type proportions or uncorrected. See Fig EV2.

C Scatter plots of differential methylation at the sites in (B) for asymptomatic ($n = 68$) vs. mild ($n = 65$) infections. For each differential contrast, we first selected DMS in Fig 1B (all subjects) that were also differentially methylated (FDR < 0.05) within symptomatic or asymptomatic groups. See Fig EV2B.

D Principal component analysis of the Mid vs. Control DEG or DMS (with FDR < 0.05 and fold change > 1.5 for DEG) at all time periods. Other DMS, are DMS that do not map to a DEG. We note that for gene expression, Post time points are very close to Control, while this is not the case for methylation. Moreover, the pattern is similar for DEG-associated and other differential probes.

E Scatter plots of differential expression (log2 fold change) or methylation (normalized delta beta) at the indicated periods for the DEG and DMS in (D). Performed assays were RNA-seq and methylation microarray, and the limma method was applied for differential analysis of either dataset.

predictions were highly correlated with the actual day since infection (Fig 3A). To examine the accuracy of methylation-based prediction over time and to determine the sites most important for predictions at different postinfection periods, we trained separate models on all CpG sites for samples from different time windows and determined which sites were most often selected by 100 model iterations for each window. The models showed predictive power for all five-time windows examined (Fig 3A) The most important methylation sites for predicting different time windows showed little overlap, indicating that the methylation patterns continue to evolve months after the initial infection (Fig 3B). We next examined the accuracy of binary classification models to distinguish between pairs of Control, PCR-positive, EarlyPost, and LatePost periods (Fig 3C). The models for distinguishing preinfection and postinfection groups showed the highest accuracy, and all iterations for all classification problems performed above chance. We constructed a multiclass classifier that assigned each sample to its time period with high accuracy, ranging from an area under the receiver operator curve (AUC) of 0.88 for the two Post periods to 0.96 for Control (Fig 3D). One limitation of our study is that most participants were male. To determine whether these analyses were applicable to females, we examined the classification of the male and female participants in our dataset separately. We compared the multiclass classifier performance in the 31 samples from the 11 female participants (Appendix Fig S2A) and in the 397 samples from the 122 male participants (Appendix Fig S2B). Overall, the samples from both sexes were classified with similar accuracy, although the confidence intervals for females were wide due to small sample size.

Relationship to other conditions

In order to characterize the specificity and generalizability of methylation changes observed during SARS-CoV-2 infection we examined whether a model trained to distinguish post PCR^+ samples (EarlyPost and LatePost combined) from Control could also distinguish other conditions associated with altered immunological states. Between mid-April and mid-May 2020, an outbreak of SARS-CoV-2 occurred in several companies during basic training at Parris Island, SC. Although few cases were confirmed by PCR testing, a retrospective serological study of exposed recruits was performed (Sah *et al*, 2021). Using DNA methylation from samples obtained in mid-July 2020 about 10 weeks after exposure, from 71 seropositive and 20 seronegative recruits, the model assignment of Control and post PCR^+ correlated with serological status (receiver operator curve AUC = 0.7, FDR = 0.016; Fig 4A and B, and Dataset EV7). This indicates that seropositive and seronegative recruits who were exposed to SARS-CoV-2 can be distinguished retrospectively by their methylation states. Most of the infected recruits in the longitudinal study were first PCR-positive following the 2-week supervised quarantine and the first few weeks of basic training. Using longitudinal samples from recruits who remained PCR-negative at a time of training control study, we found that the model did not distinguish the quarantine and basic training samples (Fig 4A).

We next examined the classification of samples from infections and inflammatory diseases (Dataset EV8). We found that the model did not distinguish samples from before and 4 weeks after H3N2 influenza challenge (Fig 4A, and Datasets EV9 and EV10). Significant classification accuracy was obtained in distinguishing control samples in each dataset from systemic lupus erythematosus (SLE), multiple sclerosis, chronic hepatitis C virus infection, rheumatoid arthritis, inflammatory bowel disease, and hepatitis C virus infection, as well as for high vs. low levels of chronic human immunodeficiency virus infections (Fig 4A and B). Significant accuracy was not achieved for classifying asthma, Sjogren's syndrome, respiratory allergies, tuberculosis infection, and chronic obstructive pulmonary disease (Fig 4A). To further examine the relationship of the postinfection methylation state induced by SARS-CoV-2 to that associated with other diseases, we determined the enrichment of postinfection DMS in the CHARM study to those reported in studies of other diseases. Significant enrichment was observed between EarlyPost period DMS and the HCV study, an HIV study, and two SLE studies (Fig 4C). The LatePost DMS was significantly enriched in one of the two SLE studies (Fig 4D). Comparing the postinfection SARS-CoV-2 DMS and the studies showing enrichment by order of significance of DMS showed a high overlap between the DNA hypomethylation sites in SARS-CoV-2 and those in SLE (Fig 4E). Seven of the eight most significant EarlyPost DMS that were assayed in either of two SLE datasets were included in the top 10 DMS identified in the SLE methylation studies, and six of the most significant LatePost DMS were among the 14 most significant sites identified in one of the SLE studies (Fig 4E).

Overall, we find that our methylation model has considerable overlap with other inflammatory conditions including chronic infection and autoimmune diseases and is most similar to SLE. This is consistent with the observation that the changes we observe are related to the modulation of interferon signaling, which is activated in SLE (Ronnblom & Leonard, 2019).

Immunological effects of prolonged methylation pattern and relevance to a more diverse cohort

Epigenetic regulation following infection has in some instances been found to convey protection against subsequent infection challenge,

 © 2023 The Authors

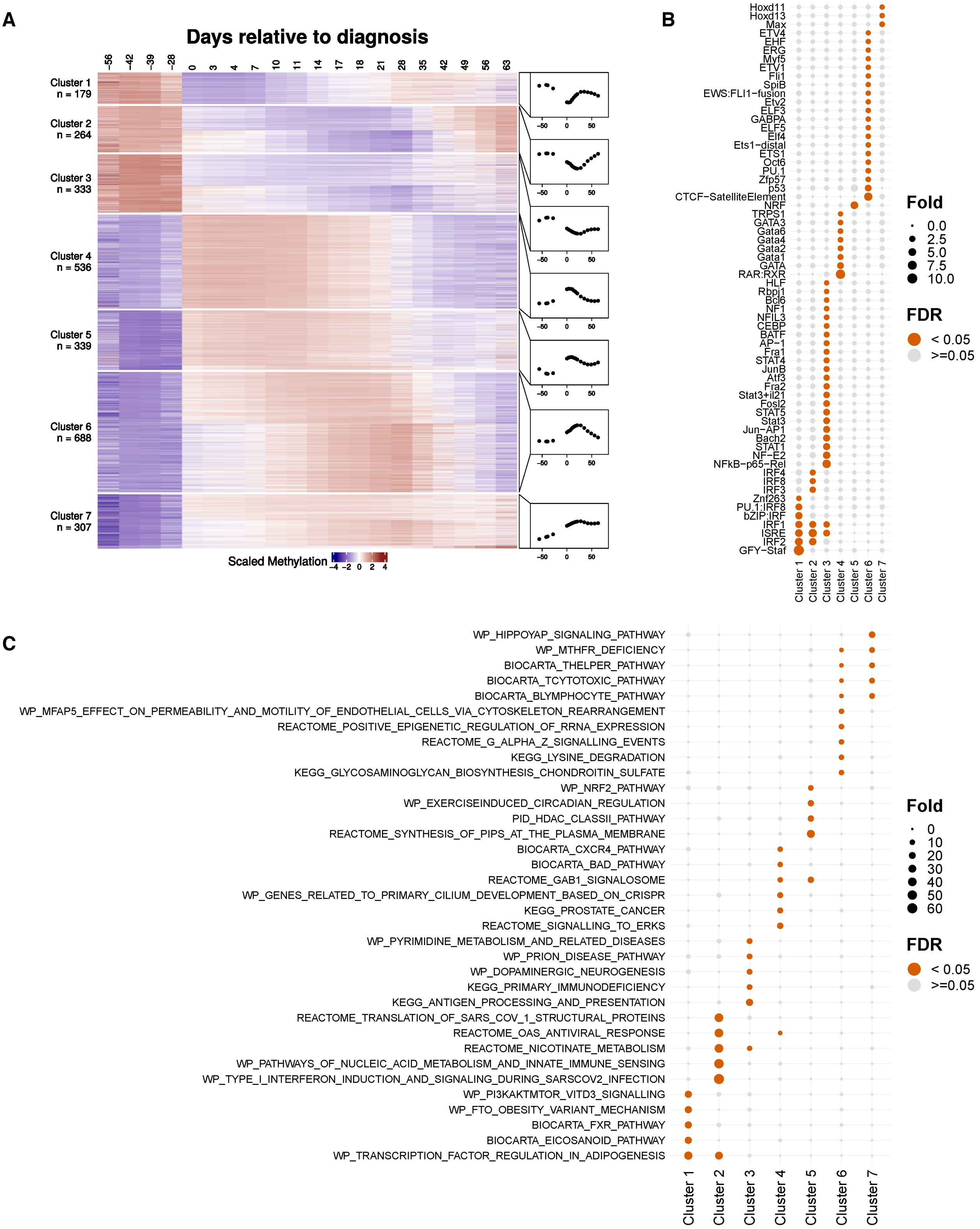

Figure 2. Characteristics of differential methylation following SARS-CoV-2 infection.

A Z-scored levels at DMS clustered by temporal trajectory relative to the first PCR-positive test. Plotted is the average of each cluster over time.

B Enrichment of TFBS by cluster within a 200-bp window centered at each DMS.

C Top five pathways showing enrichment of DMS-associated genes in each cluster. (B, C) FDR $<$ 0.05 for at least one cluster. Fold = fold enrichment.

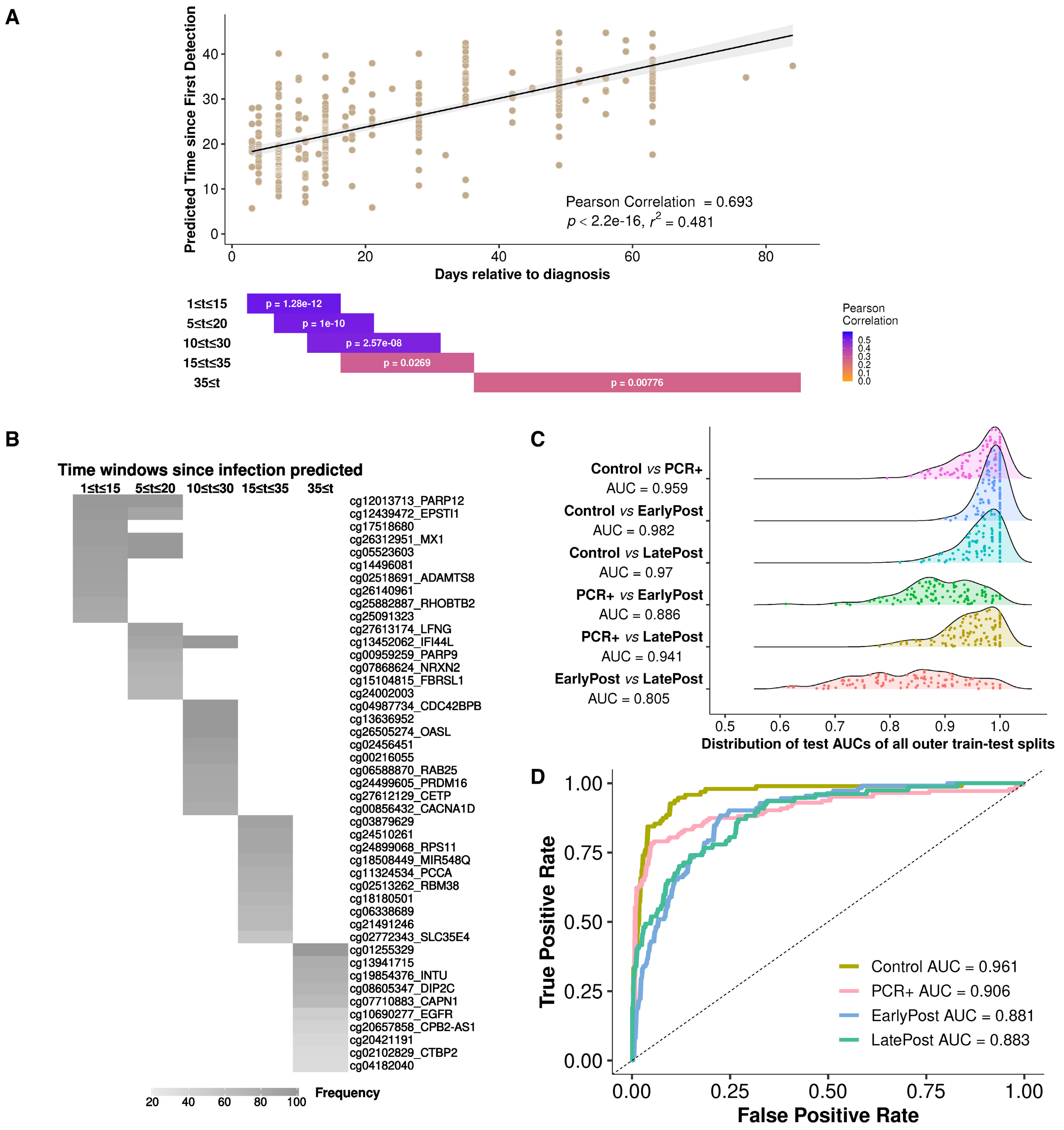

Figure 3. SARS-CoV-2 infection methylation clock.

A *Top*, Regression model predicting time since infection. *Bottom*, Correlation and significance of models restricted to shorter time windows. The results shown were trained with mean squared error but are depicted as a correlation plot to facilitate interpretation.

B Comparison of the 10 most frequently utilized sites when regression models are repeatedly generated for each time window.

C Accuracy of binary blood methylation classification models as the AUC, in distinguishing samples from preinfection, infection, and postinfection pseudotime periods.

D Accuracy of blood methylation multiclass classifier in classifying samples from time periods relative to infection.

 © 2023 The Authors

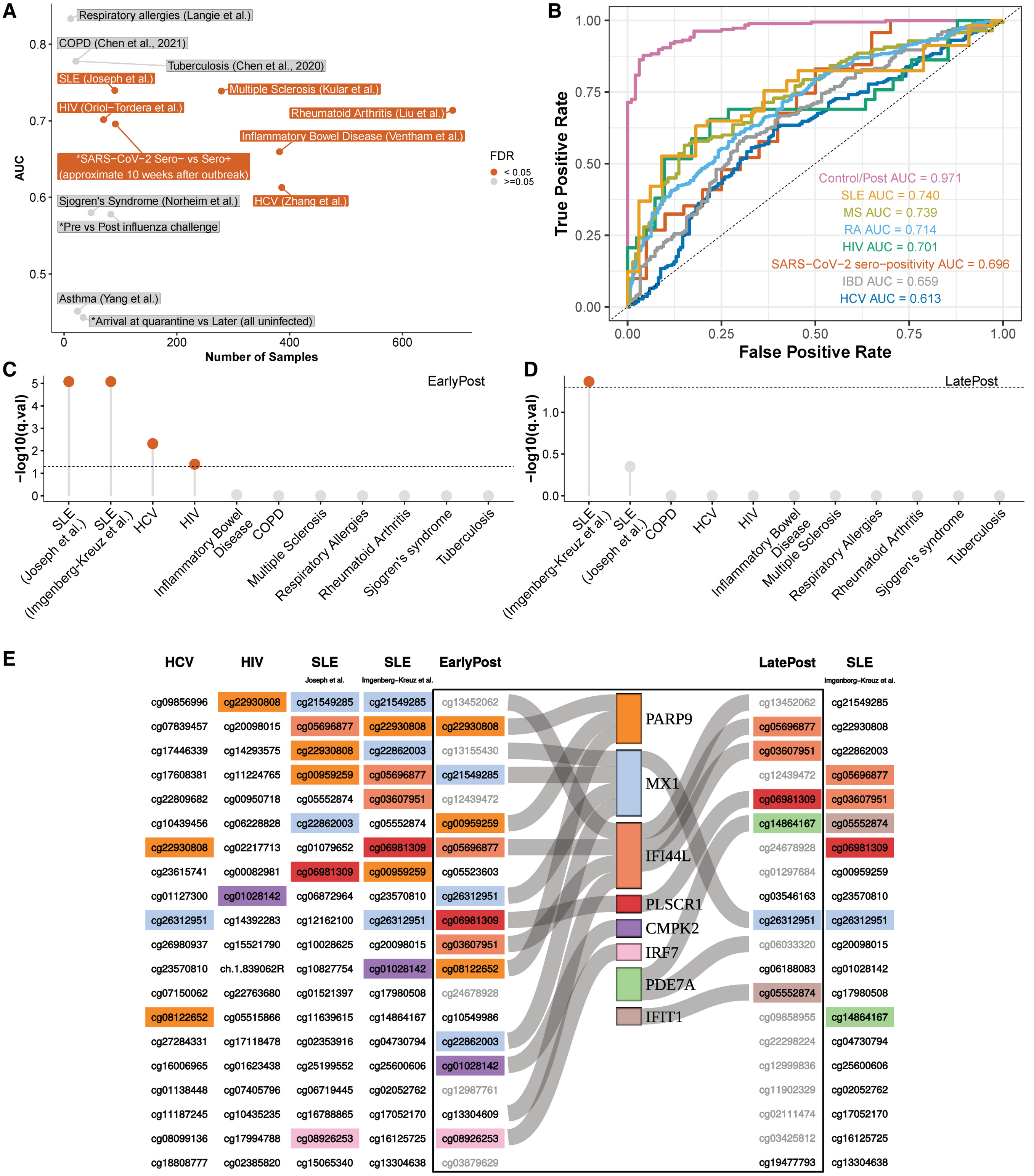
A
Respiratory allergies (Langie et al.)
COPD (Chen et al., 2021)
Tuberculosis (Chen et al., 2020)
SLE (Joseph et al.)
Multiple Sclerosis (Kular et al.)
HIV (Oriol−Tordera et al.)
Rheumatoid Arthritis (Liu et al.)
Inflammatory Bowel Disease (Ventham et al.)
*SARS−CoV−2 Sero− vs Sero+ (approximate 10 weeks after outbreak)
Sjogren's Syndrome (Norheim et al.)
HCV (Zhang et al.)
*Pre vs Post influenza challenge
Asthma (Yang et al.)
*Arrival at quarantine vs Later (all uninfected)
AUC
Number of Samples
FDR
< 0.05
>=0.05
B
True Positive Rate
False Positive Rate
Control/Post AUC = 0.971
SLE AUC = 0.740
MS AUC = 0.739
RA AUC = 0.714
HIV AUC = 0.701
SARS−CoV−2 sero−positivity AUC = 0.696
IBD AUC = 0.659
HCV AUC = 0.613
C
EarlyPost
−log10(q.val)
SLE (Joseph et al.)
SLE (Imgenberg-Kreuz et al.)
HCV
HIV
Inflammatory Bowel Disease
COPD
Multiple Sclerosis
Respiratory Allergies
Rheumatoid Arthritis
Sjogren's syndrome
Tuberculosis
D
LatePost
E
HCV
HIV
SLE Joseph et al.
SLE Imgenberg−Kreuz et al.
EarlyPost
LatePost
PARP9
MX1
IFI44L
PLSCR1
CMPK2
IRF7
PDE7A
IFIT1

Figure 4.

Figure 4. Post-SARS-CoV-2 infection methylation pattern comparison with other conditions.

A Performance of a binary classifier trained to distinguish postinfection (EarlyPost or LatePost) vs. controls in other datasets. * marks current study datasets. "SARS-CoV-2 Sero− vs. Sero+": retrospective study dataset of Marine recruits exposed during late March-early April 2020, assayed for blood DNA methylation in mid-July, and distinguished by SARS-CoV-2 serology status. "Arrival at Quarantine vs. Later": PCR-negative study participants upon arrival vs. later during training. See Materials and Methods for details.

B Receiver operator curve and significance of AUC for datasets showing FDR < 0.05 in panel (A).

C, D Enrichment of 20 most significantly hypomethylated DMS ranked by absolute delta beta values relative to top hypomethylated DMS in EarlyPost (C) or LatePost (D) vs. Control.

E Top-ranked hypomethylated DMS upon SARS-CoV-2 infection compared with other diseases showing enrichment in (C, D). Sites identified both in the SARS-CoV-2 study and at least one other condition are highlighted. Light gray sites were ranked in this study but not assayed in other studies. Gene annotations are indicated.

a phenomenon referred to as trained immunity (Netea *et al*, 2020). On a mechanistic level, trained immunity is attributed to a permissive epigenetic state that allows for faster upregulation of chemokines and receptors needed to mount an immune response. Trained immunity has been invoked to explain infection-induced protection in animals that lack an adaptive immune system and cross-pathogen protection. The longitudinal nature of our cohort combined with a well-defined postinfection methylation state enabled us to evaluate indirectly whether the postinfection methylation state we define is protective against infection (Fig 5A).

We reasoned that prior to infection, the methylation patterns in subsequently infected longitudinal study participants vary in their relative similarity to the methylation signatures post PCR positivity. In other words, the control samples could already be in a postinfection-like state, for example as a result of infection with a different infectious agent or another immune challenge such as vaccination. We note that the SARS-CoV-2 vaccine was not available at the time of this study. Thus, as a quantification of the similarity of preinfection control samples to the patterns seen following infection, we used the probability of these samples being misclassified to the active infection period (PCR^+), the early period following infection (EarlyPost), or the later period following infection by the multi-class classifier (Fig EV5A).

We next examined whether similarity to the postinfection methylation state at baseline was predictive of the future response to SARS-CoV-2 infection. Because symptoms were so sparse in this cohort, we used the minimum SARS-CoV-2 PCR cycle (negated to indicate viral load in arbitrary units) as a measure of the effectiveness of controling the virus infection. We examined the relationship of the preinfection sample misclassification probabilities to the subsequent level of the virus. We note that nearly all samples are, in fact, correctly classified by the model. We use the term "misclassification" here to reflect merely the quantitative probability obtained from the model of classifying the samples as belonging to the wrong class. Probabilities of these samples being misclassified as active infection or LatePost were not significantly associated with viral load (Fig EV5B). The probabilities of the preinfection samples being misclassified as EarlyPost were associated with having higher maximal levels of virus detected by PCR ($P = 0.001$, Spearman rank correlation; Fig 5B). This result demonstrates that baseline methylation values are indeed predictive of future infection response. An identical analysis using gene expression did not yield significant results (Appendix Fig S3), supporting a key role of the methylation-encoded epigenetic state.

However, while we demonstrate a clear predictive power for baseline methylation, the direction of association is the opposite to that found in trained immunity. If the postinfection-like state were protective, we would expect it to correlate with lower viral loads. Notably, we find the opposite result (Fig 5B). This result can be confirmed by looking at individual features that contribute to our EarlyPost model. Among the top 16 CpG sites used by the model, we highlight two hypomethylated sites in IFI44L, which were individually inversely correlated with virus level (Fig 5B). These results suggest that individuals having preinfection blood methylation patterns similar to that characteristic of the post-PCR-positive period showed a less effective suppression of SARS-CoV-2 during infection.

In order to evaluate the generalizability of these findings to a more diverse cohort, we applied our postinfection model to a SARS-CoV-2 infection dataset from a different cohort having a broader age range (50.6 ± 17.2), more balanced sex composition (70 female, 92 male) and that included severe outcomes (Konigsberg *et al*, 2021). We found that the postinfection probability calculated on methylation state early in the disease course was significantly associated with disease severity and death (Fig 5C), further supporting the hypothesis that the state we identify is associated with reduced effectiveness of the immune response to SARS-CoV-2 infection.

We also applied our postinfection model to an *in vivo* and *in vitro* methylation study of BCG vaccination, one of the best-characterized perturbations for inducing trained immunity (Bannister *et al*, 2022). We found that the similarity to the SARS-CoV-2 postinfection state was not significantly changed when comparing either the *in vivo* or the *in vitro* (Fig 5D) pre- and post-BCG infection samples, further supporting the view that the epigenetic state we identify is distinct from trained immunity.

While the mechanistic details need to be further elucidated, we can hypothesize about the reasons for these contradictory findings. Both our gene expression and methylation data are heavily dominated by interferon-related genes and loci. Many interferon-induced genes (ISGs) have well-characterized antiviral activity and provide protection on the cellular and organismal levels (McNab *et al*, 2015). However, a growing body of evidence suggests that interferon signaling provides important immunoregulatory functions (Lee & Ashkar, 2018), and the effects of interferons on infection susceptibility are complex and context-dependent (McNab *et al*, 2015). Indeed, in our study, some of the most persistent hypomethylated loci are located near IFI44L and FKBP5 (Fig 1E), two genes that have been shown to negatively regulate antiviral responses (DeDiego *et al*, 2019a, 2019b). Together, these observations suggest that the epigenetic memory we observe may in fact reflect an interferon regulatory feedback state that correlates with reduced capacity for viral suppression. If this were the case, we would expect that the probability of being in a postinfection-like state as defined by our model should increase with the number of infections and thus with age. We confirm this conjecture in several

 © 2023 The Authors

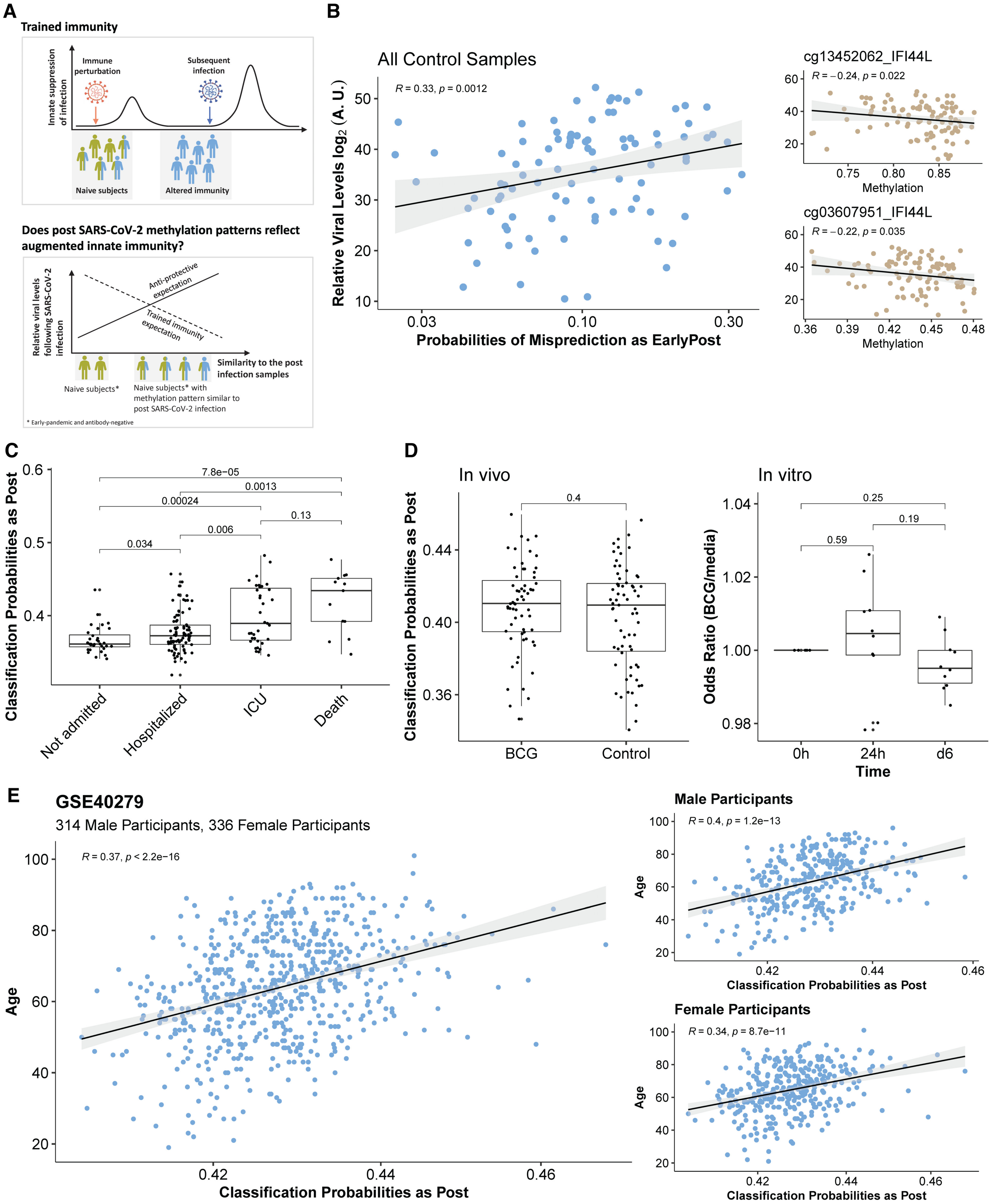
A
Trained immunity
Innate suppression of infection
Immune perturbation
Subsequent infection
Naive subjects
Altered immunity
Does post SARS-CoV-2 methylation patterns reflect augmented innate immunity?
Relative viral levels following SARS-CoV-2 infection
Anti-protective expectation
Trained immunity expectation
Similarity to the post infection samples
Naive subjects*
Naive subjects* with methylation pattern similar to post SARS-CoV-2 infection
* Early-pandemic and antibody-negative
B
All Control Samples
R = 0.33, p = 0.0012
Relative Viral Levels log2 (A. U.)
Probabilities of Misprediction as EarlyPost
cg13452062_IFI44L
R = −0.24, p = 0.022
Methylation
cg03607951_IFI44L
R = −0.22, p = 0.035
Methylation
C
Classification Probabilities as Post
7.8e-05
0.0013
0.00024
0.13
0.006
0.034
Not admitted
Hospitalized
ICU
Death
D
In vivo
In vitro
Classification Probabilities as Post
0.4
BCG
Control
Odds Ratio (BCG/media)
0.25
0.19
0.59
0h
24h
d6
Time
E
GSE40279
314 Male Participants, 336 Female Participants
R = 0.37, p < 2.2e−16
Age
Classification Probabilities as Post
Male Participants
R = 0.4, p = 1.2e−13
Female Participants
R = 0.34, p = 8.7e−11

Figure 5.

Figure 5. Persistent methylation state predicts future infection trajectories.

A Schematic illustration of the trained immunity phenomenon and expectations of possible protective and antiprotective effects of the post-SARS-CoV-2 methylation state.

B Correlation between maximum relative viral level during infection and the probabilities of misclassification as EarlyPost (*Left*) using the multiclassifier model (see Fig 3D); correlation of two hypomethylated IFI44L sites with viral load (*Right*). A.U., arbitrary units, calculated as 80-(minimum cycle threshold PCR result) for each participant. See Fig EV5B for plots of the correlation of misclassification probabilities for the other infection periods.

C Postinfection-like state is significantly associated with negative outcomes following SARS-CoV-2 infection in an older cohort with severe outcomes. As infection outcomes and postinfection probabilities (see panel E) are both associated with age, age was regressed out from the input methylation data for this analysis, showing these results are independent of subject age. The boxplot displays the 25th, 50th, and 75th percentiles, with whiskers that extend up to 1.5 times the interquartile range or the range of the data, whichever is smaller. *P*-values are from the Wilcoxon rank-sum test.

D There is no significant difference comparing samples following BCG vaccination of human subjects or BCG stimulation *in vitro* with respect to the model prediction probabilities as post-SARS-CoV-2 infection. The boxplot displays the 25th, 50th, and 75th percentiles, with whiskers that extend up to 1.5 times the interquartile range or the range of the data, whichever is smaller. *P*-values are from the Wilcoxon rank-sum test.

E Applying the multiclass classifier on a reference methylation cohort shows a strong positive correlation between age and prediction probabilities as Post. Results are comparable in males and females.

large cohorts of methylation data and find a similar relationship in both males and females (Fig 5E and Appendix Fig S4). Overall, our results support the formulation that the baseline methylation state, but not gene expression, is predictive of response to subsequent infection challenge. However, the state we identify following SARS-CoV-2 infection is antiprotective and represents an epigenetic phenomenon that is distinct from trained immunity.

Discussion

Our study provides a fine grain characterization of the temporal dynamics of methylation changes following an acute perturbation. Our results indicate that in immune-naive healthy young adults, asymptomatic and mild SARS-CoV-2 infections induced prolonged alterations of DNA methylation. The dynamics of these methylation changes observed during several months of follow-up were used to develop a methylation clock that accurately predicts time since infection. These results suggest that in addition to the lifetime methylation clocks that have been described, the methylome may also contain a record of the timing of individual acute environmental exposures.

These dynamic epigenetic processes may have important implications for health and disease. In the context of immunological stimuli, methylation and other induced epigenetic changes can provide faster induction of immune responses, thus benefiting host defense (Netea *et al*, 2020). We find that the postinfection methylation signature we define is related to other pro-inflammatory conditions such as chronic infections and autoimmune diseases, with the association being particularly strong for Systemic Lupus Erythematosus (SLE). Strikingly, we find that contrary to the trained immunity phenomenon, in this cohort the presence of an early postinfection-like methylation state prior to infection is antiprotective for the SARS-CoV-2 infection that occurred subsequent to these baseline measurements. This potentially deleterious effect of SARS-CoV-2 infection may be relatively short-lived as we found that the presence of a late postinfection-like methylation state prior to infection showed only a nonsignificant trend towards being antiprotective. The persistence of SARS-CoV-2-induced methylation changes and their functional effect, if any, beyond the several months duration of the present investigation requires additional study. An increased subsequent infection risk has also been observed following other primary infections, such as measles (Behrens *et al*, 2020). We further find that the presence early after SARS-CoV-2 infection of a methylation state that is similar to the post-SARS-CoV-2 infection methylation state defined by our model is associated with poorer outcomes in a more diverse cohort. We speculate that the state we define using our study is related to a regulatory feedback process that downregulates interferon activity and results in reduced viral suppression. Overall, our results suggest that the persistent SARS-CoV-2 methylation we identify represents a dysregulated epigenetic state.

Materials and Methods

Sources of samples for analysis

COVID-19 Health Action Response for Marines (CHARM) study

We obtained samples as part of the prospective COVID-19 Health Action Response for Marines (CHARM) study, which followed predominantly male, US Marine recruits after a 2-week home quarantine. A second supervised 2-week quarantine followed, which included SARS-CoV-2 mitigation measures such as mask-wearing and social distancing, along with daily temperature and symptom monitoring. At the time of arrival at quarantine, CHARM study participants were tested for SARS-CoV-2 infection via quantitative polymerase-chain-reaction (qPCR) assay of nasal swab specimen and evaluated for baseline SARS-CoV-2 IgG seropositivity, defined as a dilution of 1:150 or more on receptor-binding domain and full-length spike protein ELISA. SARS-CoV-2 infection and COVID-19-related symptoms or any other unspecified symptoms were assessed at weeks 1 and 2 of quarantine. Study participants included Marines who had three negative PCR tests during quarantine and a baseline serum serology test that indicated them as either seropositive or seronegative for SARS-CoV-2. As recruits went on to basic training at Marine Corps Recruit Depot-Parris Island SC, PCR tests were performed at weeks 2, 4, and 6 in both seropositive and seronegative groups. Additionally, a baseline neutralizing antibody titer was measured on all subsequently seropositive participants, and a follow-up symptom questionnaire was provided. We also collected PAXgene blood samples for RNA-seq analysis and EDTA blood samples for DNA methylation analysis from PBMCs. All samples were frozen at −80°C after collection prior to processing for RNA-seq and

© 2023 The Authors

methylation analysis. Additional details regarding CHARM study are described in Letizia *et al* (2021). Notably, researchers had access to de-identified PCR data.

Retrospective study of US marines

Marine recruits in training at Marine Corps Recruit Depot-Parris Island SC who were in companies exposed to SARS-CoV-2 during a cluster occurring from Mid-March to Mid-April 2020 were later enrolled in a retrospective blood sampling study. Only a few study participants had been tested for SARS-CoV-2 at the time of the cluster. Samples were obtained approximately 6 and 10 weeks after exposure, with the 10-week samples analyzed for the present study. EDTA blood samples were used for DNA methylation analysis from PBMCs. Additional details regarding this study and the serological analysis of these samples are described in Ramos *et al* (2021). Notably, mild symptoms included runny nose, sore throat, cough, subjective fever, headache, chills, and nausea (see table 1 in Ramos *et al*, 2021).

Influenza challenge study

Samples were analyzed from the placebo vaccination group from an influenza H3N2 (A/Belgium/2417/2015) virus human challenge model study. DNA methylation analysis was performed using cryo-preserved PBMC collected from 41 participants before the challenge and 28 days after the challenge for each subject. Additional study details can be found at trial NCT03883113 at clinicaltrials.gov.

Protection of human subjects

Institutional Review Board approval was obtained from the Naval Medical Research Center (protocol number NMRC.2020.0006) in compliance with all applicable US federal regulations governing the protection of human subjects. All participants provided written informed consent, and the experiments conformed to the principles set out in the WMA Declaration of Helsinki and the Department of Health and Human Services Belmont Report.

Data production

RNA isolation and cDNA library preparation

Total RNA from PAXgene preserved blood was extracted using the Agencourt RNAdvance Blood Kit (Beckman Coulter, Indianapolis, IN) on a BioMek FX^{P} Laboratory Automation Workstation (Beckman Coulter). Concentration and integrity (RIN) of isolated RNA were determined using the Quant-iT™ RiboGreen™ RNA Assay Kit (Thermo Fisher) and an RNA Standard Sensitivity Kit (DNF-471, Agilent Technologies, Santa Clara, CA, USA) on a Fragment Analyzer Automated CE system (Agilent Technologies), respectively. Subsequently, cDNA libraries were constructed from total RNA using the Universal Plus mRNA-Seq kit (Tecan Genomics, San Carlos, CA, USA) in a Biomek i7 Automated Workstation (Beckman Coulter). Briefly, mRNA was isolated from purified 300 ng total RNA using oligo-dT beads and used to synthesize cDNA following the manufacturer's instructions. The transcripts for ribosomal RNA (rRNA) and globin were further depleted using the AnyDeplete kit (Tecan Genomics) prior to the amplification of libraries. Library concentration was assessed fluorometrically using the Qubit dsDNA HS Kit (Thermo Fisher), and quality was assessed with the HS NGS Fragment Kit (1–6,000 bp; DNF-474, Agilent Technologies).

RNA sequencing and preprocessing of the RNA-seq data

Following library preparation, samples were pooled and preliminary sequencing of cDNA libraries (average read depth of 90,000 reads) was performed using a MiSeq system (Illumina), to confirm library quality and concentration. Deep sequencing was subsequently performed using an S4 flow cell in a NovaSeq sequencing system (Illumina; average read depth ~ 30 million pairs of 2 × 100 bp reads) at New York Genome Center.

Methylation data

All samples were frozen at −80°C after collection prior to processing for methylation analyses. Genomic DNA was extracted from cryo-preserved PBMC or blood collected in EDTA tubes using Genfind V3 (Beckman Coulter) on a BioMek FX^{P} Laboratory Automation Workstation (Beckman Coulter). All DNA samples were quantified using both absorbance (NanoDrop 2000; Thermo Fisher Scientific, Waltham, MA) and fluorescence-based methods (Qubit; Thermo Fisher Scientific) using standard dyes selective for double-stranded DNA, minimizing the effects of contaminants that affect the quantitation.

DNA methylation was quantified using Illumina Infinium Human Methylation EPIC Bead Chip array (Illumina Inc., San Diego, CA) according to the manufacturer's instructions at the University of Minnesota Genomic Center. Briefly, 500 ng of DNA from each sample was treated with sodium bisulfite, using the EZ-96 DNA Methylation-Gold kit (Zymo Research, CA, USA). The bisulfite-converted amplified DNA products were denatured into single strands and hybridized to the Illumina Infinium Human Methylation EPIC Bead Chip array (Illumina Inc.). The hybridized BeadChips were stained, washed, and scanned for the intensities of the unmethylated and methylated bead types using Illumina's iScan System. The DNA methylation beta values were obtained from the raw IDAT files by using the ChAMP package in R. Samples from the same individual were processed together across all experimental stages to negate any methodological batch effects.

Data processing and quality assessment

RNA-seq

The RNA-seq reads were converted from raw RSEM counts to the final gene-level quantification following the pipeline in Fig EV4A. We only included protein-coding genes and filtered out low-expressed genes based on the mean expression levels. Overall, we had 11,436 genes left after filtering.

Methylation

We adopted the ChAMP pipeline (Tian *et al*, 2017) to process the raw (IDAT) files from Illumina Methylation microarray platform. The normalization steps and probe filtering criterion are illustrated in Fig EV4B. We applied ComBat (Johnson *et al*, 2007) in the M-value space to regress out potential technical covariates including Array (EPIC array), Slide (EPIC array), and batches (EPIC array plates). Then, we converted methylation levels of 707,361 CpG sites from M-values to beta values for all downstream differential methylation analysis and modeling. The regression of cell-type proportion to remove the confounding effect used for clustering was performed in both beta value and M-value space, with the results obtained in M-value space shown in Fig 2 (see Materials and Methods, Subsection Temporal clustering).

For both RNA-seq and methylation samples, only samples from subjects who were PCR- and serology-negative when enrolled in the study were kept for the downstream analysis (Fig EV1). We further filtered out samples if they were outliers in the principal component (PC) space. We calculated the Mahalanobis distances to the center in the PC space of the first five principal components correspondingly. As the distances follow a chi-square distribution, samples with significant *P*-values (0.01 divided by the number of samples included in the test) were classified as outliers. In total, there were two methylation samples, and three RNA-seq samples excluded from downstream analysis.

Computational inference of cell-type proportions

Methylation

We estimated the proportions of six major cell types (B cells, Granulocytes, Monocytes, NK cells, CD4 T cells, and CD8 T cells) using a standard reference-based method (Houseman *et al*, 2012). We took the original CellType450K basis matrix and replaced the values with those from (Roy *et al*, 2021; Illumina Methylation microarray). This was done to help remove bias induced by the platform inconsistency. We compared cell-type specificity obtained with the updated basis matrix to that obtained using the standard Houseman *et al* (2012) basis. We found that the cell-type specificity blocks were preserved and in some cases actually improved in the updated matrix. In particular, we find that the hypomethylated values are generally lower in the new basis (Appendix Fig S9A and B). The overall correlation of the standard basis values against the updated basis values is nearly perfect (Appendix Fig S9C).

The differential methylation site analysis was performed on raw beta values using these cell-type proportions as covariates (see Materials and Methods, Sub-section Differential gene and methylation site analysis). For clustering analysis, we created a cell-type-corrected matrix by regressing out cell-type proportions first (see our elaboration in Sub-section Temporal clustering). The machine learning models used the raw beta value matrix (see Subsection Machine learning models).

RNA-seq

Our key goal for proportion inference was to ascertain whether the major trends in our data such as more prolonged alterations in DNA versus RNA were insensitive to cell proportion correction. As proportion estimation from RNA and methylation differs greatly in terms of robustness and the number of cell types that can be estimated (methylation is more robust while RNA can be used to estimate some rare cell types) in order to formulate a fair comparison we correct both modalities for the same cell proportion estimates. We used the methylation estimated proportions as a gold standard. For RNA samples with no matching methylation, the proportions were imputed using a simple machine learning model. We used genes included in Cibersort LM22 (Newman *et al*, 2015) to train an elastic net model (Friedman *et al*, 2010; $\alpha = 0.9$, 10-fold CV) to predict the inferred cell-type proportions based on paired methylation data. Then, we selected lambda corresponding to the minimum cross-validation error to generate predictions for the complete RNA-seq data. Similarly, we regressed out inferred cell-type proportions by linear regression from the uncorrected gene expression profiles. The gene expression profiles that were corrected for cell-type proportions would be used for some downstream analysis. We find that using alternative methods of proportion estimation including a newly published methylation basis with 12 cell types (Salas *et al*, 2022) and CIBERSORTx (Newman *et al*, 2019) did not alter the main conclusions. We produce alternative versions of Fig 1B, which shows the timing of methylation and RNA changes, using different proportion estimation methods and find that the overall trend is unchanged (Appendix Fig S5). We also visualize cell proportion differences across time points in Appendix Fig S6.

Differential gene and methylation site analysis

We adopted limma (Ritchie *et al*, 2015) to perform differential analysis for both methylation data and RNA-seq data. We noted that many methylation probes with similar time trajectory patterns had highly variable value ranges. To account for this, we transformed the beta values into z-scores. Subsequent methylation analysis was performed using limma in this standardized space. Because the standardization is a linear transformation, it does not affect the significance of the limma linear model coefficients. The differential output from the limma analysis is referred to as log fold change for the RNA data and as normalized delta beta for the methylation data. We included age and sex as biological covariates in the limma models when cell-type proportions were not corrected. When cell-type proportions were corrected, the proportions of six major cell types (Monocyte%, Bcell%, Gran%, CD4T%, CD8T%, NK%) were also included as biological covariates. The raw *P*-values were corrected by the Benjamini–Hochberg (BH) method, and the significance cutoff of FDR < 0.05 was applied.

Comparison of methylation after symptomatic and asymptomatic infections

The participant symptom category (symptomatic, asymptomatic) was determined by the result of temperature screening and a 14-symptom questionnaire obtained concerning the week prior to each study visit. For details, see Letizia *et al* (2021). Responses covering up to 2 weeks before and after the initial PCR-positive test were used for group assignment. We performed differential analysis comparing these symptomatic and asymptomatic participants separately for each time period (Control, First, Mid, EarlyPost, and LatePost; see Datasets EV5 and EV6).

Temporal clustering

We clustered CpG sites, which were aligned to the first PCR-positive day for each subject (Fig 2A). We only included time points with more than four associated samples, giving 20 time points. The beta value matrix was first corrected for cell-type proportions (we investigated different methods for performing this step, as detailed below). After correction, we fitted a loess (local polynomial regression fitting) curve for each CpG site, then we discretized the fitted curve and only kept the values corresponding to the 20 unique time points.

We clustered CpG sites with respect to these discrete time series, and we evaluated the similarity of each pair of time series using dynamic time-warping distance (Leodolter *et al*, 2021). Dynamic time-warping is an algorithm that calculates the optimal matching between two time series (Liu & Muller, 2003; Leng & Muller, 2006).

© 2023 The Authors

It measures similarity based on overall trajectory, regardless of speed. These characteristics make it beneficial for clustering differential features according to their temporal trajectory patterns. The warping window size was set to be 20. The distance matrix was squared and then used as input for the hierarchical clustering step (Ward's minimum variance method, seven clusters). In summary, our temporal clustering analysis includes four consecutive steps: (i) Correct for cell-type proportions, (ii) Smooth the normalized data by local polynomial regression fitting, (iii) Calculate the dynamic time-warping distance matrix, and (iv) Run hierarchical clustering using the distance matrix as input.

We investigated two different approaches to correct for the cell-type proportions: The first approach named B2M2B is to first convert the beta value matrix to M-value matrix, regress out cell-type proportions in the M-value space by linear regression, and convert the M-value matrix back to the beta value space. We also considered an alternative approach where cell-type proportions are directly regressed out in the beta value space, and we name this approach B_regress (see Appendix Fig S7A). We chose between these two normalization strategies (B2M2B vs. B_regress) by running through the same pipeline detailed above with all hyperparameters fixed in steps (2–4) and comparing all the intermediate outputs side by side. First, B2M2B and B_regression generated nearly identical beta value matrices after correcting for cell-type proportions (see Appendix Fig S7B). Next, the corresponding dynamic warping distance matrices were also highly correlated (see Appendix Fig S7C). Finally, we compared the cluster assignments after running through the hierarchical clustering step. Due to the NP-hard nature of the hierarchical clustering problem, Ward's minimum variance method tried to minimize the total within-cluster variances (SSE) in a heuristic manner in practice, and the different initializations might end up with different local optimal solutions. B_regress resulted in a larger total within-cluster variance (SSE) (see Appendix Fig S7A), indicating that the corresponding cluster assignment was indeed less tight compared with that based on B2M2B. From the perspective of the clustering optimization problem, the B2M2B cluster assignment is a better solution. We also investigated the biological coherence of the resulting clusters using the downstream enrichment pipeline (see Materials and Methods, Sub-section Enrichment analysis by temporal cluster). We found that the B2M2B cluster assignment was also more biologically coherent, as the corresponding transcription factor (TF) enrichment results identified unique enriched TFs for all seven clusters, whereas the B_regress analysis failed to identify unique TFs that were significantly enriched with Cluster 2, 6 and 7 (see Fig 2 and Appendix Fig S8). We show the clustering analysis and annotations based on the B2M2B method in Fig 2 and the parallel analysis using B_regress in Appendix Fig S8.

Enrichment analysis by temporal cluster

These enrichment analyses comparing each cluster with the other clusters with respect to both discrete phenotypes, continuous phenotypes, and transcription factor binding sites are presented in Figs 2 and EV3.

Pathway/cell markers and discrete phenotype enrichment analysis

We first mapped DMS to associated genes based on Illumina Methylation microarray annotation. If multiple DMS were mapped to the same gene, the corresponding gene would be only included as foreground or background once. We combined canonical pathways and hallmark pathways from MsigDB (v7.4) (Liberzon *et al*, 2011, 2015) together to formulate a comprehensive pathway set. The other discrete phenotypes included cell markers (scRNA-seq; Stuart *et al*, 2019), gene region feature categories, and CpG island categories. We adopted the hypergeometric test by cluster to conduct enrichment analysis.

Continuous phenotype enrichment analysis

For each DMS, we collected four different categories of continuous phenotypes. The first category was the Blueprint Epigenome project cell-type signatures (Stunnenberg *et al*, 2016). We downloaded the bigWig file matching "CPG_methylation_calls.bs_call.GRCh38" from Blueprint. Beta values corresponding to EPIC array probes were extracted using bwtool (Pohl & Beato, 2014). Missing values were imputed using *knn.impute* and the replicates were mean summarized. CpG levels were z-scored to define relative cell-type specificity. We calculated the spearman rank correlations between one hot encoding of the cluster membership of all DMS and the corresponding normalized Blueprint CpG levels to test for significant associations. The second category was the correlation with ref-based cell-type proportions. This was defined as the Pearson correlations of DMS methylation levels and the inferred proportions of six major cell types (B cells, Granulocytes, Monocytes, NK cells, CD4 T cells, and CD8 T cells). The third class was the CG pattern/GC pattern/GC ratio. The CG pattern was defined as the number of CpG (dinucleotides) divided by N-1 (number of dinucleotide positions), and the GC pattern was defined as the number of GpC divided by the number of dinucleotide positions. GC ratio was the ratio of G/C mono-nucleotides. The last class was the distance of each DMS to the closest transcription start sites (TSS). We ranked DMS based on each class of the continuous phenotypes and conducted the Wilcoxon rank-sum test for enrichment analysis.

Transcription factor enrichment analysis

We utilized Homer (v4.11; Heinz *et al*, 2010) to test the enrichment of transcription factor binding sites by cluster within a 200 bp window centered at each DMS. The transcription factors included in the analysis were the 440 known motifs for vertebrates included in Homer. When the 200 bp windows of one cluster are specified as the foreground sequences, the 200 bp windows of other clusters were used as the background.

Enrichment analysis of reported differential CpG sites

In Fig 4C and D, we tested whether reported differentially methylated CpG sites of other diseases were enriched with respect to the rankings in the longitudinal study. For many published studies, we found that *de novo* analysis of the raw data did not replicate the DMS rank lists reported by the authors. We reasoned that the discrepancies most likely resulted from the selection of covariates, and because the original authors had privileged knowledge about covariates that may improve the analysis, we used the published DMS calls from each study for our comparative analysis. Accordingly, we extracted the DMS from each published manuscript and ordered them based on the absolute delta beta values. Then, we took the top 20 hypomethylated sites and tested whether they were enriched

© 2023 The Authors

given the rankings (ordered by absolute delta beta values) of significantly hypomethylated sites (EarlyPost vs. Control or LatePost vs. Control) from our analysis of the longitudinal CHARM study data using the Wilcoxon rank-sum test.

Machine learning models

Overview of model construction

We utilized a nested cross-validation (Fig EV4C; Simon *et al*, 2003; Teschendorff, 2019) strategy to build different prediction models for the longitudinal study. There are two loops in the nested cross-validation procedure where an "inner" cross-validation step is nested inside an "outer" train-test split. The nested cross-validation strategy eliminates the possibility of selection bias when constructing the test-train split and more accurately estimates the generalization error of the model.

Unless otherwise specified, there were 100 outer train-test splits. We used the elastic net model for both regression and classification tasks as the inner cross-validation model. The input was the raw beta value matrix or gene expression profile without correcting for the cell-type proportions. The average predictions reported in the manuscript (Figs 3 and EV5B, and Appendix Fig S3, and Fig 5A) were calculated in two steps. First, the test predictions (classification probabilities or values of response variables) were averaged for each sample using outer train-test splits that include this sample in the test set. Then, we took the average predictions of all samples to evaluate the AUC (classification) or the correlation value (regression) with respect to the ground truth. These metrics were referred to as the average AUC and the average correlation. In order to build a general model that is applicable to external datasets, we first selected features that were robust (frequently selected over all outer train-test splits) and then built the model only with these most stable features. The selection of the most stable features also limits the number of features that will be missing when applying the model trained on our 850 K EPIC data to the 450 K platform data available in nearly all public datasets studied.

Binary classification

We constructed a binary classification model for each pair out of four defined groups (Figs 3C and 4A and B): Control, PCR^+ (combining First and Mid together), EarlyPost, and LatePost (Fig 3C). All 707,361 CpGs were included as features without preselection. 10% of the available data were used as the test set for each outer train-test split, and we utilized the elastic net model (*glmnet (family = "binomial")*) for the inner cross-validation step ($\alpha = 0.9$, 5-fold cross-validation).

We also built a binary classification model distinguishing Control samples with Post samples (including both EarlyPost and LatePost samples). All 707,361 CpGs were included as features without preselection. After the nested cross-validation step, we selected features that were most frequently utilized across outer iterations (> 90% of all outer train-test splits, shown in Dataset EV11) to build the model for unseen data.

Features were transformed into z-scores to build an elastic net model ($\alpha = 0.9$, 5-fold cross-validation). Features were also first standardized before applying this pretrained model to other datasets (Fig 4A and B). If the dataset was based on the HM450K microarray, we imputed the CpG sites that are not available on the HM450K microarray by all-zero vectors. We utilized the Wilcoxon rank-sum test to estimate the significance of AUCs and we calculated the adjusted *P*-values following the Benjamini–Hochberg correction.

Multiclass classification

We built a multiclass classification model with 10% of the available data as the test set for each outer train-test split (Figs 3D and 5A). All 707,361 CpGs were included as features without preselection, and we utilized the elastic net model (*glmnet(family = "multinomial")*) for the inner cross-validation step ($\alpha = 0.9$, 5-fold cross-validation).

Regression

We built a regression model using 10% of the available data as the test set for each outer train-test split (Fig 3A). All 707,361 CpGs were included as features without preselection, and we utilized the elastic net model (*glmnet(family = "gaussian")*) for the inner cross-validation step ($\alpha = 0.5$, 5-fold cross-validation). We repeatedly construct the regression model for each time window following the same steps above.

Methylation-gene annotation

The CpG-gene assignment is based on Illumina Methylation microarray annotation (manufacturer's manifest) for Genome assembly GRCh37 (hg19). The manifest also includes information on gene region feature categories and CpG island annotations. In our analysis, we categorized gene region feature categories into two main groups: promoter sites (including TSS1500, TSS200, 1st Exon, and 5' UTR) and gene body sites (including 3' UTR, Body, and ExonBnd annotations). The definition of these gene region feature categories can be found in (Illumina, 2014).

Data availability

All data needed to evaluate the conclusions in the paper are present in the paper and/or the Supporting Information. The datasets produced in this study are available in the following databases:

- RNA-seq data: Gene Expression Omnibus GSE198449 (https://www.ncbi.nlm.nih.gov/geo/query/acc.cgi?acc=GSE198449)
- Methylation data: Gene Expression Omnibus GSE219037 (https://www.ncbi.nlm.nih.gov/geo/query/acc.cgi?acc=GSE219037)

Expanded View for this article is available online.

Acknowledgements

We thank the New York Genome Center for performing the NovaSeq sequencing assays and the University of Minnesota Genomics Center for processing Illumina EPIC methylation array and data collection. This work was supported by a grant (9700130) from the Defense Health Agency through the Naval Medical Research Center and by the Defense Advanced Research Projects Agency (contract number N6600119C4022). This research was supported in part by the University of Pittsburgh Center for Research Computing, RRID:SCR_022735, through the resources provided. Specifically, this work used the HTC cluster, which is supported by NIH award number S10OD028483. SG was partially supported by NIH grants and contracts CA224319, DK124165, 75N91020R00055, CA263705, and CA196521.

© 2023 The Authors

Author contributions

Weiguang Mao: Formal analysis; visualization; methodology; writing – review and editing. **Clare M Miller:** Formal analysis; writing – review and editing. **Venugopalan D Nair:** Supervision; visualization; writing – review and editing. **Yongchao Ge:** Formal analysis; writing – review and editing. **Mary Anne S Amper:** Performing omics assays. **Antonio Cappuccio:** Formal analysis. **Mary-Catherine George:** Project administration. **Carl W Goforth:** Supervision. **Kristy Guevara**: Performing omics assays. **Nada Marjanovic**: Performing omics assays. **German Nudelman:** Formal analysis. **Hanna Pincas:** Writing – review and editing. **Irene Ramos:** Formal analysis. **Rachel S G Sealfon:** Writing – review and editing. **Alessandra Soares-Schanoski:** Formal analysis. **Sindhu Vangeti:** Formal analysis. **Mital Vasoya**: Performing omics assays. **Dawn L Weir:** Supervision. **Elena Zaslavsky:** Formal analysis. **Biobank Team:** Resources. **Seunghee Kim-Schulze:** Resources. **Sacha Gnjatic:** Resources. **Miriam Merad:** Resources. **Andrew G Letizia:** Supervision; investigation. **Olga G Troyanskaya:** Formal analysis. **Stuart C Sealfon:** Formal analysis; supervision; investigation; writing – original draft; writing – review and editing. **Maria Chikina:** Formal analysis; supervision; investigation; writing – review and editing.

Disclosure and competing interests statement

SG reports past consultancy or advisory roles for Merck and OncoMed; research funding from Boehringer Ingelheim, Bristol Myers Squibb, Celgene, Genentech, Janssen R&D, Pfizer, Regeneron Pharmaceuticals, and Takeda. SCS is a founder of GNOMX, Corp. Other authors declare that they have no conflict of interest. The views expressed in this article are those of the authors and do not necessarily reflect the official policy or position of the Department of the Navy, Department of Defense, nor the US Government. AGL, CWG, and DLW are a military service member or employee of the US Government. This work was prepared as part of their official duties. Title 17, U.S.C., §105 provides that copyright protection under this title is not available for any work of the US Government. Title 17, U.S.C., §101 defines a US Government work as a work prepared by a military service member or employee of the US Government as part of that person's official duties.

References

Balnis J, Madrid A, Hogan KJ, Drake LA, Chieng HC, Tiwari A, Vincent CE, Chopra A, Vincent PA, Robek MD *et al* (2021) Blood DNA methylation and COVID-19 outcomes. *Clin Epigenetics* 13: 118

Bannister S, Kim B, Dominguez-Andres J, Kilic G, Ansell BRE, Neeland MR, Moorlag S, Matzaraki V, Vlahos A, Shepherd R *et al* (2022) Neonatal BCG vaccination is associated with a long-term DNA methylation signature in circulating monocytes. *Sci Adv* 8: eabn4002

Behrens L, Cherry JD, Heininger U, Swiss Measles Immune Amnesia Study Group (2020) The susceptibility to other infectious diseases following measles during a three year observation period in Switzerland. *Pediatr Infect Dis J* 39: 478 – 482

Castro de Moura M, Davalos V, Planas-Serra L, Alvarez-Errico D, Arribas C, Ruiz M, Aguilera-Albesa S, Troya J, Valencia-Ramos J, Velez-Santamaria V *et al* (2021) Epigenome-wide association study of COVID-19 severity with respiratory failure. *EBioMedicine* 66: 103339

Chen R, Xia L, Tu K, Duan M, Kukurba K, Li-Pook-Than J, Xie D, Snyder M (2018) Longitudinal personal DNA methylome dynamics in a human with a chronic condition. *Nat Med* 24: 1930 – 1939

Corley MJ, Pang APS, Dody K, Mudd PA, Patterson BK, Seethamraju H, Bram Y, Peluso MJ, Torres L, Iyer NS *et al* (2021) Genome-wide DNA methylation profiling of peripheral blood reveals an epigenetic signature associated with severe COVID-19. *J Leukoc Biol* 110: 21 – 26

DeDiego ML, Martinez-Sobrido L, Topham DJ (2019a) Novel functions of IFI44L as a feedback regulator of host antiviral responses. *J Virol* 93: e01159-19

DeDiego ML, Nogales A, Martinez-Sobrido L, Topham DJ (2019b) Interferon-induced protein 44 interacts with cellular FK506-binding protein 5, negatively regulates host antiviral responses, and supports virus replication. *mBio* 10: e01839-19

Duttke SH, Chang MW, Heinz S, Benner C (2019) Identification and dynamic quantification of regulatory elements using total RNA. *Genome Res* 29: 1836 – 1846

Friedman J, Hastie T, Tibshirani R (2010) Regularization paths for generalized linear models via coordinate descent. *J Stat Softw* 33: 1 – 22

Furukawa R, Hachiya T, Ohmomo H, Shiwa Y, Ono K, Suzuki S, Satoh M, Hitomi J, Sobue K, Shimizu A (2016) Intraindividual dynamics of transcriptome and genome-wide stability of DNA methylation. *Sci Rep* 6: 26424

Heinz S, Benner C, Spann N, Bertolino E, Lin YC, Laslo P, Cheng JX, Murre C, Singh H, Glass CK (2010) Simple combinations of lineage-determining transcription factors prime cis-regulatory elements required for macrophage and B cell identities. *Mol Cell* 38: 576 – 589

Horvath S, Raj K (2018) DNA methylation-based biomarkers and the epigenetic clock theory of ageing. *Nat Rev Genet* 19: 371 – 384

Houseman EA, Accomando WP, Koestler DC, Christensen BC, Marsit CJ, Nelson HH, Wiencke JK, Kelsey KT (2012) DNA methylation arrays as surrogate measures of cell mixture distribution. *BMC Bioinformatics* 13: 86

Johnson WE, Li C, Rabinovic A (2007) Adjusting batch effects in microarray expression data using empirical Bayes methods. *Biostatistics* 8: 118 – 127

Konigsberg IR, Barnes B, Campbell M, Davidson E, Zhen Y, Pallisard O, Boorgula MP, Cox C, Nandy D, Seal S *et al* (2021) Host methylation predicts SARS-CoV-2 infection and clinical outcome. *Commun Med (Lond)* 1: 42

Lee AJ, Ashkar AA (2018) The dual nature of type I and type II interferons. *Front Immunol* 9: 2061

Leng X, Muller HG (2006) Classification using functional data analysis for temporal gene expression data. *Bioinformatics* 22: 68 – 76

Leodolter M, Plant C, Brändle N (2021) IncDTW: an R package for incremental calculation of dynamic time warping. *J Stat Softw* 99: 1 – 23

Letizia AG, Ge Y, Vangeti S, Goforth C, Weir DL, Kuzmina NA, Balinsky CA, Chen HW, Ewing D, Soares-Schanoski A *et al* (2021) SARS-CoV-2 seropositivity and subsequent infection risk in healthy young adults: a prospective cohort study. *Lancet Respir Med* 9: 712 – 720

Liberzon A, Subramanian A, Pinchback R, Thorvaldsdottir H, Tamayo P, Mesirov JP (2011) Molecular signatures database (MSigDB) 3.0. *Bioinformatics* 27: 1739 – 1740

Liberzon A, Birger C, Thorvaldsdottir H, Ghandi M, Mesirov JP, Tamayo P (2015) The molecular signatures database (MSigDB) hallmark gene set collection. *Cell Syst* 1: 417 – 425

Liu X, Muller HG (2003) Modes and clustering for time-warped gene expression profile data. *Bioinformatics* 19: 1937 – 1944

Liu J, Li S, Liu J, Liang B, Wang X, Wang H, Li W, Tong Q, Yi J, Zhao L *et al* (2020) Longitudinal characteristics of lymphocyte responses and cytokine profiles in the peripheral blood of SARS-CoV-2 infected patients. *EBioMedicine* 55: 102763

Lu AT, Quach A, Wilson JG, Reiner AP, Aviv A, Raj K, Hou L, Baccarelli AA, Li Y, Stewart JD *et al* (2019) DNA methylation GrimAge strongly predicts lifespan and healthspan. *Aging (Albany NY)* 11: 303 – 327

© 2023 The Authors

McNab F, Mayer-Barber K, Sher A, Wack A, O'Garra A (2015) Type I interferons in infectious disease. *Nat Rev Immunol* 15: 87 – 103

Netea MG, Dominguez-Andres J, Barreiro LB, Chavakis T, Divangahi M, Fuchs E, Joosten LAB, van der Meer JWM, Mhlanga MM, Mulder WJM *et al* (2020) Defining trained immunity and its role in health and disease. *Nat Rev Immunol* 20: 375 – 388

Newman AM, Liu CL, Green MR, Gentles AJ, Feng W, Xu Y, Hoang CD, Diehn M, Alizadeh AA (2015) Robust enumeration of cell subsets from tissue expression profiles. *Nat Methods* 12: 453 – 457

Newman AM, Steen CB, Liu CL, Gentles AJ, Chaudhuri AA, Scherer F, Khodadoust MS, Esfahani MS, Luca BA, Steiner D *et al* (2019) Determining cell type abundance and expression from bulk tissues with digital cytometry. *Nat Biotechnol* 37: 773 – 782

Pohl A, Beato M (2014) Bwtool: a tool for bigWig files. *Bioinformatics* 30: 1618 – 1619

Ramos I, Goforth C, Soares-Schanoski A, Weir DL, Samuels EC, Phogat S, Meyer M, Huang K, Pietzsch CA, Ge Y *et al* (2021) Antibody responses to SARS-CoV-2 following an outbreak among marine recruits with asymptomatic or mild infection. *Front Immunol* 12: 681586

Ritchie ME, Phipson B, Wu D, Hu Y, Law CW, Shi W, Smyth GK (2015) Limma powers differential expression analyses for RNA-sequencing and microarray studies. *Nucleic Acids Res* 43: e47

Ronnblom L, Leonard D (2019) Interferon pathway in SLE: one key to unlocking the mystery of the disease. *Lupus Sci Med* 6: e000270

Roy R, Ramamoorthy S, Shapiro BD, Kaileh M, Hernandez D, Sarantopoulou D, Arepalli S, Boller S, Singh A, Bektas A *et al* (2021) DNA methylation signatures reveal that distinct combinations of transcription factors specify human immune cell epigenetic identity. *Immunity* 54: 2465 – 2480

Sah P, Fitzpatrick MC, Zimmer CF, Abdollahi E, Juden-Kelly L, Moghadas SM, Singer BH, Galvani AP (2021) Asymptomatic SARS-CoV-2 infection: a systematic review and meta-analysis. *Proc Natl Acad Sci USA* 118: e2109229118

Salas LA, Zhang Z, Koestler DC, Butler RA, Hansen HM, Molinaro AM, Wiencke JK, Kelsey KT, Christensen BC (2022) Enhanced cell deconvolution of peripheral blood using DNA methylation for high-resolution immune profiling. *Nat Commun* 13: 761

Simon R, Radmacher MD, Dobbin K, McShane LM (2003) Pitfalls in the use of DNA microarray data for diagnostic and prognostic classification. *J Natl Cancer Inst* 95: 14 – 18

Stuart T, Butler A, Hoffman P, Hafemeister C, Papalexi E, Mauck WM III, Hao Y, Stoeckius M, Smibert P, Satija R (2019) Comprehensive integration of single-cell data. *Cell* 177: 1888 – 1902

Stunnenberg HG, International Human Epigenome Consortium, Hirst M (2016) The international human epigenome consortium: a blueprint for scientific collaboration and discovery. *Cell* 167: 1145 – 1149

Teschendorff AE (2019) Avoiding common pitfalls in machine learning omic data science. *Nat Mater* 18: 422 – 427

Thompson M, Hill BL, Rakocz N, Chiang JN, Sankararaman S, Hofer I, Cannesson M, Zaitlen N, Halperin E (2022) Methylation risk scores are associated with a collection of phenotypes within electronic health record systems. *medRxiv* https://doi.org/10.1101/2022.02.07.22270047 [PREPRINT]

Tian Y, Morris TJ, Webster AP, Yang Z, Beck S, Feber A, Teschendorff AE (2017) ChAMP: updated methylation analysis pipeline for Illumina BeadChips. *Bioinformatics* 33: 3982 – 3984

Yousefi PD, Suderman M, Langdon R, Whitehurst O, Davey Smith G, Relton CL (2022) DNA methylation-based predictors of health: applications and statistical considerations. *Nat Rev Genet* 23: 369 – 383

Zhou S, Zhang J, Xu J, Zhang F, Li P, He Y, Wu J, Wang C, Wang X, Zhang W *et al* (2021) An epigenome-wide DNA methylation study of patients with COVID-19. *Ann Hum Genet* 85: 221 – 234

Appendix 1

Biobank Team Members

Vanessa Barcessat, Precision Immunology Institute, Icahn School of Medicine at Mount Sinai, New York, NY, USA; Kevin Tuballes, Precision Immunology Institute, Icahn School of Medicine at Mount Sinai, New York, NY, USA; Diane Marie Del Valle, Precision Immunology Institute, Icahn School of Medicine at Mount Sinai, New York, NY, USA; Kai Nie, Human Immune Monitoring Center (HIMC), Icahn School of Medicine at Mount Sinai, New York, NY, USA; Hui Xie, Human Immune Monitoring Center (HIMC), Icahn School of Medicine at Mount Sinai, New York, NY, USA; Grace Chung, Human Immune Monitoring Center (HIMC), Icahn School of Medicine at Mount Sinai, New York, NY, USA; Manishkumar Patel, Human Immune Monitoring Center (HIMC), Icahn School of Medicine at Mount Sinai, New York, NY, USA; Jocelyn Harris, Human Immune Monitoring Center (HIMC), Icahn School of Medicine at Mount Sinai, New York, NY, USA; Kimberly Argueta, Human Immune Monitoring Center (HIMC), Icahn School of Medicine at Mount Sinai, New York, NY, USA; Jacques Fehr, Precision Immunology Institute, Icahn School of Medicine at Mount Sinai, New York, NY, USA; Barr Gruberg, Human Immune Monitoring Center (HIMC), Icahn School of Medicine at Mount Sinai, New York, NY, USA; Nicholas Zaki, Human Immune Monitoring Center (HIMC), Icahn School of Medicine at Mount Sinai, New York, NY, USA.

License: This is an open access article under the terms of the Creative Commons Attribution License, which permits use, distribution and reproduction in any medium, provided the original work is properly cited.

© 2023 The Authors

Expanded View Figures

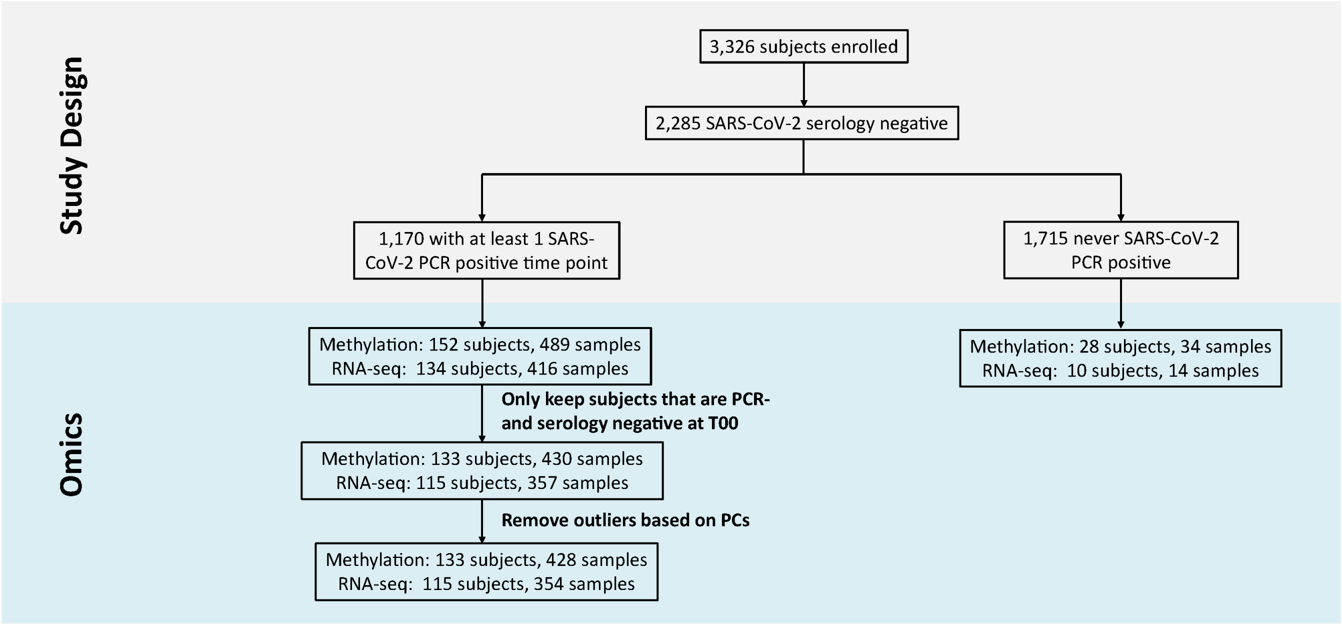

	Subject-wise		Sample-wise	
	**Methylation**	**RNA-seq**	**Methylation**	**RNA-seq**
**Total**	133	115	428	354
**Gender**	Male: 122<br>Female: 11	Male: 104<br>Female: 11	Male: 397<br>Female: 31	Male: 325<br>Female: 29
**Age**	Overall: 19.77 ± 2.45<br>Male: 19.81 ± 2.52<br>Female: 19.36 ± 1.57	Overall: 19.88 ± 2.58<br>Male: 19.93 ± 2.66<br>Female: 19.36 ± 1.57	n.a.	n.a.
**Race**	White: 94<br>Black: 21<br>Multi-racial: 5<br>AI/AN: 3<br>Asian: 3<br>Hawaiian/OPI: 1<br>Other: 1<br>NA: 5	White: 80<br>Black: 19<br>Multi-racial: 4<br>AI/AN: 3<br>Asian: 3<br>Other: 1<br>NA: 5	White: 306<br>Black: 64<br>Multi-racial: 14<br>AI/AN: 11<br>Asian: 8<br>Hawaiian/OPI: 4<br>Other: 3<br>NA: 18	White: 252<br>Black: 55<br>Multi-racial: 10<br>AI/AN: 10<br>Asian: 8<br>Other: 3<br>NA: 16
**Ethnicity**	Non-Hispanic: 57<br>Hispanic: 44<br>NA: 32	Non-Hispanic: 48<br>Hispanic: 39<br>NA: 28	Non-Hispanic: 172<br>Hispanic: 147<br>NA: 109	Non-Hispanic: 136<br>Hispanic: 124<br>NA: 94
**Symptom**	Asymptomatic: 68<br>Symptomatic: 65	Asymptomatic: 60<br>Symptomatic: 55	n.a.	n.a.

***n.a.** Not Applicable; **NA** Not Available

Figure EV1. CHARM study description.

Participants and samples are summarized by gender, race, ethnicity, and reported symptoms. All analyses of methylation changes associated with SARS-CoV-2 infection used preinfection samples as the Control group. The methylation data from the 28 never infected participants were used for the model evaluation of this group shown in Fig 4C. n.a., not applicable; NA, not available.

© 2023 The Authors

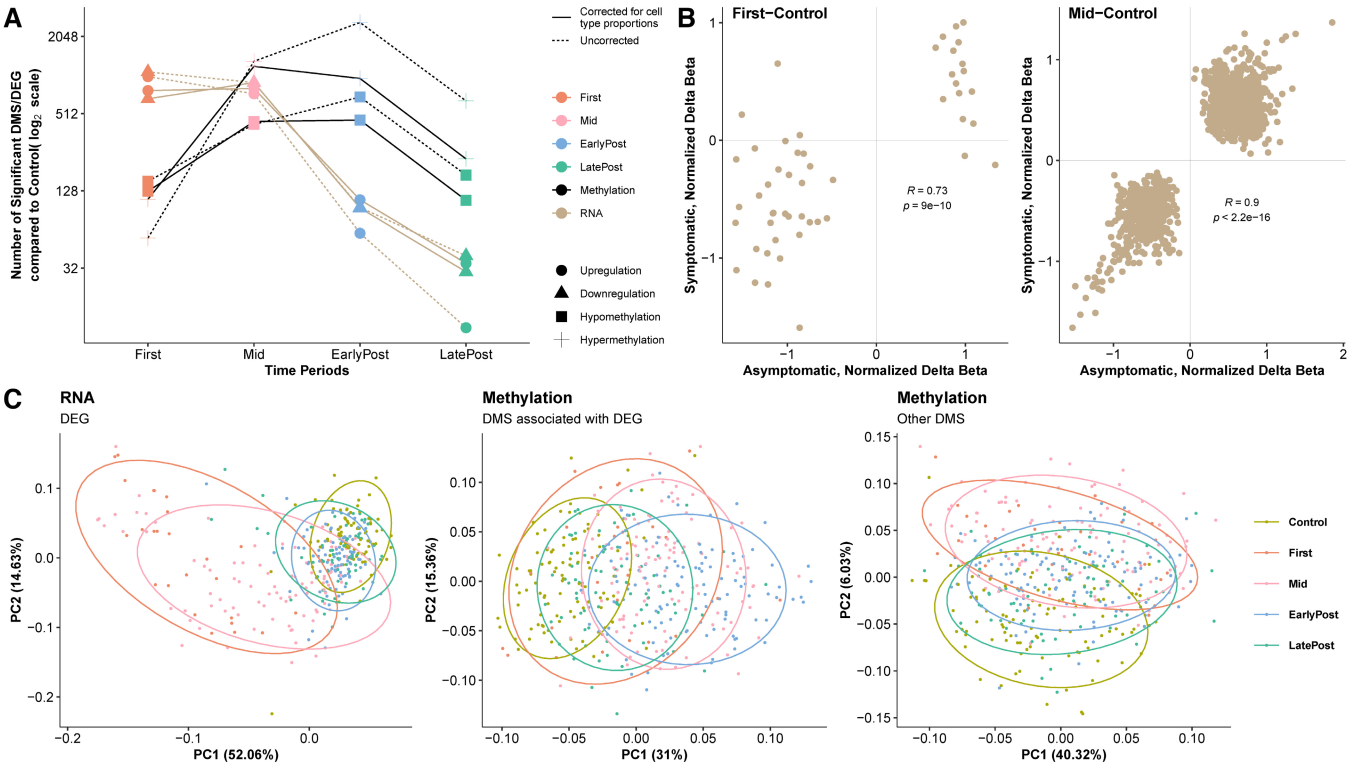

Figure EV2. Relationship of gene and methylation changes following SARS-CoV-2 infection.

A The number of differentially expressed genes (DEG) and differentially methylated sites (DMS) during each infection period compared with preinfection levels (uncorrected $P < 1e^{-4}$) are plotted separately by direction of regulation. Analysis corrected for cell-type proportions and uncorrected are shown separately.

B Scatter plots comparing the changes in methylation levels compared with control following asymptomatic ($n = 68$) and mildly symptomatic ($n = 65$) infections for the First and Mid time period. These plots correspond to the same analysis shown for EarlyPost and LatePost in Fig 1C.

C Principal component analysis of the Mid vs. Control DEG or DMS (with FDR < 0.05 and fold change > 1.5 for DEG) at all time periods. Other DMS, unannotated DMS. These plots correspond to Fig 1D.

 © 2023 The Authors

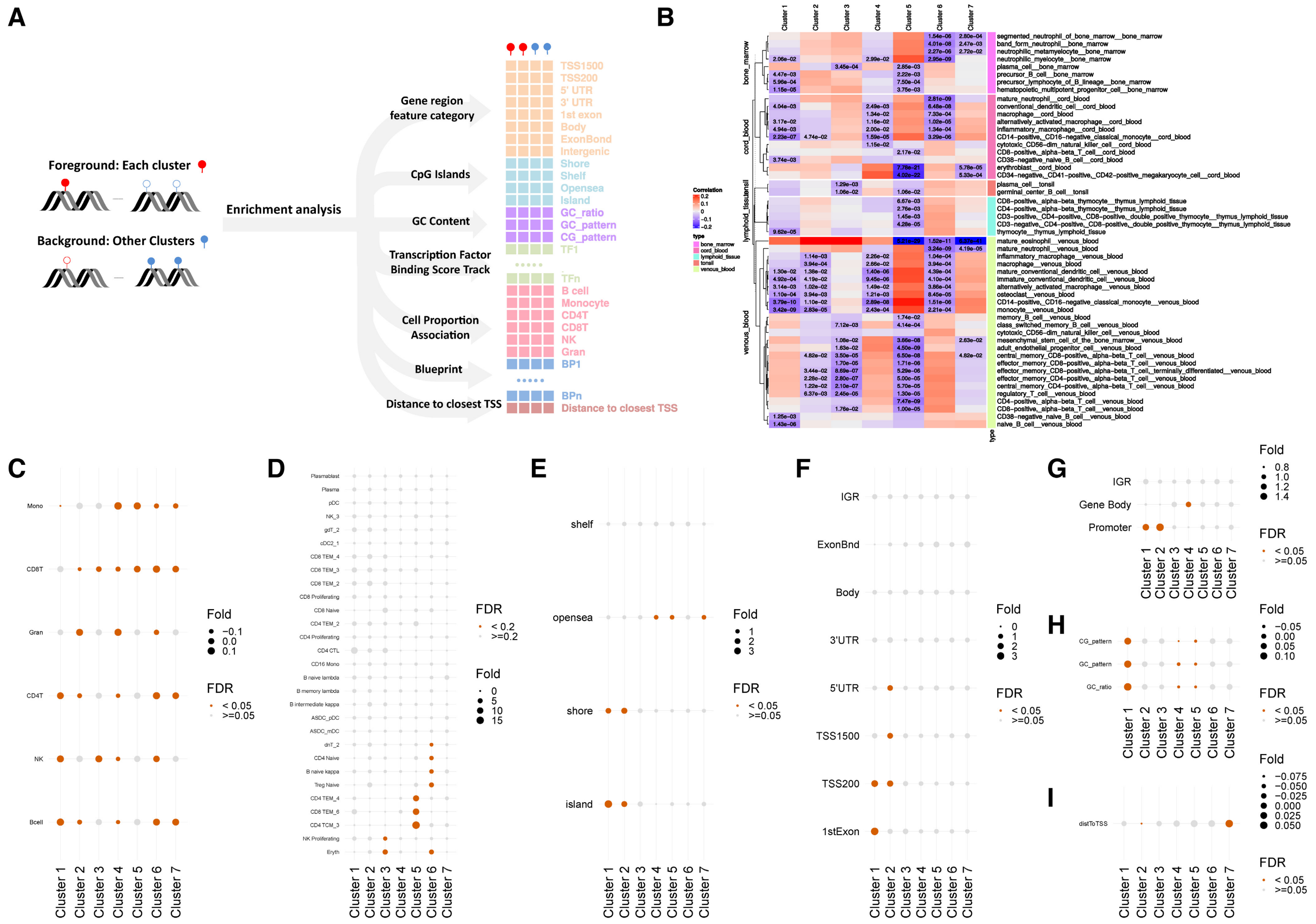

Figure EV3. Analysis of temporal clusters of differentially methylated sites.

- A Schematic showing the features evaluated by enrichment analysis for association with postinfection hypomethylated sites in each DMS cluster from Fig 2.
- B Correlation of DMS in each cluster with Blueprint cell-type methylation markers. (See Materials and Methods).
- C Enrichment analysis with respect to the Pearson correlations of DMS in each cluster with inferred cell-type proportions. Fold enrichment for each cluster is indicated in comparison with all clusters (see Materials and Methods).
- D Enrichment analysis showing the top five enriched cell markers from single-cell RNA-seq for DMS in each cluster. Cell markers with FDR < 0.05 are highlighted.
- E Enrichment analysis for CpG island categories. Results with FDR < 0.05 are highlighted.
- F Enrichment analysis for gene region feature categories. Results with FDR < 0.05 are highlighted.
- G Enrichment analysis of gene region feature categories aggregated into promoter region (TSS1500, TSS200, 1st Exon, 5'UTR) and gene body (3'UTR, Body, ExonBnd). IGR is also included. Results with FDR < 0.05 are highlighted.
- H Enrichment analysis of CG and GC content categories. Results with FDR < 0.05 are highlighted.
- I Enrichment analysis of distance to transcription start site (TSS). Results with FDR < 0.05 are highlighted.

Figure EV4. Data normalization and modeling procedures.

- A Schematic of the processing pipeline used for RNA-Seq data normalization.
- B Schematic of the processing pipeline used for Methylation data normalization.
- C Schematic of the procedure utilized for nested cross-validation of all machine learning models generated. The left panel indicates one outer iteration for developing the model M built from the training set. The right side gives the data summary derived from all outer iterations.

© 2023 The Authors

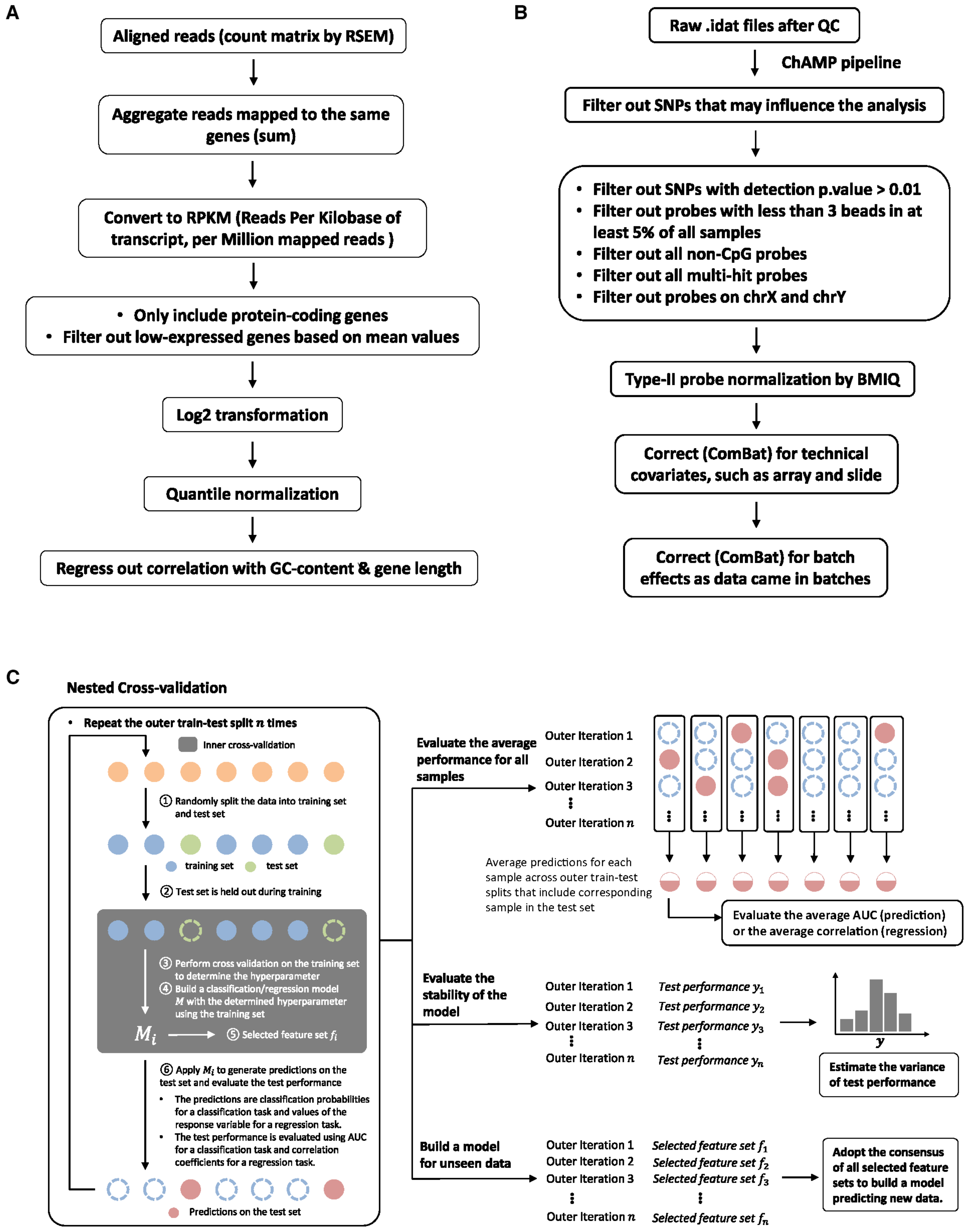
A
Aligned reads (count matrix by RSEM)
Aggregate reads mapped to the same genes (sum)
Convert to RPKM (Reads Per Kilobase of transcript, per Million mapped reads)
• Only include protein-coding genes
• Filter out low-expressed genes based on mean values
Log2 transformation
Quantile normalization
Regress out correlation with GC-content & gene length
B
Raw .idat files after QC
ChAMP pipeline
Filter out SNPs that may influence the analysis
• Filter out SNPs with detection p.value > 0.01
• Filter out probes with less than 3 beads in at least 5% of all samples
• Filter out all non-CpG probes
• Filter out all multi-hit probes
• Filter out probes on chrX and chrY
Type-II probe normalization by BMIQ
Correct (ComBat) for technical covariates, such as array and slide
Correct (ComBat) for batch effects as data came in batches
C
Nested Cross-validation
• Repeat the outer train-test split n times
Inner cross-validation
① Randomly split the data into training set and test set
training set test set
② Test set is held out during training
③ Perform cross validation on the training set to determine the hyperparameter
④ Build a classification/regression model M with the determined hyperparameter using the training set
M_i ⑤ Selected feature set f_i
⑥ Apply M_i to generate predictions on the test set and evaluate the test performance
• The predictions are classification probabilities for a classification task and values of the response variable for a regression task.
• The test performance is evaluated using AUC for a classification task and correlation coefficients for a regression task.
Predictions on the test set
Evaluate the average performance for all samples
Outer Iteration 1
Outer Iteration 2
Outer Iteration 3
Outer Iteration n
Average predictions for each sample across outer train-test splits that include corresponding sample in the test set
Evaluate the average AUC (prediction) or the average correlation (regression)
Evaluate the stability of the model
Test performance y_1
Test performance y_2
Test performance y_3
Test performance y_n
y
Estimate the variance of test performance
Build a model for unseen data
Selected feature set f_1
Selected feature set f_2
Selected feature set f_3
Selected feature set f_n
Adopt the consensus of all selected feature sets to build a model predicting new data.

Figure EV4.

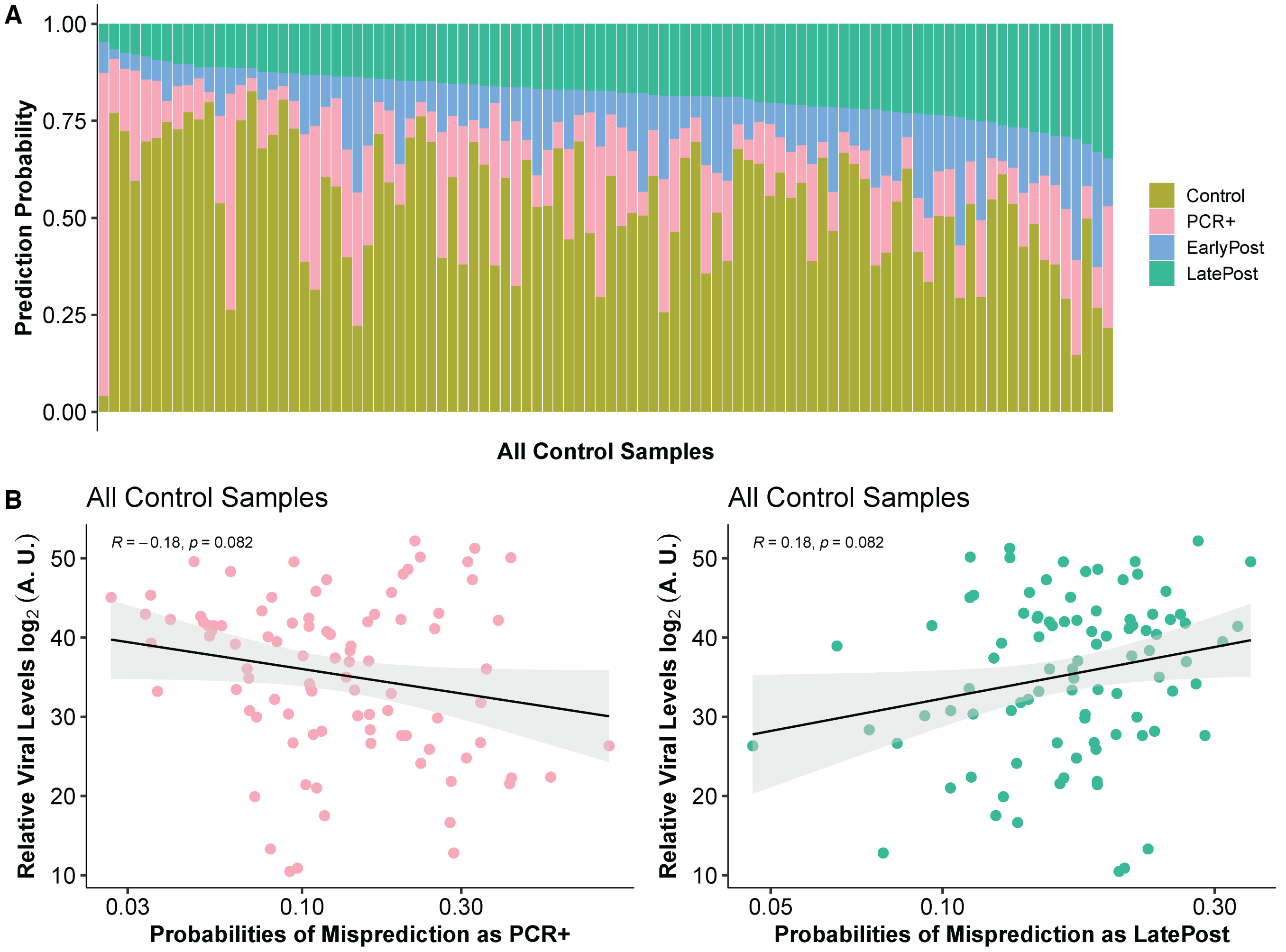

Figure EV5. Multiclass classifier predictions of control samples anticipate virus levels.

A Prediction probabilities generated by the multiclass classifier for all control samples are shown by bar plots. The results are in increasing order of the prediction probability obtained that each control sample is LatePost.

B Correlation plot of maximum relative viral levels measured during infection with the probabilities of misclassification as PCR-positive or LatePost using the classifier from Fig 3D of the control samples prior to infection from the same participants. A.U., arbitrary units calculated as 80-(minimum cycle threshold PCR result) for each participant.

Appendix to:

A methylation clock model of mild SARS-CoV-2 infection provides insight into immune dysregulation

Weiguang Mao, Clare M. Miller, Venugopalan D. Nair, Yongchao Ge, Mary Anne S. Amper, Antonio Cappuccio, Mary-Catherine George, Carl W. Goforth, Kristy Guevara, Nada Marjanovic, German Nudelman, Hanna Pincas, Irene Ramos, Rachel S. G. Sealfon, Alessandra Soares-Schanoski, Sindhu Vangeti, Mital Vasoya, Dawn L. Weir, Elena Zaslavsky, *Biobank team*, Seunghee Kim-Schulze, Sacha Gnjatic, Miriam Merad, Andrew G. Letizia, Olga G. Troyanskaya, Stuart C. Sealfon, Maria Chikina

Table of contents

1. **Appendix Figures S1-9** …………………………………………………… p.2-17
    - **Appendix Fig. S1** ……………………………………………………...…...... p.3-4
    - **Appendix Fig. S2**……………………………………………………..……..... p.5-6
    - **Appendix Fig. S3** ……………………………………………………..… p.7-8
    - **Appendix Fig. S4** ……………………………………………………...…. p.9-10
    - **Appendix Fig. S5** ……………………………………….………………. p.11
    - **Appendix Fig. S6** ……………………………………………..………....... p.12-13
    - **Appendix Fig. S7** ……………………….………………………………. p.14-15
    - **Appendix Fig. S8** ………………………………………...……………... p.16-17
    - **Appendix Fig. S9** ………………………………………………...………. p.17

2. **References** ………………………………………………………………...... p.18

1. Appendix Figures S1-9

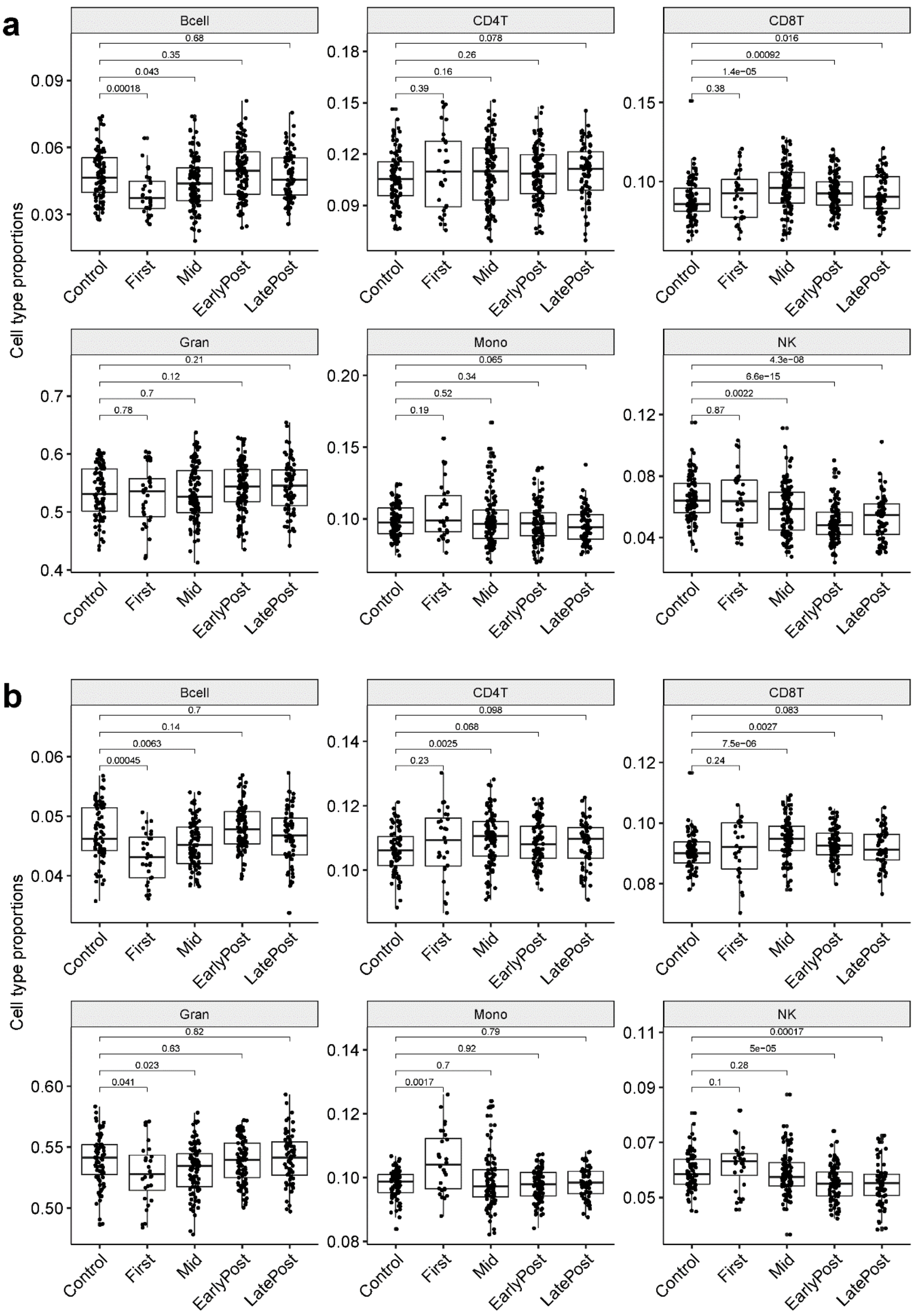

a
b
Cell type proportions
Bcell
CD4T
CD8T
Gran
Mono
NK
Control
First
Mid
EarlyPost
LatePost
0.68
0.35
0.043
0.00018
0.078
0.26
0.16
0.39
0.016
0.00092
1.4e−05
0.38
0.21
0.12
0.7
0.78
0.065
0.34
0.52
0.19
4.3e−08
6.6e−15
0.0022
0.87
0.7
0.14
0.0063
0.00045
0.098
0.068
0.0025
0.23
0.083
0.0027
7.5e−06
0.24
0.82
0.63
0.023
0.041
0.79
0.92
0.7
0.0017
0.00017
5e−05
0.28
0.1

Appendix Figure S1: Cell type proportion variation across periods relative to first PCR positive test. a, Boxplot of inferred cell type proportions based on all methylation samples. Cell type proportions at each period after infection were compared with pre-infection cell type proportions using two-tailed Wilcoxon rank sum test. b, Boxplot of inferred cell type proportions based on all RNA-seq samples. Cell type proportions at each period after infection were compared with pre-infection cell type proportions using two-tailed Wilcoxon rank sum test. See Methods for further information on this analysis.

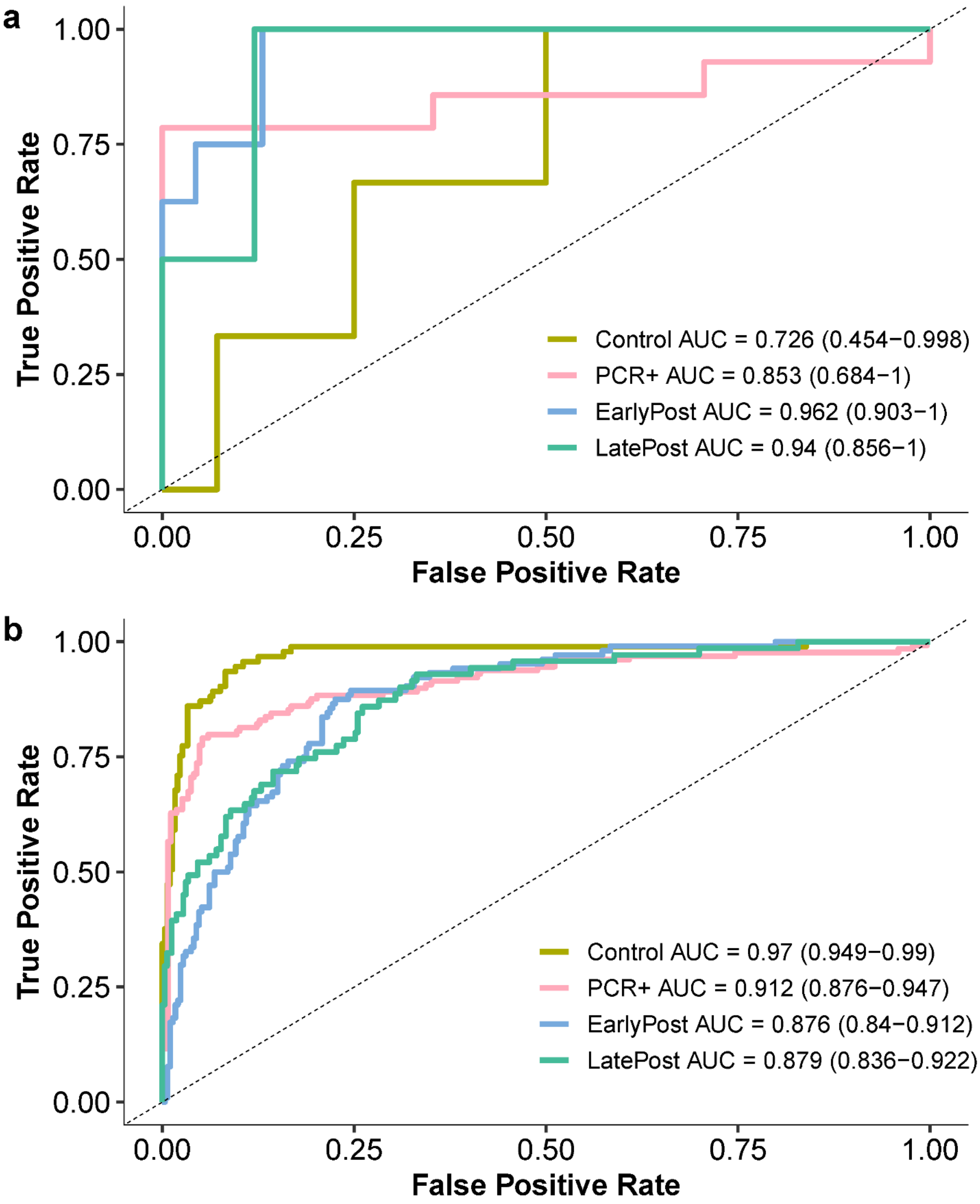
a
True Positive Rate
False Positive Rate
1.00
0.75
0.50
0.25
0.00
0.00
0.25
0.50
0.75
1.00
Control AUC = 0.726 (0.454−0.998)
PCR+ AUC = 0.853 (0.684−1)
EarlyPost AUC = 0.962 (0.903−1)
LatePost AUC = 0.94 (0.856−1)
b
True Positive Rate
False Positive Rate
1.00
0.75
0.50
0.25
0.00
0.00
0.25
0.50
0.75
1.00
Control AUC = 0.97 (0.949−0.99)
PCR+ AUC = 0.912 (0.876−0.947)
EarlyPost AUC = 0.876 (0.84−0.912)
LatePost AUC = 0.879 (0.836−0.922)

Appendix Figure S2: Comparison of multiclass classifier performance on samples from male and female participants. a, Receiver operator curve obtained from multiclass classifier applied to samples from female participants. The 95% confidence intervals are indicated in the key. **b**, Receiver operator curve obtained from multiclass classifier applied to samples from male participants. The 95% confidence intervals are indicated in the key.

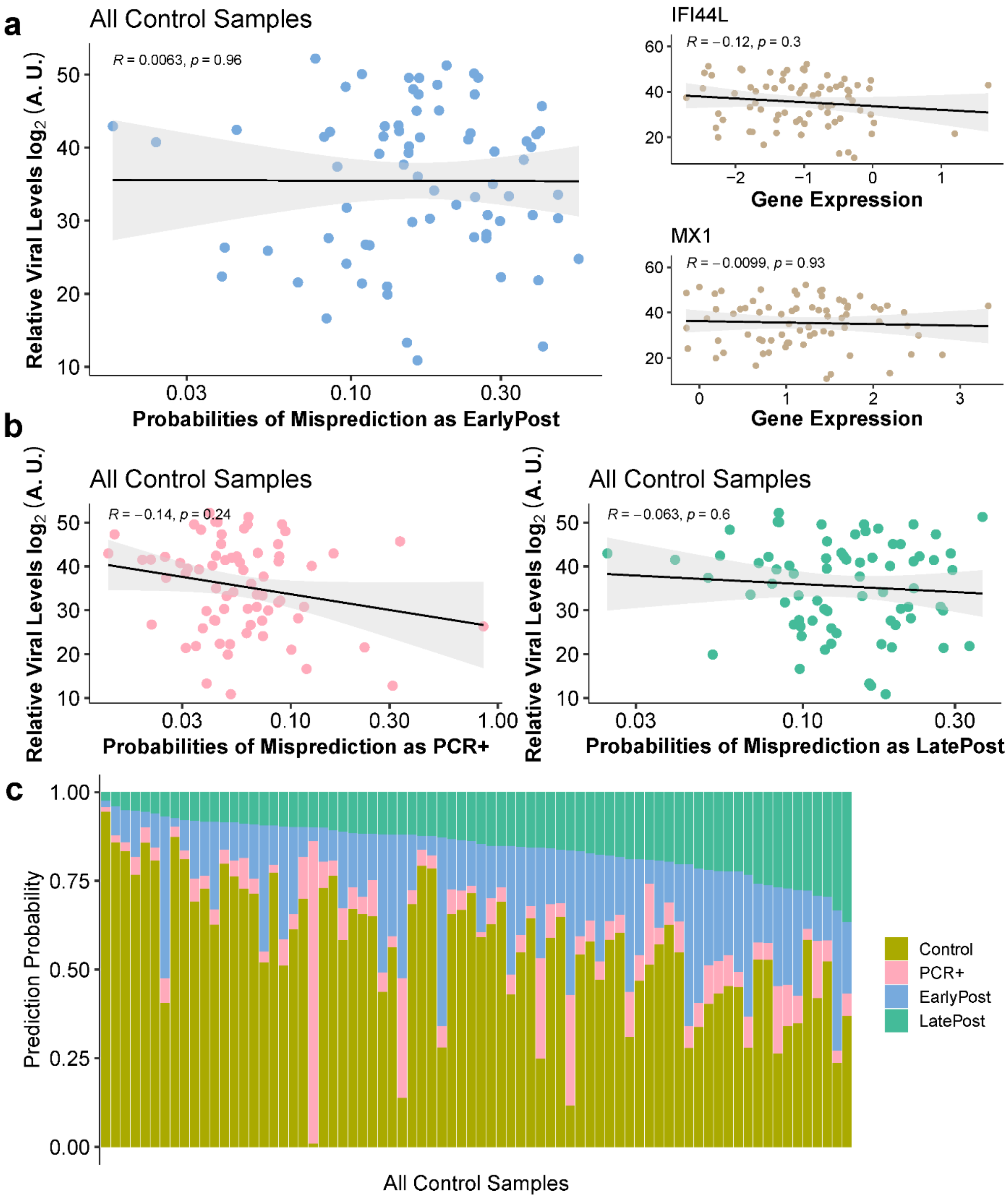

Appendix Figure S3: Gene expression-based multiclass classifier predictions of control samples do not anticipate virus levels. a, Correlation between maximum relative viral level during infection and i) the probabilities of misclassification as EarlyPost (*Left*) using the gene

expression-based multiclassifier model built in the same way as Figure 5a; ii) two upregulated genes, with uncorrected p values (*Right*). A.U., arbitrary units calculated as 80-(minimum cycle threshold PCR result) for each participant. **b**. Correlation plot of maximum relative viral levels measured during infection with the probabilities of misclassification as PCR-positive or LatePost using the classifier from panel a of the control samples prior to infection from the same participant. A.U., arbitrary unit calculated as 80-(minimum cycle threshold PCR result) for each participant. **c**. Prediction probabilities generated by the multiclass classifier for all control samples are shown by bar plot. The results are in increasing order of the prediction probability obtained that each control sample is LatePost.

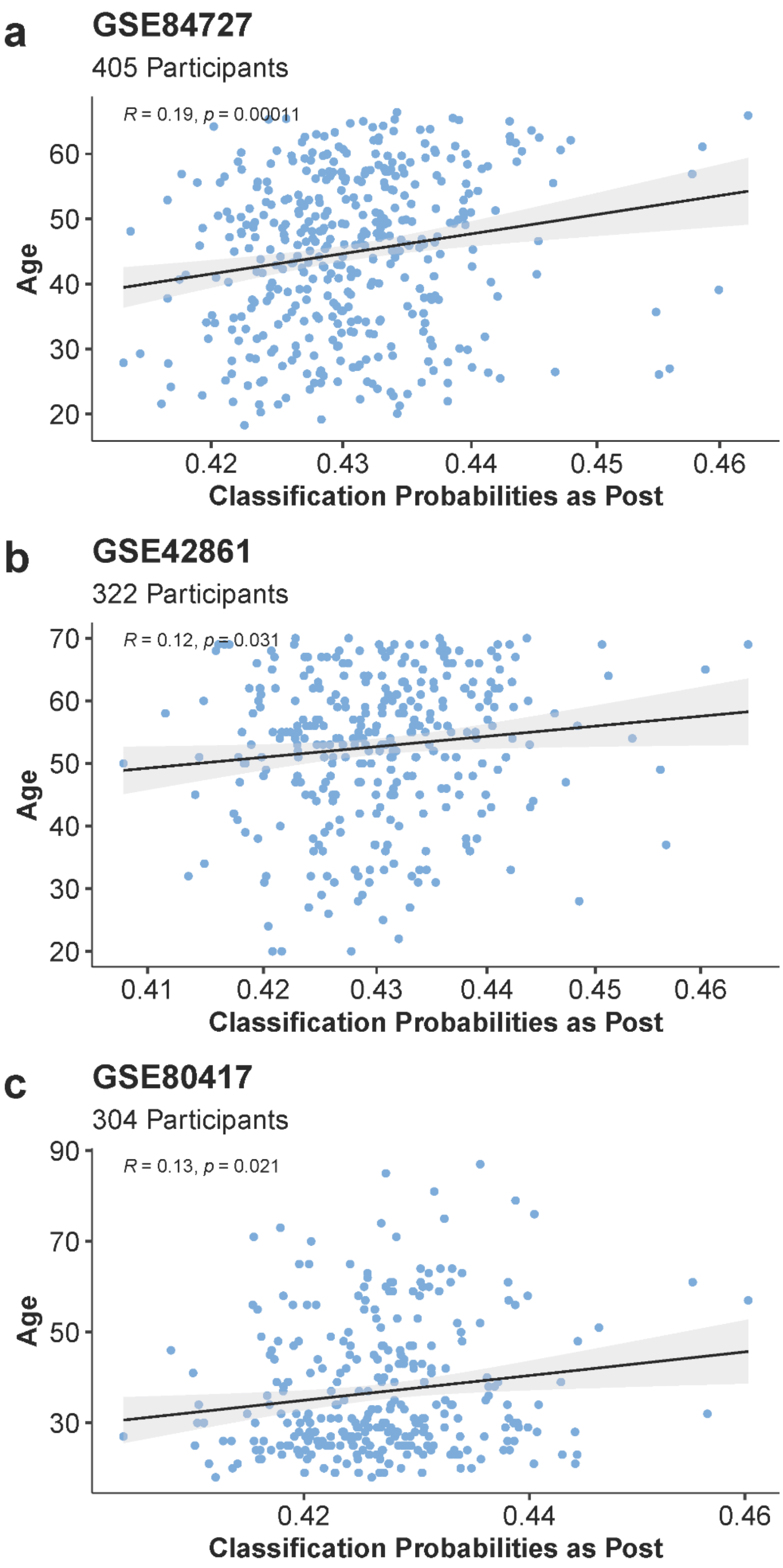
a
GSE84727
405 Participants
R = 0.19, p = 0.00011
Age
60
50
40
30
20
0.42
0.43
0.44
0.45
0.46
Classification Probabilities as Post
b
GSE42861
322 Participants
R = 0.12, p = 0.031
Age
70
60
50
40
30
20
0.41
0.42
0.43
0.44
0.45
0.46
Classification Probabilities as Post
c
GSE80417
304 Participants
R = 0.13, p = 0.021
Age
90
70
50
30
0.42
0.44
0.46
Classification Probabilities as Post

Appendix Figure S4. Post-infection probabilities are associated with age. Applying the multiclass classifier on several cohorts of methylation data shows a significant positive correlation between age and prediction probabilities as Post.

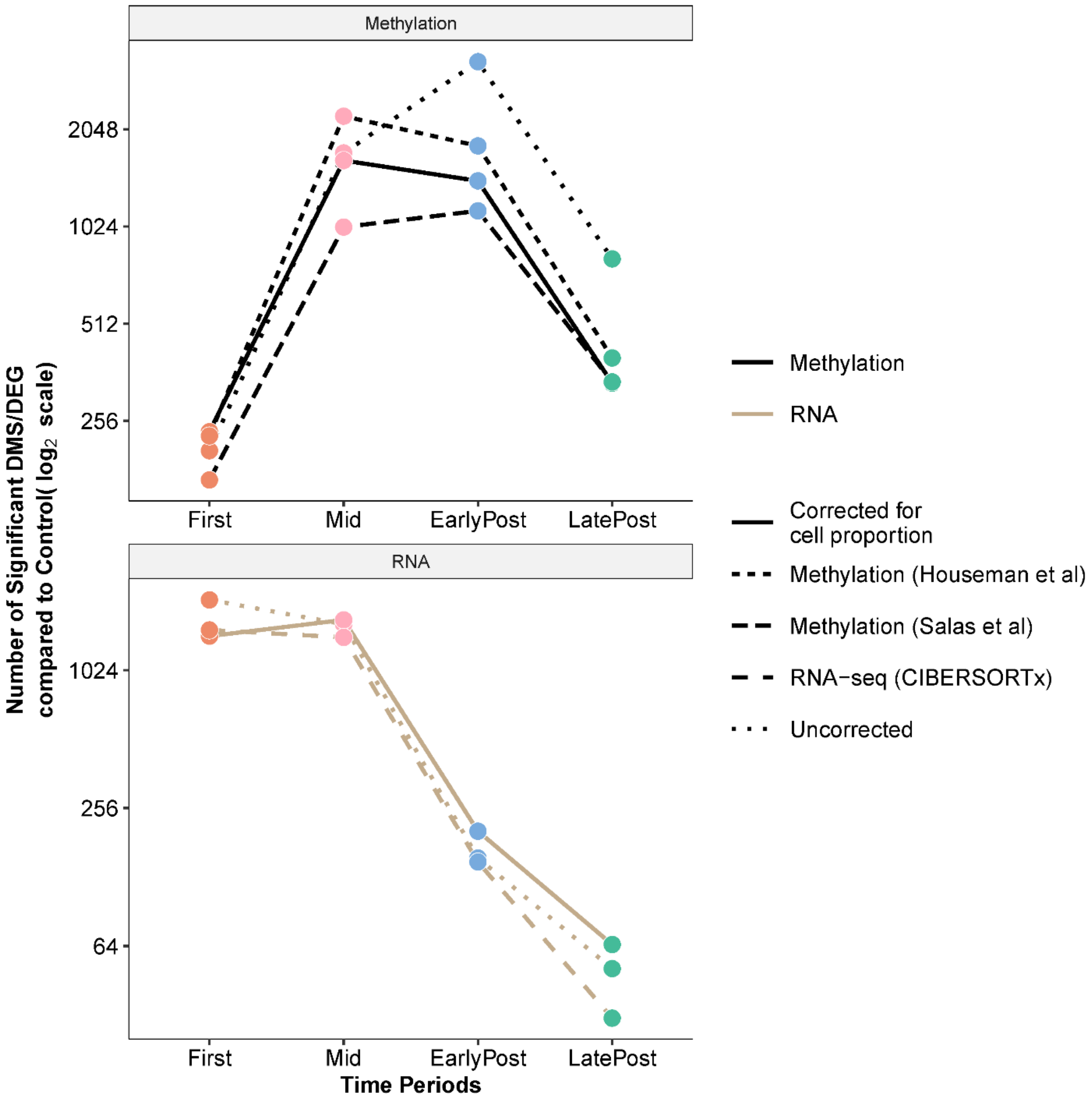

Appendix Figure S5: Timing of methylation and RNA changes using different proportion estimation methods. Number of DMS or DEG in each pseudotime period vs. pre-infection controls (nominal p<10-4). Numbers were either corrected for cell type proportions using the methods indicated (Houseman *et al*, 2012; Newman *et al*, 2019; Salas *et al*, 2022) or uncorrected. See also Figure 1b, Figure EV2 and Appendix Figure S6.

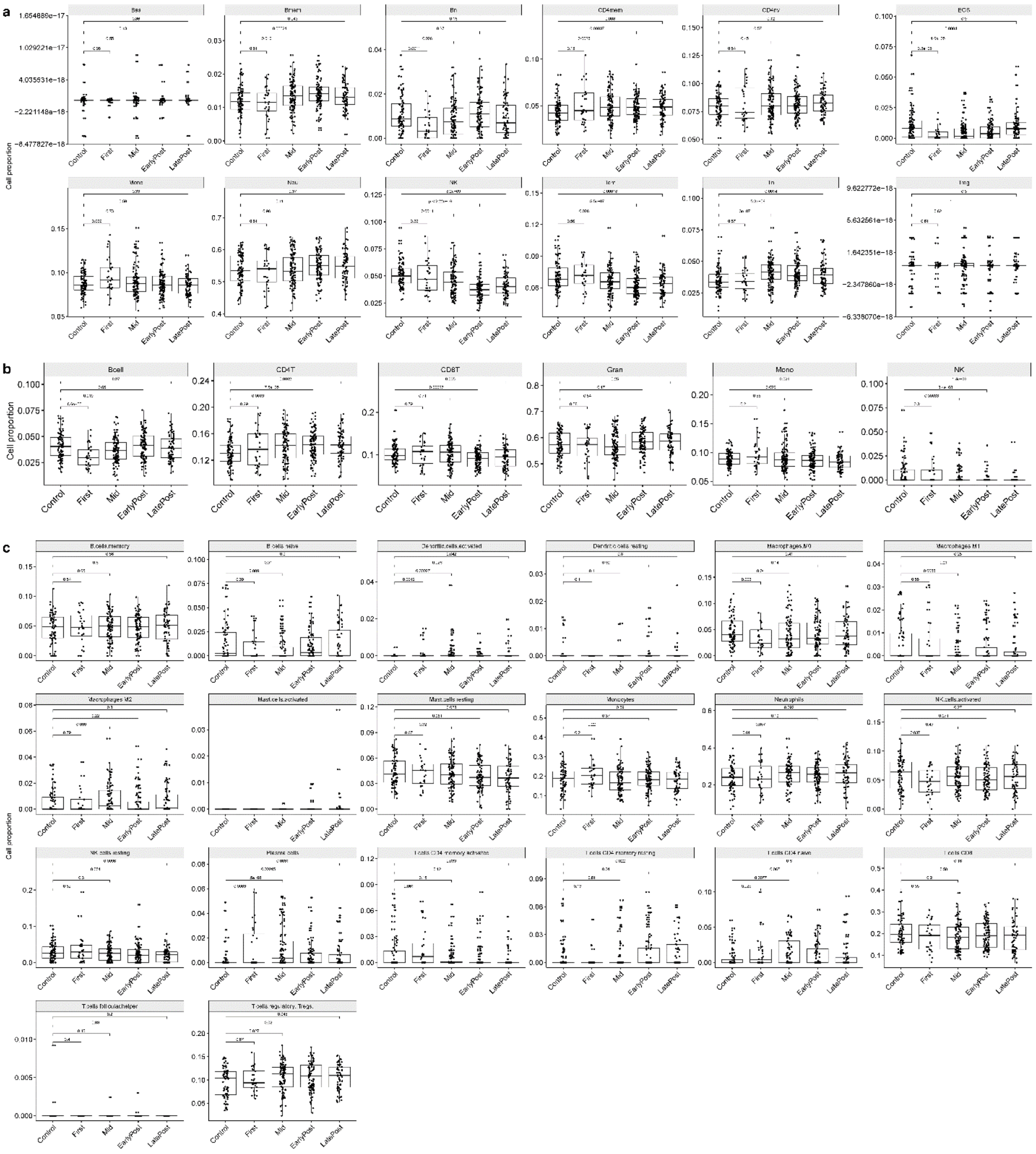

Appendix Figure S6. Visualization of cell proportion differences across time points. Cell type proportion variation across intervals relative to the first PCR positive test in the young adult longitudinal study. Cell type proportions at each period after infection were compared with pre-infection cell type proportions using two-tailed Wilcoxon rank sum test. **a**, Boxplot of inferred

cell type proportions based on all methylation samples and the deconvolution basis was from the Salas et al. (Salas *et al.*, 2022). **b**, Boxplot of inferred cell type proportions based on all methylation samples and the deconvolution basis was from Houseman et al. (Houseman *et al.*, 2012). **c**, Boxplot of inferred cell type proportions based on all RNA-seq samples and the deconvolution basis was the LM22 signature matrix from CIBERSORTx (Newman *et al.*, 2019).

a

Name	Normalization Pipeline	SSE
Raw	No regression	/
B2M2B (current)	Raw → converted to M value → regress out the cell type proportion → converted to beta values.	29850
B_regress	Raw → regress out the cell type proportion directly in the beta value space	30730

b

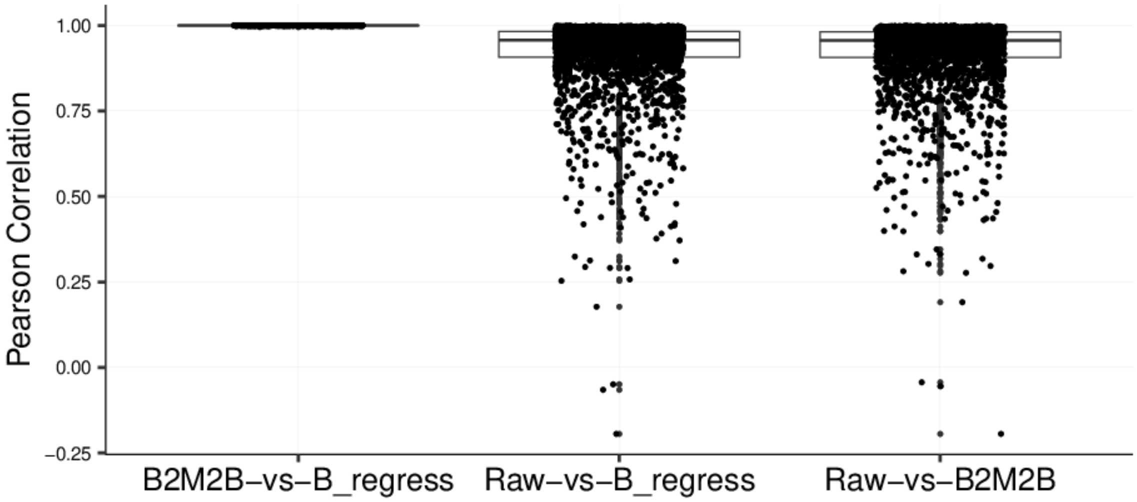

c

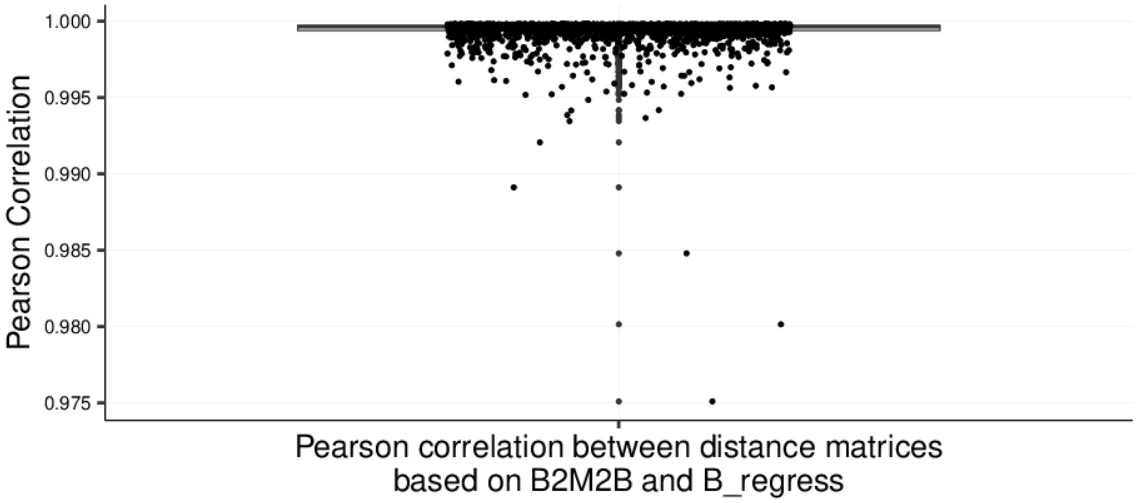

Appendix Figure S7. B2M2B and B_regress are highly correlated as two different normalization approaches to eliminate the cell type proportion variations as confounders. **a**, Detailed normalization flowcharts for B2M2B and B_regress. Total within-cluster variance (SSE) for each approach is provided in the last column. **b**, Pearson correlations between

corresponding methylation samples across all 707,361 CpGs following different normalization pipelines. **c**, Comparison of distance matrices (dynamic warping distance), the measure used for the clustering in Fig. 2A, calculated using B2M2B and B_regress.

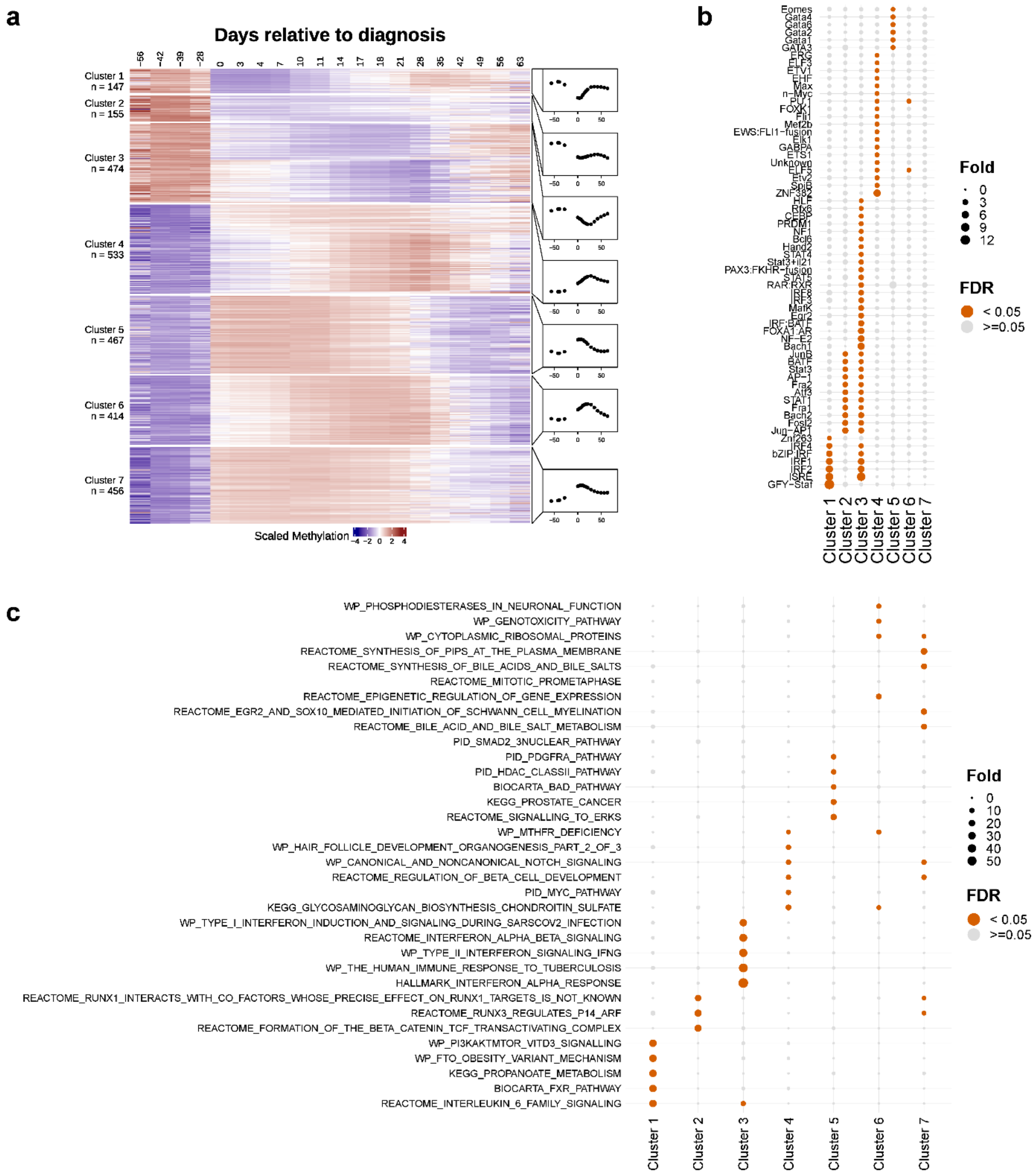

Appendix Figure S8. Characteristics of differential methylation following SARS-CoV-2 infection using B_regress. This is a remake of Figure 2 in the manuscript using the B_regress analysis **a**, Z-scored levels at DMS clustered by temporal trajectory relative to the first PCR-

positive test. Plotted is the average of each cluster over time. **b**, Enrichment of TFBS by cluster within a 200-bp window centered at each DMS, FDR <.05 for at least one cluster. Fold, fold enrichment. **c**, Top five pathways showing enrichment of DMS-associated genes in each cluster. b,c, FDR <.05 for at least one cluster. Fold, fold enrichment. Unlike the results obtained with the B2M2B analysis shown in Fig. 2, the TF enrichment indicates that the results with B_regress are clusters that have less biological coherence. Using B_regress, as shown above, there is less distinction of the TFs between clusters, and several of the clusters in the TF enrichment lack any annotation at all. In the B2M2B analysis shown in main Fig. 2, the clusters are tighter (see Fig. S7A) and are more biologically distinct, as assessed by the TF enrichment analysis.

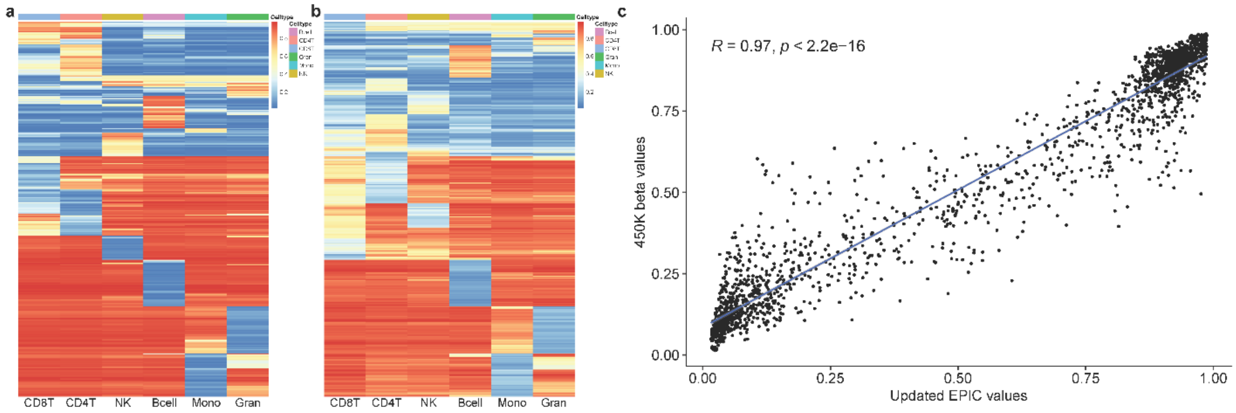

Appendix Figure S9. Updated basis matrix for the EPIC array is highly correlated with the standard Houseman et al. basis. a, Heatmap of the updated basis matrix for the EPIC array. **b**, For comparison, the heatmap using the standard Houseman et al. (Houseman *et al*, 2012) basis for the 450K array is shown. The update basis matrix generates somewhat better cell type discrimination **c**, Scatter plot of the 450K values against the updated EPIC basis values are highly correlated.

2. References

Houseman EA, Accomando WP, Koestler DC, Christensen BC, Marsit CJ, Nelson HH, Wiencke JK, Kelsey KT (2012) DNA methylation arrays as surrogate measures of cell mixture distribution. *BMC Bioinformatics* 13: 86

Newman AM, Steen CB, Liu CL, Gentles AJ, Chaudhuri AA, Scherer F, Khodadoust MS, Esfahani MS, Luca BA, Steiner D *et al* (2019) Determining cell type abundance and expression from bulk tissues with digital cytometry. *Nat Biotechnol* 37: 773-782

Salas LA, Zhang Z, Koestler DC, Butler RA, Hansen HM, Molinaro AM, Wiencke JK, Kelsey KT, Christensen BC (2022) Enhanced cell deconvolution of peripheral blood using DNA methylation for high-resolution immune profiling. *Nat Commun* 13: 761
