# Supplementary material for: A methylation clock model of mild SARS‐CoV‐2 infection provides insight into immune dysregulation
Source: Mol Syst Biol. 2023 Mar 15;19(5):e11361. doi: 10.15252/msb.202211361 (PMC10167476; doi:10.15252/msb.202211361)
Supplement: Supplementary file 15 [file MSB-19-e11361-s007.pdf]

molecular systems biology

A methylation clock model of mild SARS-CoV-2 infection provides insight into immune dysregulation

Weiguang Mao, Clare Miller, Venugopalan Nair, Yonchao Ge, Mary Anne Amper, Antonio Cappuccio, Mary-Catherine George, Carl Goforth, Kristy Guevara, Nada Marjanovic, German Nudelman, Hanna Pincas, Irene Ramos, Rachel Sealfon, Alessandra Soares-Schanoski, Sindhu Vangeti, Mital Vasoya, Dawn Weir, Elena Zaslavsky, Biobank Team, Seunghee Kim-Schulze, Sacha Gnjatic, Miriam Merad, Andrew Letizia, Olga Troyanskaya, Stuart Sealfon, and Maria Chikina
DOI: 10.15252/msb.202211361

Corresponding author(s): Stuart Sealfon (stuart.sealfon@mssm.edu) , Maria Chikina (mchikina@pitt.edu)

Review Timeline:

Submission Date:	23rd Sep 22
Editorial Decision:	27th Oct 22
Revision Received:	1st Dec 22
Editorial Decision:	18th Jan 23
Revision Received:	16th Feb 23
Accepted:	20th Feb 23

Editor: Jingyi Hou

Transaction Report:

(Note: With the exception of the correction of typographical or spelling errors that could be a source of ambiguity, letters and reports are not edited. Depending on transfer agreements, referee reports obtained elsewhere may or may not be included in this compilation. Referee reports are anonymous unless the Referee chooses to sign their reports.)

1st Editorial Decision **27th Oct 2022**

Thank you for submitting your work to Molecular Systems Biology. We have now heard back from the three reviewers who agreed to evaluate your study. As you will see below, the reviewers find your study of interest. However, they raise substantial concerns about your work, which should be convincingly addressed in a major revision of the present manuscript.

Without reiterating all the points listed below, the most fundamental issues that need to be convincingly addressed are the following:

- Reviewer #3 raised significant concerns about data analysis and statistical implementation, which must be carefully addressed.
- Reviewer #2's concerns about the conclusiveness of the main findings (especially major points #1 and #2) should be satisfactorily addressed. Several related statements should be better supported and more carefully worded to avoid overstatements.

All other issues raised by the reviewers need to be satisfactorily addressed as well. Please feel free to contact me in case you would like to discuss in further detail any of the issues raised by the reviewers.

Reviewer #1:

This is an interesting study in which the authors investigate the impact of a mild or asymptomatic COVID-19 infection on the long-term DNA methylation pattern in the blood of the patients during and after the infection. The authors identify transcriptional changes during infections, and in parallel DNA methylation changes with a slower kinetics. Interestingly, it is only the DNA methylation that persists long-term after the infection. Moreover, the post-infection patterns seem to be association with an anti-protective effect against SARS-CoV-2 infection.

Comments

1. The study is very interesting and relevant, and the methodology is appropriate. I would only suggest a bit more caution in the final conclusions of the study. In this respect, "Probabilities of these samples being misclassified as active infection or LatePost were not significantly associated with viral load (Fig. EV7b)". It was only the "probabilities of the pre-infection samples being misclassified as EarlyPost" that were associated with having higher maximal levels of virus detected by PCR (Page 9). Thus, it should be concluded that this deleterious effect of the COVID-19 infection is relatively short lived, although certainly important. This should be clearly discussed.
2. COVID-19 is not the only infection which is associated with an increased susceptibility to other infections after recovery: other examples are sepsis or measles, although the impact of epigenetic changes after these infections has not been studied. Nevertheless, it would be good to discuss these other infections.
3. It would have been ideal to be able to assess whether in addition to viral loads associated with the post-COVID19 DNA methylation patterns, there are also associations with markers of systemic inflammation (e.g. CRP, NLR, ferritin, etc), known to contribute to severity of the disease. Is such information available?
4. Can the authors speculate whether the susceptibility to other infections than COVID-19 might be influenced by the long-term changes in DNA-methylation.

Reviewer #2:

In this manuscript, the authors first observed a persistent methylation but not gene expression pattern post asymptomatic or mildly symptomatic SARS-CoV-2 infection by comparing methylation and gene expression profile of patients and control state through the course of the disease in the CHARM study cohort. The prolonged methylation is clustered into seven groups, each is enriched with distinct biological contents. Then, using the methylation data, a regression model was built to predict days post infection. The authors also trained classification models to investigate whether the methylation pattern could be applied to predict other disease state. Finally, the methylation data indicated that a post-infection like methylation profile was anti-protective and associated with poor prognosis regardless of gender. Such methylation seems cumulative, since post-infection like pattern becomes more obvious as age increases. It exerts prolonged effect, and is distinct from previous defined trained immunity. However, despite of intriguing results, there are major problems in article structure and data interpretation.

Major.
1. The major conclusion is not convincing.
(1) ... In other words, the control samples could already be in a post-infection-like state, for example as a result of infection with a different infectious agent or another immune challenge such as vaccination. According to the authors, the post-infection-like methylation pattern has an anti-protective effect (Fig. 5b). Patients who underwent SARS-Cov-2 infection (LatePost) should establish such methylation pattern, and thus should exhibit the same anti-protective effect. However, the probability of misclassifying a control sample as LatePost does not correlate negatively with relative viral levels (Fig EV7b, right). Why?

(2) The authors assumed that anti-protective methylation was related with innate immunity (... for example as a result of infection with a different infectious agent or another immune challenge such as vaccination.). However, the methylation data is heavily correlated with adaptive immunity (B & T cells, Fig. EV4b). Therefore, the methylation data may reflect the activity of adaptive immunity in each patients. Each control sample may vary in the activity of adaptive immunity intrinsically, and those who resemble EarlyPost state have a more active adaptive immunity preset, resulting low viral loads. If the authors want to conclude that anti-protective methylation comes from previous infection/stimulation, more solid biological data, rather than mathematical manipulation, is needed. Otherwise, it is circular argument.

2. Inconsistent article structure.
In the part "SARS-CoV-2 methylation clock", the authors trained a regression model to predict days post infection. These contents are disconnected from the remaining parts of the article and thus unnecessary.

3. Improper data interpretation.
In the part "Relationship to other conditions", the classification model is applied in a variety of disease types, such as autoimmune diseases (SLE, multiple sclerosis and rheumatoid arthritis), acute infection (SARS-Cov-2 and influenza) and chronic infection (HCV), which varied greatly in mechanism and phenotype and thus it is not reasonable to generalize the classification model. For example, ... Significant enrichment was observed between EarlyPost period DMS and the HCV study, an HIV study and two SLE studies. How to explain the resemblance? Is the same enrichment observed in other chronic or autoimmune diseases? What are the authors trying to imply with this model? Unless more biological interpretations are made, the generalization is meaningless.

Minor.
1. Among the top 16 CpG sites used by the model, we highlight two hypomethylated sites in IFI44L, which were individually inversely correlated with virus level (Fig. 5a): Should be Fig. 5b.
2. Can you define how long the anti-protective methylation pattern exist?
3. Since the authors mentioned train immunity, is methylation involved in the regulation of trained immunity? For example, what is the methylation level of IRF targeted genes?
4. Which type of cells play the major role in methylation-related anti-protection?

Reviewer #3:

The paper "A methylation clock model of mild SARS-CoV-2 infection provides insight into immune dysregulation" from Chikina and Sealfon's labs is a well-conducted longitudinal Covid-infection study of transcriptomic and DNA methylation (DNAm) patterns in a reasonable number of healthy young individuals (all males). The authors find that the severity of symptoms (in this cohort asymptomatic vs mild) did not have an impact on differential expression or DNAm patterns, but that there were differences between pre-infection, first and subsequent PCR+ tests and PCR- post-infection period. In particular, the authors discover that whilst gene expression patterns post-infection largely return to pre-infection levels, that DNAm levels do not. The authors find interesting enrichment of their DNAm signatures for interferon-related genes. The authors use elastic net ML-models to predict both time since infection as well as infection period, and are able to demonstrate the biological meaning of these models by applying it to other Covid-19 cohorts as well as other disease cohorts (mostly autoimmune), finding interesting

links with S.Lupus infection, and importantly finding that the level of the post-infection DNAm signature is predictive of Covid-19 severity in an independent cohort.
Overall, I think this is a well-designed and very interesting study, and contributes some important new findings. The figures are great. The authors have also used state-of-the-art statistical methodology throughout. However, there are also some very big concerns, especially with regards to the treatment of cell-type heterogeneity and the interpretation of the DNAm changes, as well as concerns regarding the statistical implementation of their prediction models, that absolutely need addressing:
1) Cell-type correction methodology is very poorly described and appears sub-standard (I): in Methods the authors write "We estimated the proportions of six major cell types (B cells, Granulocytes, Monocytes, NK cells, CD4 T cells and CD8 T cells) using standard reference-based method (Houseman et al, 2012). We took the original CellType450K basis matrix and replaced the values with those from (Roy et al, 2021) (Illumina Methylation microarray). This would help remove the bias induced by the platform inconsistency". As written, this does not make sense and seems to indicate that the authors don't understand how this works. First of all, the Houseman et al paper built a DNAm reference matrix for these cell-types using 450k technology. Approximately 90% of these 450k probes are present on the EPIC array, so in my experience the cell-type fractions estimated using Houseman's procedure using his DNAm reference matrix should work very well on EPIC data too. Indeed DNAm reference matrices can tolerate up to 30% missing values and still perform very well (see e.g. Zheng SC et al Epigenomics 2018). However, if the authors wish to maximize coverage, then you can't just replace values...that is wrong. What you need to do is build a new DNAm reference matrix using the EPIC DNAm data from say Roy et al 2021. But the authors do not describe that they did this, which is disturbing.
2) Cell-type correction methodology is very poorly described and appears sub-standard (II): In the same paragraph there is another problematic issue, which is that the authors adjust for cell-type heterogeneity by regressing M-values to the estimated cell-type fractions, and then seemingly transform back to beta-values. Irrespective of whether this procedure has been borrowed from another paper (in which case that paper should be revised...), it is wrong. M-values are unbounded, but fractions are bounded between 0 and 1. The methylation beta-value you measure is a linear sum of cell-type fractions times the methylation beta-values in the specific cell-types. So, the cell-type fractions appears naturally in the beta-value basis and therefore adjusting for cell-type fractions should be done in the beta-value basis. The residuals of course are not beta-valued but they also don't need to be. You can still use the residual matrix downstream. It would be incorrect for the authors to assume that they can have "a cell-type fraction adjusted data matrix" in the beta-valued basis. In theory what you actually want is the data matrix over CpGs and samples for each individual cell-type but this is an extremely hard problem and current methods don't work well.
3) Celltype fractions as covariates: the authors should also make it clearer when they are using the residuals, and when they are using them as covariates in their limma model. It should be clear that you don't need to do both together, that would be wrong. It is advised to use them as covariates in the linear models (limma) whenever possible.
4) Variations in celltype fractions and interpretation: I am a little puzzled regarding the interpretation of the derived DNAm signatures (Fig.2a) and their insensitivity to cell-type adjustment. On the one hand, Fig.EV2, clearly shows changes in cell-type proportions with infection period. For instance, B-cells and NK-cells display quite big changes. And yet, adjustment for cell-type fractions does not seem to alter the observed DNAm signatures? What about the enrichment of TFBSs in Fig.2b? Does that not point to specific cell-types and hence that these signatures are driven by corresponding changes in cell-type proportions? In other words, the authors don't seem to interpret any of their signatures in terms of variations in cell-type fractions, and frankly I find that hard to believe. It could be down to the fact that the authors have not adjusted for cell-type fractions in the correct manner (see previous points).
5) Estimation of celltype fractions with RNAseq: I don't understand what the authors did here. Frankly, by now there are many cell-type fraction estimation tools available for RNA-Seq data, including CIBERSORT. There are corresponding mRNA expression reference matrices. Just use that. Why train a model using paired DNAm data? What is the purpose of this? The authors are not pushing boundaries here in terms of deriving a new mRNA expression reference matrix for blood, so what is the point of this? The good agreement in Fig.EV2 between mRNA and DNAm is NOT impressive at all, if the mRNA-based estimates were trained using DNAm data. I find Fig.EV2a-b comparison to be very misleading in fact. Please change your mRNA-based method so that it only uses mRNA data!
6) Dynamic time warping: this is a nice method, but neither in Results nor Methods, do the authors explain what it actually does. Please add 1-2 sentences explaining what it actually does and why it was used in Fig.2a. We want authors to apply methods because they understand them, otherwise it gives the impression they are just applying a black-box. And please cite the corresponding paper in main text, not just in Methods.
7) Prediction models on external datasets: The authors use a rather unconventional method to build the models that are applied to the external datasets. If the authors used a nested CV on their training-test set splits, then why not run the internal CV loop on the full dataset to derive a prediction model that can then be applied to the external datasets? Or alternatively, because the authors have derived 100 classification/prediction models, one for each training-test set split, they could apply an ensemble approach where each of the 100 are tested on the external dataset. Prediction/classification scores can then be averaged to arrive a final prediction/classification score for each sample in the external dataset. These last two approaches are more conventional and rigorous than the ad-hoc approach of the authors.
8) Fig.1c-d: can the authors please confirm that in Fig.1c they have not removed any datapoints from the other two quadrants? I just found the agreement between asymptomatic and symptomatic to be amazingly good. As far as Fig.1d is concerned, I did not find this PCA panel to convey any useful message. I would remove it.
9) External datasets (Fig.EV6): On first mention in the main text, there are no details provided regarding these external datasets. What are they? And in Fig.EV6 I missed 95% confidence intervals for the AUCs specially in panel-A where the number of samples is quite low.
10) Fig.5b: the data displayed in this panel is not very convincing. The P-values are also quite marginal and some experts have

been arguing that significance thresholds should be lowered to <0.001 e.g. to account for multiple-testing of "tests". Moreover, the x-axis which labels the probability of misprediction only varies to 0.3?? I am surprised by that. I thought some samples would have a probability larger than 0.5, or why then are they "misclassified"?
11) Fig.3a: I am also very troubled by Fig.3a, because the x-axis values go up to 80 days after diagnosis, but the predicted times only go up to 40. So, if instead of using a correlation we were to use RMSE, we would have an extremely large error. I wonder if this large RMSE reflects an error or problem in the training of the regression model? Seems like it. Why not train the model to minimize the RMSE?

Minor point:
It is great to see that the authors used the correct nested CV strategy in their model training. It would be good if the authors could cite a few papers that have emphasized the importance of nested CV: Simon, R., et al J. Natl Cancer Inst. 95, 14-18 (2003) and Teschendorff et al Nat Materials 2019.

Reviewers' comments and our point-by-point responses

Reviewer #1

This is an interesting study in which the authors investigate the impact of a mild or asymptomatic COVID-19 infection on the long-term DNA methylation pattern in the blood of the patients during and after the infection. The authors identify transcriptional changes during infections, and in parallel DNA methylation changes with a slower kinetics. Interestingly, it is only the DNA methylation that persists long-term after the infection. Moreover, the post-infection patterns seem to be association with an anti-protective effect against SARS-CoV-2 infection.

Comments

1. The study is very interesting and relevant, and the methodology is appropriate. I would only suggest a bit more caution in the final conclusions of the study. In this respect, "Probabilities of these samples being misclassified as active infection or LatePost were not significantly associated with viral load (Fig. EV7b)". It was only the "probabilities of the pre-infection samples being misclassified as EarlyPost" that were associated with having higher maximal levels of virus detected by PCR (Page 9). Thus, it should be concluded that this deleterious effect of the COVID-19 infection is relatively short lived, although certainly important. This should be clearly discussed.

We appreciate the reviewers comment and have revised the discussion accordingly. It now reads (changes are highlighted on p.11-12, 2nd paragraph in Discussion):

Strikingly, we find that contrary to the trained immunity phenomenon, in this cohort the presence of an early post-infection-like methylation state prior to infection is anti-protective for the SARS-CoV-2 infection that occurred subsequent to these baseline measurements. This potentially deleterious effect of SARS-CoV-2 infection may be relatively short lived, as we found that the presence of a late post infection-like methylation state prior to infection showed only a non-significant trend towards being anti-protective. The persistence of SARS-CoV-2 induced methylation changes and their functional effects, if any, beyond the several months duration of the present investigation requires additional study.

We also now provide the results of the hospital based COVID outcome study split into early and late Post (see below) and find that those show a very similar pattern. Overall, the differences between earlyPost and latePost are subtle. We think the revision stating "may be relatively short lived" expresses what can be concluded from our analyses.

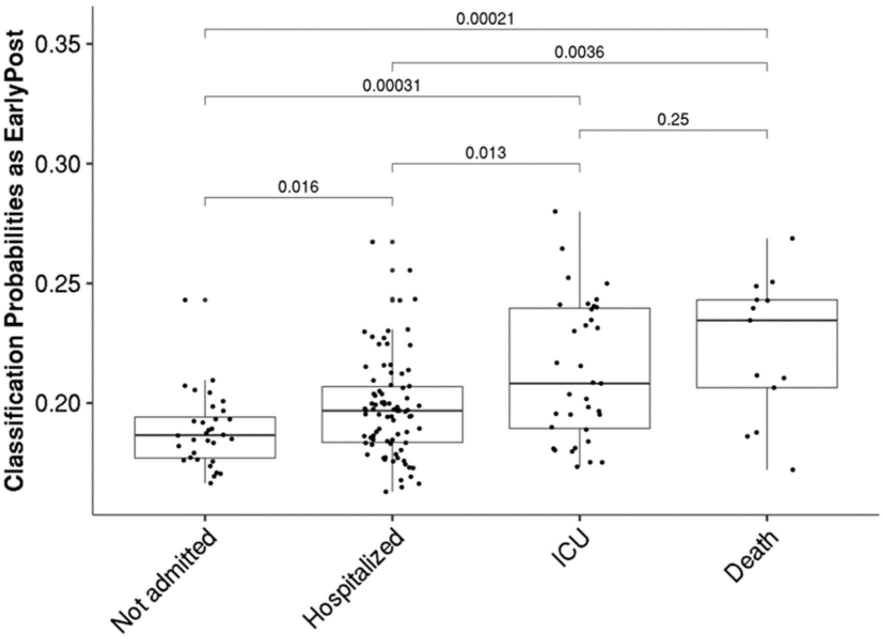

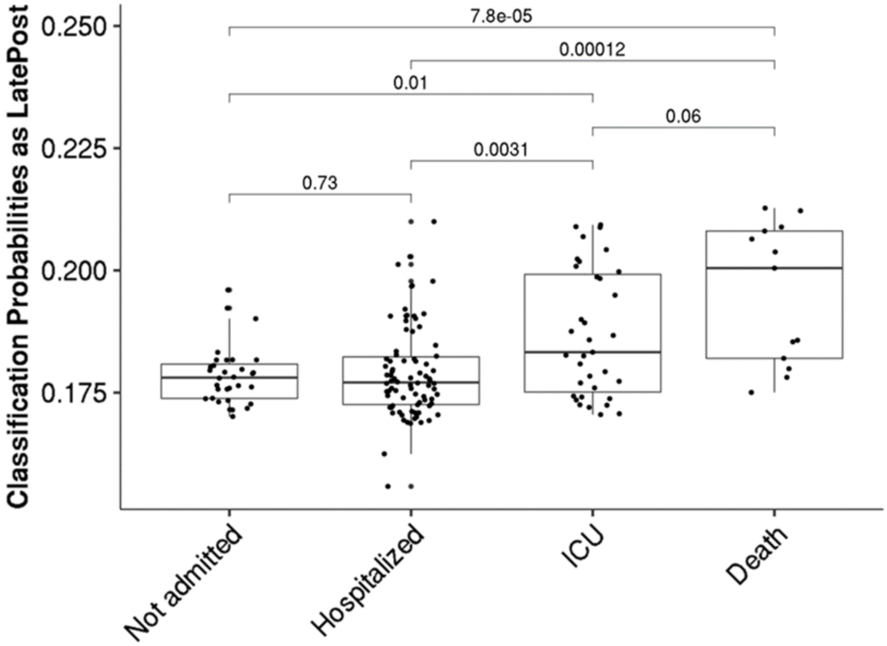

2. COVID-19 is not the only infection which is associated with an increased susceptibility to other infections after recovery: other examples are sepsis or measles, although the impact of epigenetic changes after these infections has not been studied. Nevertheless, it would be good to discuss these other infections.

We thank the reviewer for raising this point. We have revised the Discussion to include the statement (on p. 12, 2nd paragraph of Discussion):

"An increased subsequent infection risk has been observed following other primary infections, such as measles (PMID 32084116) "

3. It would have been ideal to be able to assess whether in addition to viral loads associated with the post-COVID19 DNA methylation patterns, there are also associations with markers of systemic inflammation (e.g. CRP, NLR, ferritin, etc), known to contribute to severity of the disease. Is such information available?

The reviewer raises an interesting question. We have Olink data from the same cohort (Sauerwald et al. Cell Systems 2022, published online https://www.cell.com/cell-systems/fulltext/S2405-4712(22)00432-X) although unfortunately the sample overlap with the present study is only 49 subjects. We find that few Olink measurements significantly correlate with our model predictions, and none after FDR correction. Thus, we are not sufficiently powered to draw firm conclusions.

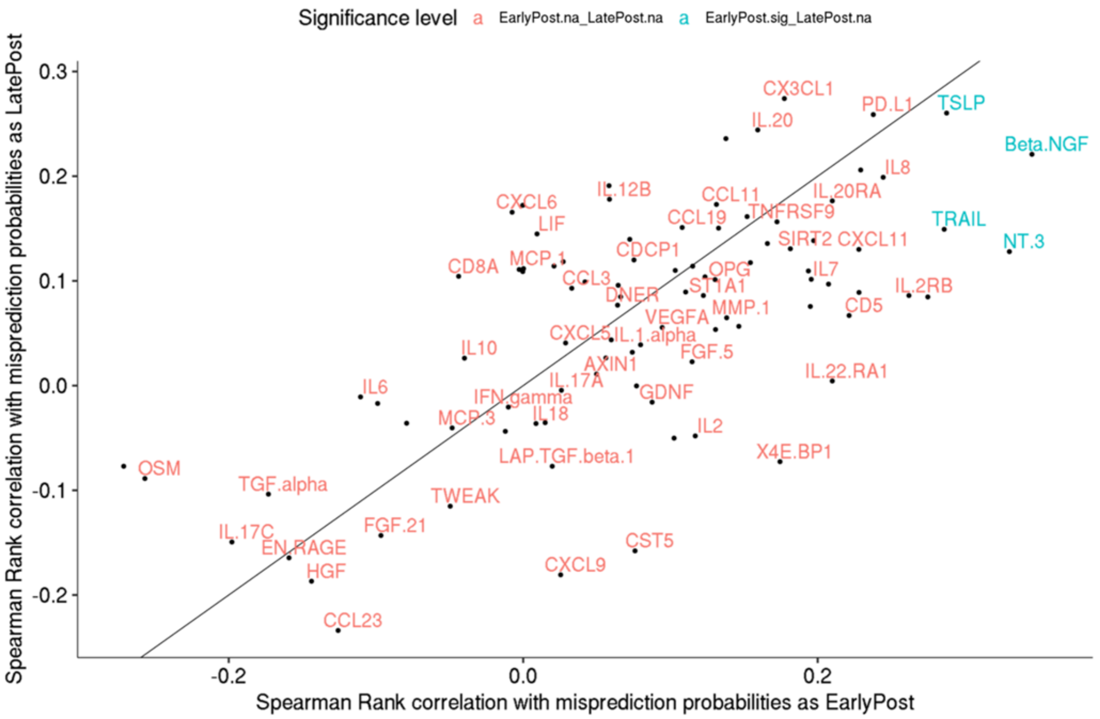

Rank correlation between Olink measurements and model output. Blue indicates significance at $p< 0.05$. No correlations were significant after multiple hypothesis correction.

4. Can the authors speculate whether the susceptibility to other infections than COVID-19 might be influenced by the long-term changes in DNA-methylation.

The early-post methylation pattern is strikingly similar to that seen in SLE. There are claims in the literature that SLE is also associated with an increased infection risk outside of immunosuppressive treatment. However, the data on this point is not definitive. We suspect that the effects observed are not specific to SARS-CoV-2 infection, but prefer not to speculate about this in the manuscript due to the lack of any definitive evidence.

<u>Reviewer #2</u>

In this manuscript, the authors first observed a persistent methylation but not gene expression pattern post asymptomatic or mildly symptomatic SARS-CoV-2 infection by comparing methylation and gene expression profile of patients and control state through the course of the disease in the CHARM study cohort. The prolonged methylation is clustered into seven groups, each is enriched with distinct biological contents. Then, using the methylation data, a regression model was built to predict days post infection. The authors also trained classification models to investigate whether the methylation pattern could be applied to predict other disease state. Finally, the methylation data indicated that a post-infection like methylation profile was anti-protective and associated with poor prognosis regardless of gender. Such methylation seems cumulative, since post-infection like pattern becomes more obvious as age increases. It exerts prolonged effect, and is distinct from previous defined trained immunity. However, despite of intriguing results, there are major problems in article structure and data interpretation.

Major.
1. The major conclusion is not convincing.
(1) ... In other words, the control samples could already be in a post-infection-like state, for example as a result of infection with a different infectious agent or another immune challenge such as vaccination. According to the authors, the post-infection-like methylation pattern has an anti-protective effect (Fig. 5b). Patients who underwent SARS-Cov-2 infection (LatePost) should establish such methylation pattern, and thus should exhibit the same anti-protective effect. However, the probability of misclassifying a control sample as LatePost does not correlate negatively with relative viral levels (Fig EV7b, right). Why?

This comment is related to that of Reviewer 1, point 1. We find a weak but non-significant effect for LatePost on virus load. The trend in the methylation changes suggests that the epigenetic state eventually returns towards baseline, and it would be reasonable to speculate that the effect on future infection may follow the same trajectory. We hypothesize that the effect is indeed strongest in the earlier post-infection phase. See also our answer and additional analyses in the response to Reviewer #1 point 1 above. As described there, we have revised the manuscript to try to more accurately express what can be concluded from our results in terms of the duration of this effect, and to indicate the necessity for further study to more accurately estimate the duration of this anti-protective effect.

(2) The authors assumed that anti-protective methylation was related with innate immunity (... for example as a result of infection with a different infectious agent or another immune challenge such as vaccination.). However, the methylation data is heavily correlated with adaptive immunity (B & T cells, Fig. EV4b). Therefore, the methylation data may reflect the activity of adaptive immunity in each patients. Each control sample may vary in the activity of adaptive immunity intrinsically, and those who resemble EarlyPost state have a more active adaptive immunity preset, resulting low viral loads. If the authors want to conclude that anti-protective methylation comes from previous infection/stimulation, more solid biological data, rather than mathematical manipulation, is needed. Otherwise, it is circular argument.

Regarding the comment about model circularity, we respectfully disagree with this assertion. We clearly show that the model output is predictive of future viral loads and the viral loads were not used in model training. We agree that the biological interpretation is open to discussion

Regarding innate vs. adaptive immunity, we hypothesize that the effect is related to the state of the innate immune system as the subjects were infected with SARS-CoV-2 for the first time and thus should not have any pre-existing adaptive response. Cross reactivity with earlier other coronavirus infections is possible, but seems unlikely given that the pre-infection post-SARS-CoV-2 methylation pattern is anti-protective. We agree that we are not able to determine the cell-type specificity, and cannot exclude the possibility that the state of adaptive cells plays an important role.

In response to this comment, we have now removed the term “innate” from the corresponding section.

2. Inconsistent article structure.
In the part "SARS-CoV-2 methylation clock", the authors trained a regression model to predict days post infection. These contents are disconnected from the remaining parts of the article and thus unnecessary.

We feel that a major finding of our paper is the existence of a quantitative clock that can be used to predict the timing of previous SARS-CoV-2 infection. We cannot refer to this as a methylation clock without this analysis.

3. Improper data interpretation.
In the part "Relationship to other conditions", the classification model is applied in a variety of disease types, such as autoimmune diseases (SLE, multiple sclerosis and rheumatoid arthritis), acute infection (SARS-Cov-2 and influenza) and chronic infection (HCV), which varied greatly in mechanism and phenotype and thus it is not reasonable to generalize the classification model. For example, ... Significant enrichment was observed between EarlyPost period DMS and the HCV study, an HIV study and two SLE studies. How to explain the resemblance? Is the same enrichment observed in other chronic or autoimmune diseases? What are the authors trying to imply with this model? Unless more biological interpretations are made, the generalization is meaningless.

The motivation behind these analyses is to establish the extent to which our observed patterns are specific to infection with SARS-CoV-2, to infection overall, or to conditions in general that modulate immunity. We indeed find that it is the latter.

We have made this motivation more explicit in the text (on p.7, 1st paragraph of the “Relationship to other conditions” subsection:

“In order to characterize the specificity and generalizability of methylation changes observed during SARS-CoV-2 infection we examined whether a model trained to distinguish post PCR+ samples (EarlyPost and LatePost combined) from Control could also distinguish other conditions associated with altered immunological states”

We have also added a concluding sentence at the end of this section (p.8, 2nd paragraph):

“Overall, we find that our methylation model has considerable overlap with other inflammatory conditions including chronic infection and autoimmune diseases and is most similar to SLE. This is consistent with the observation that the changes we observe are related to modulation of interferon signaling, which is activated in SLE.“(Ref: https://lupus.bmj.com/content/6/1/e000270)

Minor.

1. Among the top 16 CpG sites used by the model, we highlight two hypomethylated sites in IFI44L, which were individually inversely correlated with virus level (Fig. 5a): Should be Fig. 5b.

This has been corrected on p. 10, 1st paragraph.

2. Can you define how long the anti-protective methylation pattern exist?
This would require additional study as described in Reviewer 1 point 1 response above. The text has been clarified on this point.

3. Since the authors mentioned train immunity, is methylation involved in the regulation of trained immunity? For example, what is the methylation level of IRF targeted genes?
Methylation has been implicated in trained immunity (see https://www.science.org/doi/full/10.1126/sciadv.abn4002, which reports methylation changes following in vivo and in vitro BCG antigen exposure). Comparing this study with our data we find that the two methylation patterns show no evidence for being related (see Fig. 5d). In the absence of any relationship, we have not proceeded to compare specific targets.

4. Which type of cells play the major role in methylation-related anti-protection?
We are unable to determine the cell of origin for these signals with any confidence. The infection associated probes show less cell-type specificity than a typical probe. Given the relatively small magnitude of the effect, we can hypothesize that the cell type involved is relatively rare and given the duration of the effect that it is long lived, which would rule out most myeloid cells. We hypothesize that the effect is mediated through hematopoietic stem and progenitor cells (HSPCs) in the bone marrow and alters the state of newly generated circulating myeloid cells. This is consistent with results obtained looking at HSPCs in other studies https://www.biorxiv.org/content/10.1101/2022.02.09.479588v1.full

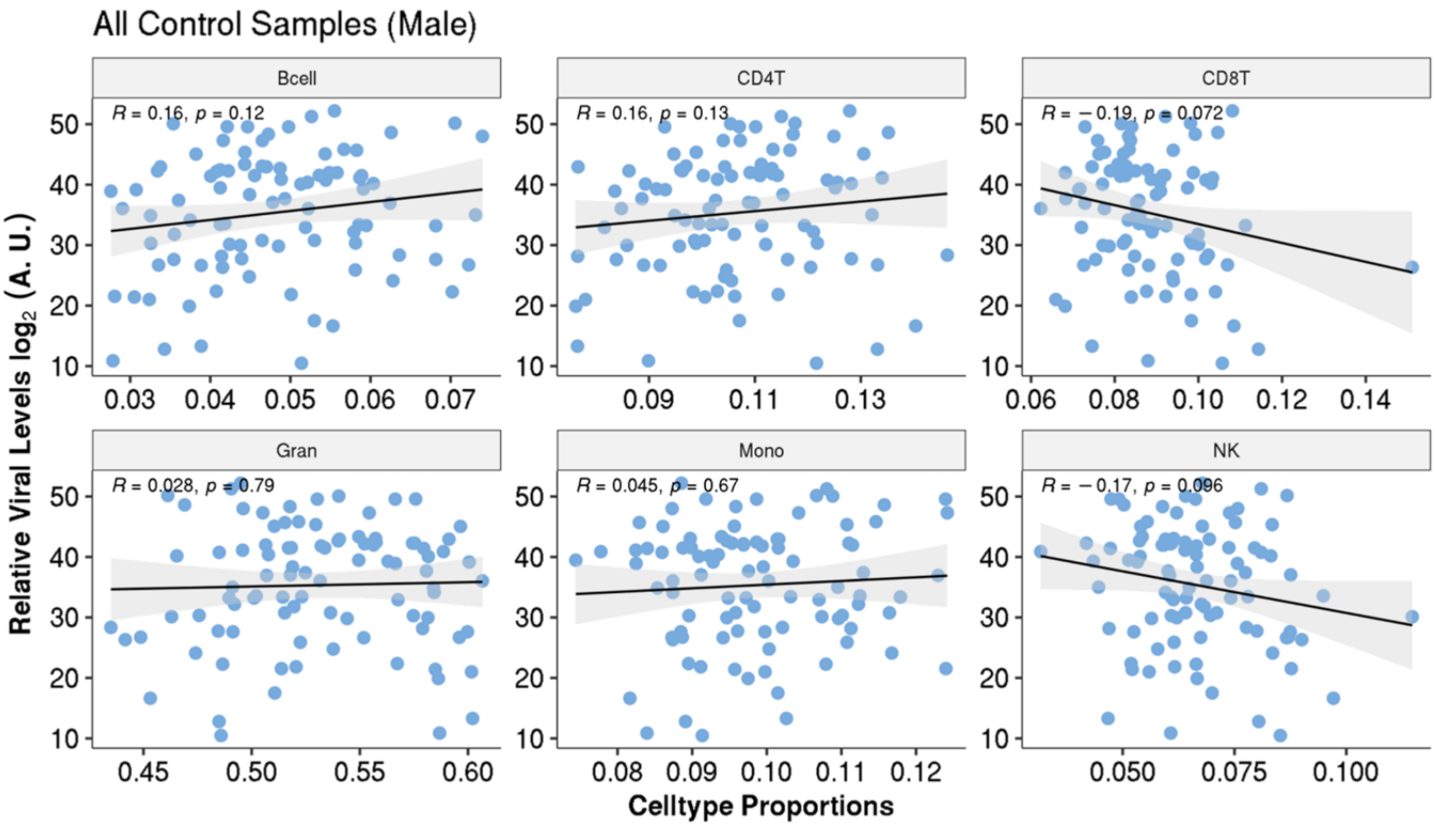

Reviewer #3

The paper "A methylation clock model of mild SARS-CoV-2 infection provides insight into immune dysregulation" from Chikina and Sealfon's labs is a well-conducted longitudinal Covid-infection study of transcriptomic and DNA methylation (DNAm) patterns in a reasonable number of healthy young individuals (all males). The authors find that the severity of symptoms (in this cohort asymptomatic vs mild) did not have an impact on differential expression or DNAm patterns, but that there were differences between pre-infection, first and subsequent PCR+ tests and PCR- post-infection period. In particular, the authors discover that whilst gene expression patterns post-infection largely return to pre-infection levels, that DNAm levels do not. The authors find interesting enrichment of their DNAm signatures for interferon-related genes. The authors use elastic net ML-models to predict both time since infection as well as infection period, and are able to demonstrate the biological meaning of these models by applying it to other Covid-19 cohorts as well as other disease cohorts (mostly autoimmune), finding interesting links with S.Lupus infection, and importantly finding that the level of the post-infection DNAm signature is predictive of Covid-19 severity in an independent cohort.

Overall, I think this is a well-designed and very interesting study, and contributes some important new findings. The figures are great. The authors have also used state-of-the-art statistical methodology throughout. However, there are also some very big concerns, especially with regards to the treatment of cell-type heterogeneity and the interpretation of the DNAm changes, as well as concerns regarding the statistical implementation of their prediction models, that absolutely need addressing:

1) Cell-type correction methodology is very poorly described and appears sub-standard (I): in Methods the authors write "We estimated the proportions of six major cell types (B cells, Granulocytes, Monocytes, NK cells, CD4 T cells and CD8 T cells) using standard reference-based method (Houseman et al, 2012). We took the original CellType450K basis matrix and replaced the values with those from (Roy et al, 2021) (Illumina Methylation microarray). This would help remove the bias induced by the platform inconsistency". As written, this does not make sense and seems to indicate that the authors don't understand how this works. First of all, the Houseman et al paper built a DNAm reference matrix for these cell-types using 450k technology. Approximately 90% of these 450k probes are present on the EPIC array, so in my experience the cell-type fractions estimated using Houseman's procedure using his DNAm reference matrix should work very well on EPIC data too. Indeed DNAm reference matrices can tolerate up to 30% missing values and still perform very well (see e.g. Zheng SC et al Epigenomics 2018). However, if the authors wish to maximize coverage, then you can't just replace values...that is wrong. What you need to do is build a new DNAm reference matrix using the EPIC DNAm data from say Roy et al 2021. But the authors do not describe that they did this, which is disturbing.

We agree with the reviewer that we made several non standard choices for our cell type correction. These choices were made for carefully considered scientific and statistical reasons and with consideration of and experience with all available methods. We clarify the justification for our choices and demonstrate that the conclusions are robust.

Regarding the custom cell-type basis: Using the Houseman basis we noted that we have many 0 values for NK cells, which is not consistent with expectation. While the EPIC array is indeed nearly a superset of the 450K array, there are small distribution shifts in probe values which we hypothesized might cause miscalibration. We thus made a new basis using the exact same probes, but with mean cell-type values from the EPIC platform. This indeed fixed the range issue. However, we emphasize that all the conclusions regarding cell-type associations and cell-type corrected differential analysis are robust to this choice as demonstrated below.

As the reviewer points out, deconvolution is very robust to small changes in the set of informative probes. Therefore we did not see a need to build an entirely new basis matrix from the Roy et al. dataset.

Regarding deconvolution of the RNA data, we have chosen this customized approach in order to generate a comparison across modalities that is as fair as possible. The cell-type estimation problem is fundamentally different for RNA and methylation. Methylation cell type fractions are much more robust and reproducible, but the range of cell-types estimated is more limited. Rare cell types can still be estimated in RNA space if they produce large quantities of cell-type specific transcripts and thus contribute appreciably to the RNA pool. On the other hand the same cell type cannot be estimated using a DNA based assay as the amount of DNA contributed is fixed. A good example of such a cell type is plasmablasts, which can be reliably estimated using RNA but not methylation.

Thus, for our analysis we have chosen to use a single cell-type estimate for both modalities. This ensures that we are correcting for identical cell types across the two modalities and that, in particular, the cell-type corrected model has the same degrees of freedom. We specifically chose the methylation based estimate

as that is more robust in that it doesn't strongly depend on different data processing and algorithmic choices (as is the case with RNA). The reason for training the machine learning model was simply imputation for samples where RNA was available but methylation was not. We have expanded the explanation of our reasoning in the methods section as follows (on p.17, 1st paragraph):

"Our key goal for proportion inference was to ascertain if the major trends in our data such as more prolonged alterations in DNA versus RNA were insensitive to cell proportion correction. As proportion estimation from RNA and methylation differs greatly in terms of robustness and the number of cell types which can be estimated (methylation is more robust while RNA can be used to estimate some rare cell types) in order to formulate a fair comparison we correct both modalities for the same cell proportion estimates. We used the methylation estimated proportions as a gold standard. For RNA samples with no matching methylation, the proportions were imputed using a simple machine learning model. "

In response to the reviewer's comment, we have repeated the analysis of both cell-type changes and cell-type corrected differential signals using a variety of different deconvolution approaches. We find that the main conclusions that:

1. Cell type correction has a limited effect on the differential signal (See discussion of this below)
2. Methylation changes are more prolonged than gene expression changes

are very robust to the choice of cell type estimation method. To illustrate this, we have reproduced Figure 1b which shows differential expression using a variety of different cell type inference algorithms including CIBERSORTx and the recent expanded 12 cell-type methylation basis from Salas et al. We now provide this figure in Appendix Figure S5, and have revised the text to refer to the robustness of these results (on p. 4, 2nd paragraph).

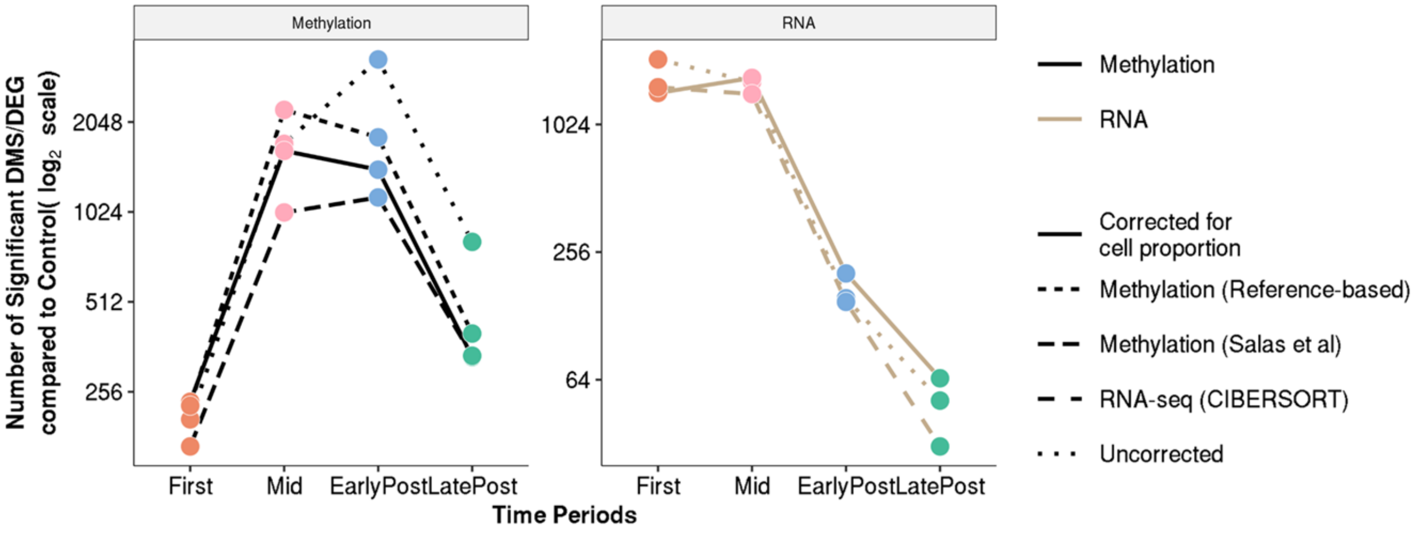

We also show below the differential type cell-analysis using the same estimates. The methods show consistent trends with the greatest changes across pseudotime groups seen in NK cells. See figures below. These results are now included in the manuscript as Appendix Figure S6 (on p. 4, 2nd paragraph).

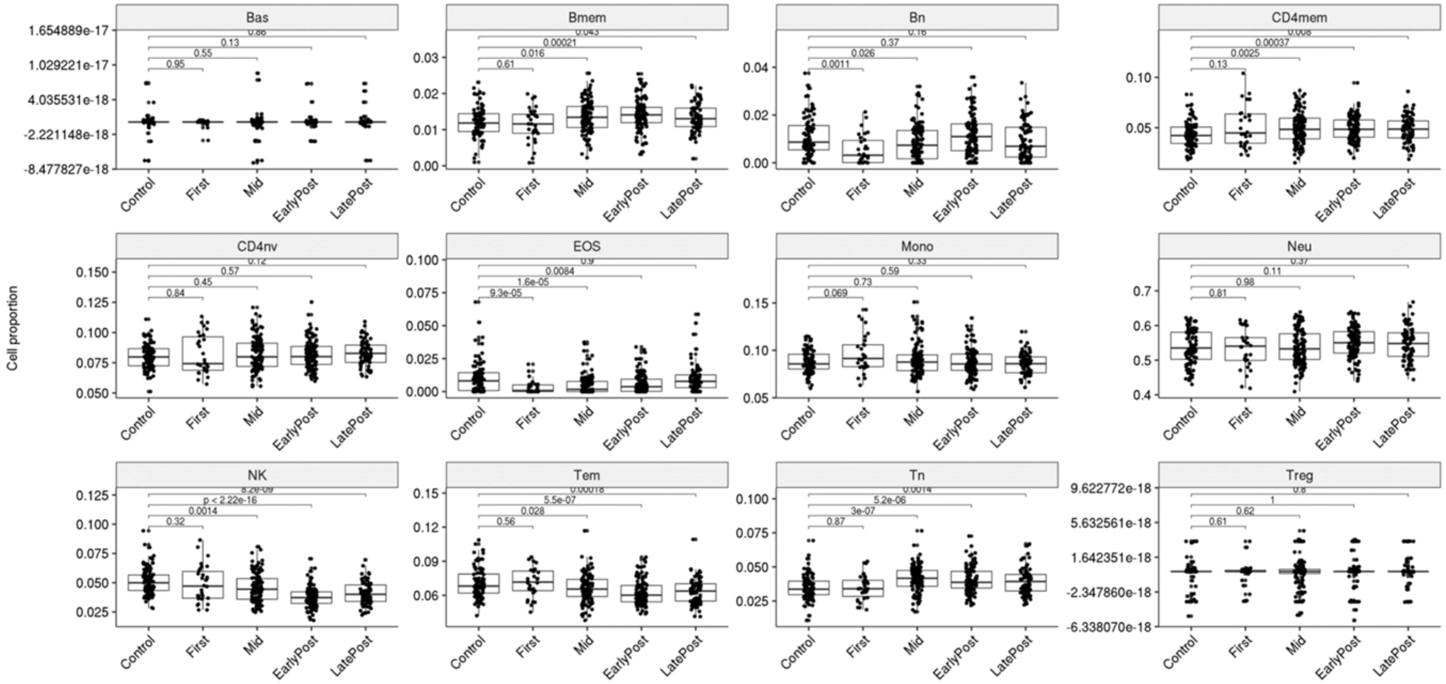

Figure above: Celltype estimation based on the methylation data using an enhanced basis (EPIC) from Salas et al.

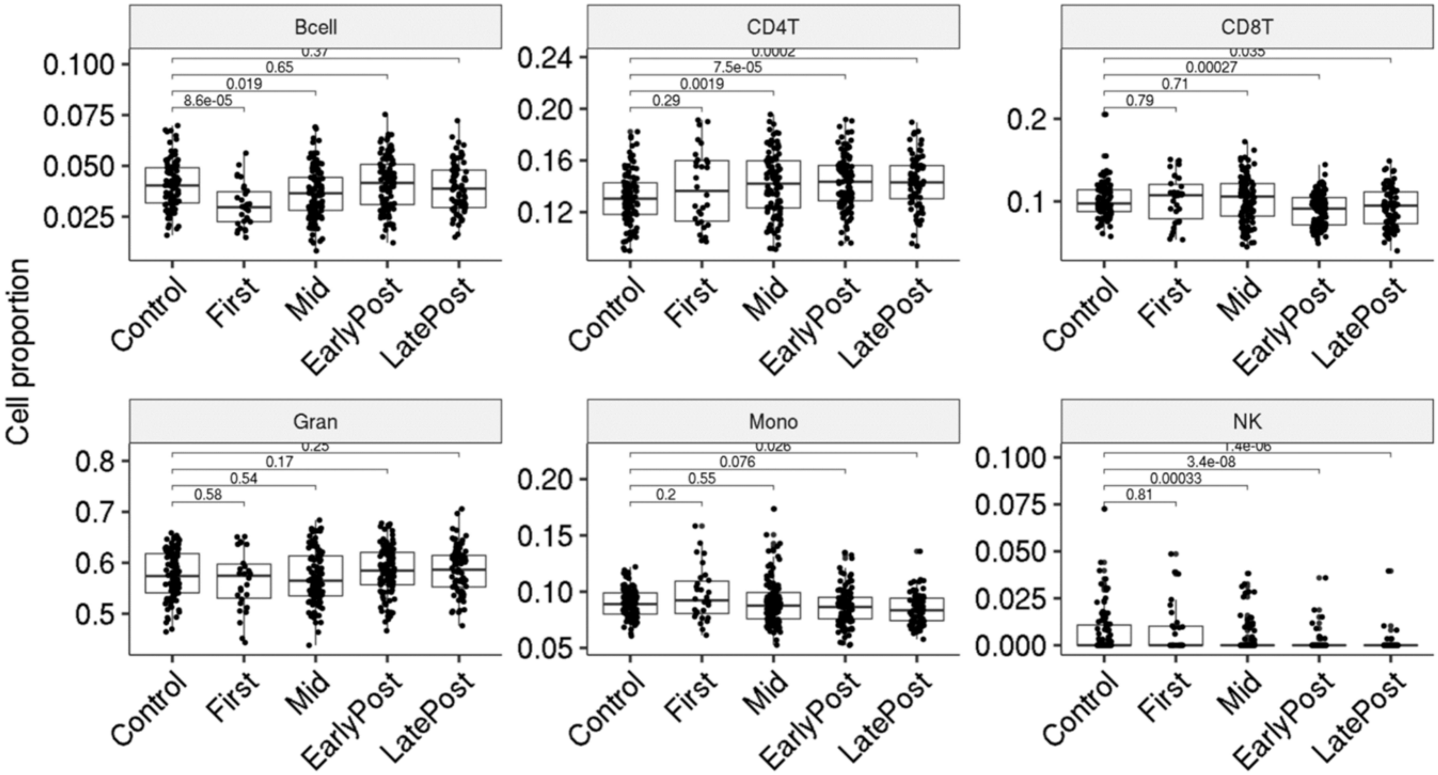

Figure above: Celltype estimation based on the methylation data using basis from Houseman et al. (ref-based)

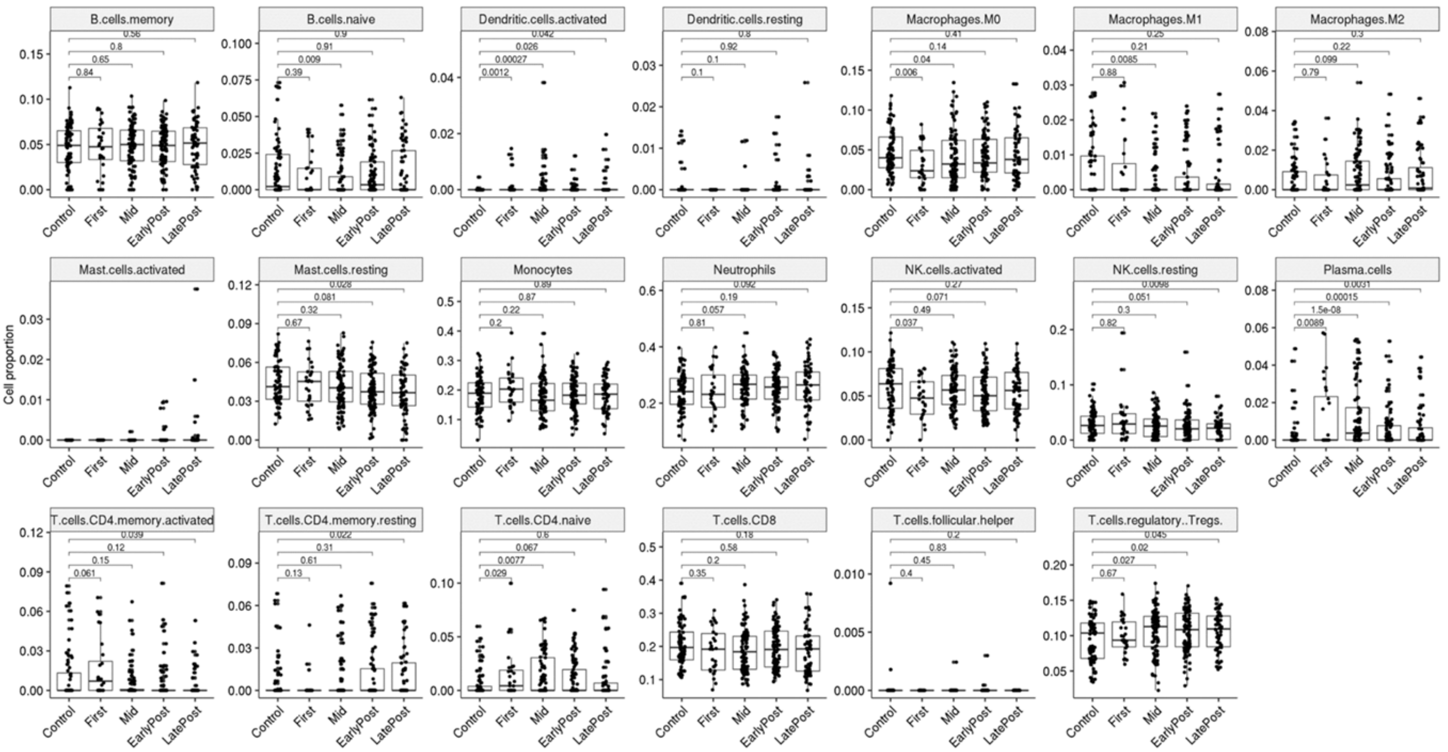

Figure above: Celltype estimation based on CIBERSORTx. We note that CIBERSORTx includes interferon response markers in their basis set, which does cause some differences in deconvolution of virus infection time course data (e.g. the increase in activated dendritic cell proportion seen at Mid, which is not consistent with other methods).

2) Cell-type correction methodology is very poorly described and appears sub-standard (II): In the same paragraph there is another problematic issue, which is that the authors adjust for cell-type heterogeneity by regressing M-values to the estimated cell-type fractions, and then seemingly transform back to beta-values. Irrespective of whether this procedure has been borrowed from another paper (in which case that paper should be revised...), it is wrong. M-values are unbounded, but fractions are bounded between 0 and 1. The methylation beta-value you measure is a linear sum of cell-type fractions times the methylation beta-values in the specific cell-types. So, the cell-type fractions appears naturally in the beta-value basis and therefore adjusting for cell-type fractions should be done in the beta-value basis. The residuals of course are not beta-valued but they also don't need to be. You can still use the residual matrix downstream. It would be incorrect for the authors to assume that they can have "a cell-type fraction adjusted data matrix" in the beta-valued basis. In theory what you actually want is the data matrix over CpGs and samples for each individual cell-type but this is an extremely hard problem and current methods don't work well.

We have expanded both our analyses and the description of our cell-type correction method as detailed in our response to the previous questions. Regarding, M-values vs. betas, we only used M-values for the batch correction step which uses ComBat. We reasoned that since the model assumes a Gaussian distribution M-values were more appropriate. As stated in the text (on p.16, 1st paragraph):

“ We applied ComBat (Johnson et al, 2007) in the M-value space to regress out potential technical covariates including Array (EPIC array), Slide (EPIC array) and batches (EPIC array plates). Then we converted methylation levels of 707,361 CpG sites from M-values to beta-values for all the downstream analysis.”

The result was then transformed back into beta space and beta values were used for all subsequent analysis including cell-type estimates. We also note that as stated in the text the final limma differential expression is performed on z-score transformed beta-values as we found that many probes with similar trajectories had widely variable ranges. This being a linear transformation, it does not affect T-tests.

3) Celltype fractions as covariates: the authors should also make it clearer when they are using the residuals, and when they are using them as covariates in their limma model. It should be clear that you don't need to do both together, that would be wrong. It is advised to use them as covariates in the linear models (limma) whenever possible.

We did include cell-types as covariates in the linear regression model. This is stated in the Methods section as follows (on p.18, 1st paragraph):

“When cell type proportions were corrected, the proportions of six major cell types (Monocyte%, Bcell%, Gran%, CD4T%, CD8T%, NK%) were also included as biological covariates.”

4) Variations in celltype fractions and interpretation: I am a little puzzled regarding the interpretation of the derived DNAm signatures (Fig.2a) and their insensitivity to cell-type adjustment. On the one hand, Fig.EV2, clearly shows changes in cell-type proportions with infection period. For instance, B-cells and NK-cells display quite big changes. And yet, adjustment for cell-type fractions does not seem to alter the observed DNAm signatures? What about the enrichment of TFBSs in Fig.2b? Does that not point to specific cell-types and hence that these signatures are driven by corresponding changes in cell-type proportions? In other words, the authors don't seem to interpret any of their signatures in terms of variations in cell-type fractions, and frankly I find that hard to believe. It could be down to the fact that the authors have not adjusted for cell-type fractions in the correct manner (see previous points).

We agree that we cannot exclude the possibility that the changes we observe are indeed differences in cell-types that we have not adjusted for. As we consider fine grained cell-type distinctions the difference between cell-type and gene regulation becomes difficult to delineate. What we can confidently say is that the top differentially methylated probes are not explained by changes in major cell-types. Indeed, our differential probes show relatively little cell-type specificity as judged by the cell-type coefficients in the model used for differential testing.

With regard to how the number of DMCs changes when cell type correction is applied, generally cell-type correction can both decrease and increase discoveries. We also note that while the changes in cell proportions we observe are indeed highly significant, in some cases they are still small in absolute magnitude.

We have included additional text to clarify the limitations of our cell-type correction (on p. 4, 2nd paragraph).

“These conclusions were robust to changes in the computational framework used to infer cell proportions (Appendix Figure S5 and Appendix Figure S6). We cannot exclude the possibility that some of the differences we observe correspond to changes in the frequency of a cell-type that is not accounted for in computational cell type deconvolution methods.”

5) Estimation of celltype fractions with RNAseq: I don't understand what the authors did here. Frankly, by now there are many cell-type fraction estimation tools available for RNA-Seq data, including CIBERSORT. There are corresponding mRNA expression reference matrices. Just use that. Why train a model using paired DNAm data? What is the purpose of this? The authors are not pushing boundaries here in terms of deriving a new mRNA expression reference matrix for blood, so what is the point of this? The good agreement in Fig.EV2 between mRNA and DNAm is NOT impressive at all, if the mRNA-based estimates were trained using DNAm data. I find Fig.EV2a-b comparison to be very misleading in fact. Please change your mRNA-based method so that it only uses mRNA data!

We believe that our additional explanation and analyses performed for comment 1 apply to this comment as well. In particular, we demonstrate that our conclusions are robust to using CIBERSORTx.

6) Dynamic time warping: this is a nice method, but neither in Results nor Methods, do the authors explain what it actually does. Please add 1-2 sentences explaining what it actually does and why it was used in Fig.2a. We want authors to apply methods because they understand them, otherwise it gives the impression they are just applying a black-box. And please cite the corresponding paper in main text, not just in Methods.

We add the following text to the main text (on p. 18 in the “7. Temporal clustering (Figure 2a)” subsection:

Dynamic time warping is an algorithm that calculates the optimal matching between two time series ([1,2]). It measures similarity based on overall trajectory, regardless of speed. These characteristics make it beneficial for clustering differential features according to their temporal trajectory patterns.

1. Liu, Xueli, and Hans-Georg Müller. "Modes and clustering for time-warped gene expression profile data." *Bioinformatics* 19.15 (2003): 1937-1944.
2. Leng, Xiaoyan, and Hans-Georg Müller. "Classification using functional data analysis for temporal gene expression data." *Bioinformatics* 22.1 (2006): 68-76.

7) Prediction models on external datasets: The authors use a rather unconventional method to build the models that are applied to the external datasets. If the authors used a nested CV on their training-test set splits, then why not run the internal CV loop on the full dataset to derive a prediction model that can then be applied to the external datasets? Or alternatively, because the authors have derived 100 classification/prediction models, one for each training-test set split, they could apply an ensemble approach where each of the 100 are tested on the external dataset. Prediction/classification scores can then be averaged to arrive a final prediction/classification

score for each sample in the external dataset. These last two approaches are more conventional and rigorous than the ad-hoc approach of the authors.

The reason we had chosen our approach to model building is that we were interested in applying the model to external datasets, most of which are on the 450K array unlike our data which is from the 850K array. As there are small but important domain shifts between the two platforms, we reasoned that selecting the most robust probes would improve model transfer. However, the hospital outcome dataset in Figure 5C contains the 850K EPIC, and we followed the reviewers suggestions for this dataset. We find that the results show the same trends, and the p-values were more significant when using the ensemble model. The reasoning for our model choices is now more explicit in the Methods (on p.21, 2nd paragraph).

"In order to build a general model that is applicable for external datasets, we first selected features that were robust (frequently selected over all outer train-test splits) and then built the model only with these most stable features. This procedure makes the model more robust to domain shifts that arise when switching from the 850K EPIC methylation arrays (our data) to the 450K platform (nearly all external datasets). "

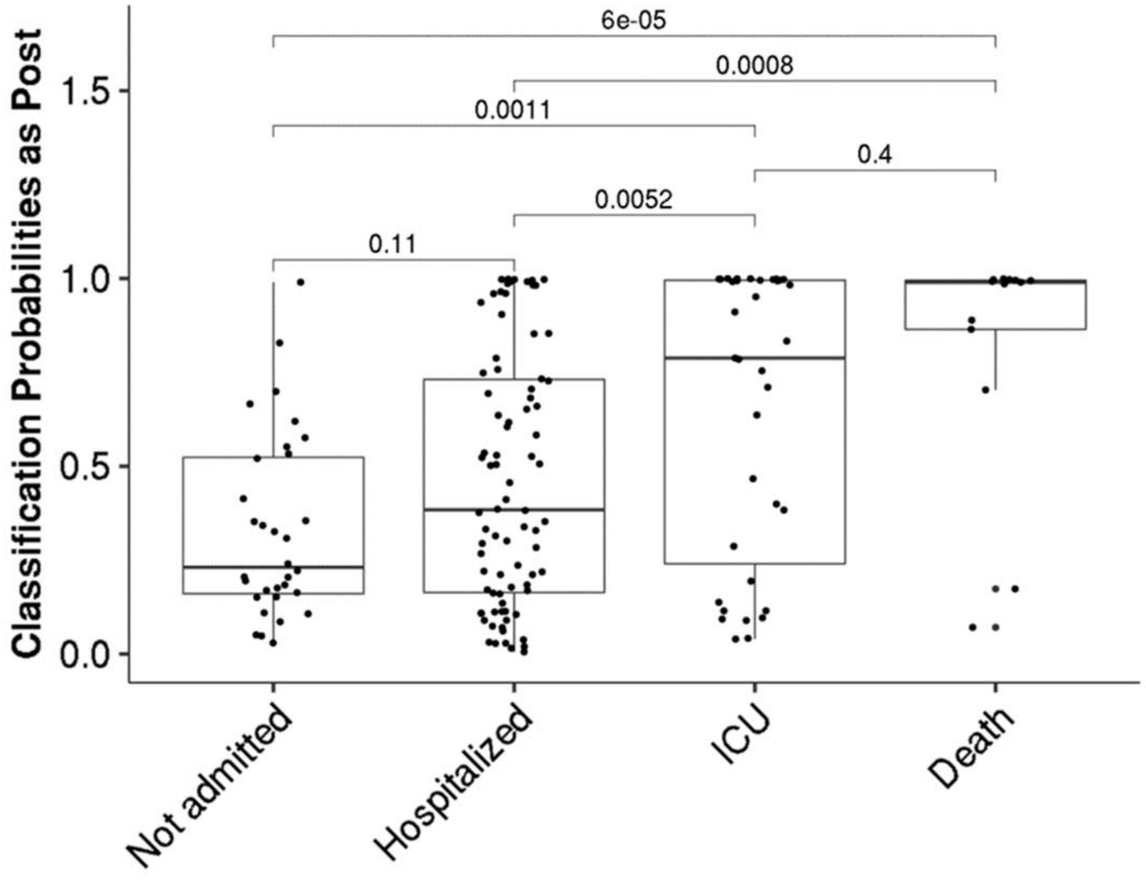

Ensemble model applied to age regressed methylation data.

8) Fig.1c-d: can the authors please confirm that in Fig.1c they have not removed any datapoints from the other two quadrants? I just found the agreement between asymptomatic and symptomatic to be amazingly good. As far as Fig.1d is concerned, I did not find this PCA panel to convey any useful message. I would remove it.

While there is no specific constraint on which quadrant will contain points, the probes plotted had to be differentially methylated overall and differentially methylated in either symptomatic or asymptomatic groups. This analysis was performed as follows. We repeated the differential analysis separately for symptomatic subjects and asymptomatic subjects. For each differential contrast, we first selected DMS in Fig. 1b (all subjects) that were also differentially methylated (FDR < 0.05) within symptomatic or asymptomatic groups. Then we extracted the corresponding differential statistics and generated the scatter plot to demonstrate that the DMS were indistinguishable with respect to the symptoms.

In response to the reviewer's comment, we have modified the legend to Fig. 1c to include the statement (on p.29):

"For each differential contrast, we first selected DMS in Fig. 1b (all subjects) that were also differentially methylated (FDR < 0.05) within symptomatic or asymptomatic groups."

With respect to Fig. 1d, we feel that many readers will find the overall progression of DEG and DMS through PC space to be a helpful high level summary of the data. We thank the reviewer for noting that the point we are making with this plot may not be evident. We have revised the legend to include the statement (on p.29):

We note that for gene expression Post time points are very close to Control while this is not the case for methylation. Moreover, the pattern is similar for DEG associated and other differential probes.

9) External datasets (Fig.EV6): On first mention in the main text, there are no details provided regarding these external datasets. What are they? And in Fig.EV6 I missed 95% confidence intervals for the AUCs specially in panel-A where the number of samples is quite low.

We have added the confidence intervals as requested (revised Fig. EV6a, now Appendix Figure S2a, reproduced below). These are not external datasets, but simply model performance on our study samples separated by subject sex. We have amended the text to clarify this as follows (on p.6-7, in the "SARS-CoV-2 methylation clock" subsection):

To determine whether these analyses were applicable to females, we examined the classification of the male and female participants in our dataset separately. We compared the multiclass classifier performance in the 31 samples from the 11 female participants (Fig. EV6a) and in the 397 samples from the 122 male participants (Fig. EV6b). Overall, the samples from both sexes were classified with similar accuracy, although the confidence intervals for females were wide due to small sample size. "

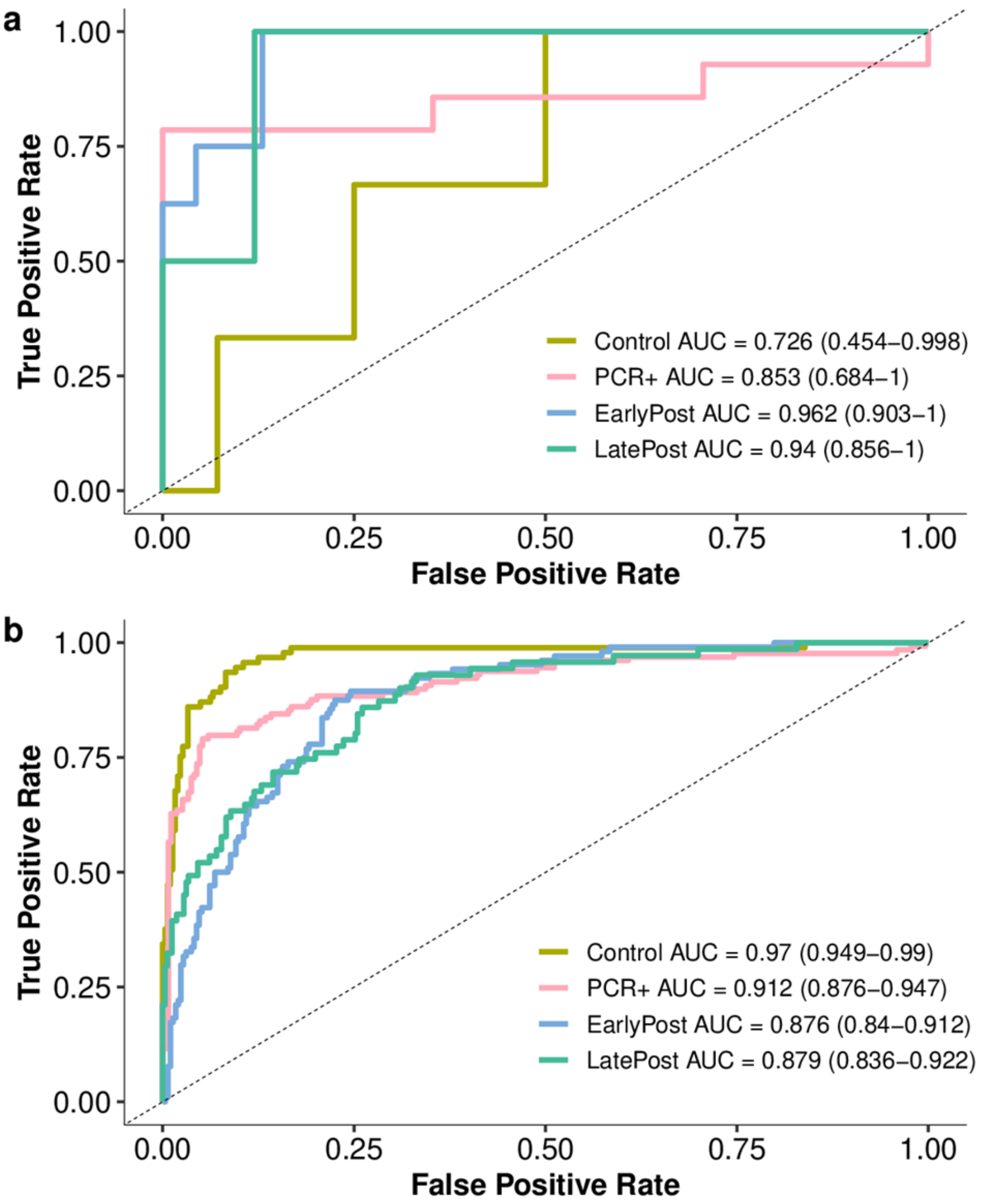

10) Fig.5b: the data displayed in this panel is not very convincing. The P-values are also quite marginal and some experts have been arguing that significance thresholds should be lowered to <0.001 e.g. to account for multiple-testing of "tests". Moreover, the x-axis which labels the probability of misprediction only varies to 0.3?? I am surprised by that. I thought some samples would have a probability larger than 0.5, or why then are they "misclassified"?

We agree with the reviewer that a stronger significance level would be preferable, which could presumably be achieved using a larger sample size. However, this is a very unique study and adding samples is not feasible. In this light we feel that a p-value of .0012 is reasonably close to the threshold the reviewer would prefer. Regarding the misprediction probability, we are indeed using the term loosely. We

are quantifying variation within the not yet infected Control group, which on average is predicted correctly since the model was trained on these samples. We appreciate that the reviewer has pointed out that our presentation here is confusing. In order to avoid this confusion, we have added the following text when we first introduced this misclassification analysis (on p.9, 2nd paragraph):

We note that nearly all samples are, in fact, correctly classified by the model. We use the term “misclassification” here to reflect merely the quantitative probability obtained from the model of classifying the samples as belonging to the wrong class.

11) Fig.3a: I am also very troubled by Fig.3a, because the x-axis values go up to 80 days after diagnosis, but the predicted times only go up to 40. So, if instead of using a correlation we were to use RMSE, we would have an extremely large error. I wonder if this large RMSE reflects an error or problem in the training of the regression model? Seems like it. Why not train the model to minimize the RMSE?

The models are in fact trained with MSE loss and not correlation, but we show correlation as that is the readily interpretable unitless quantity. We have revised the legend of Fig. 3a to include the statement (on p. 30):

“The results shown were trained with mean squared error, but are depicted as a correlation plot to facilitate interpretation.”

We note that given the variance in the output variable Y it is straightforward to translate MSE to correlation as the two values are related by a linear transformation. We have very few data points at 80 so they contribute very little to the loss which explains why the best fit (minimum MSE loss) model does not extend to the full data range.

Minor point:
It is great to see that the authors used the correct nested CV strategy in their model training. It would be good if the authors could cite a few papers that have emphasized the importance of nested CV: Simon, R., et al J. Natl Cancer Inst. 95, 14-18 (2003) and Teschendorff et al Nat Materials 2019.

We have now included the following citations (on p. 21, 1st paragraph).

1. Simon, Richard, et al. "Pitfalls in the use of DNA microarray data for diagnostic and prognostic classification." *Journal of the National Cancer Institute* 95.1 (2003): 14-18.
2. Teschendorff, Andrew E. "Avoiding common pitfalls in machine learning omic data science." *Nature materials* 18.5 (2019): 422-427.

Thank you for sending us your revised manuscript. We have now heard back from the two referees who agreed to evaluate your study. From the comments below, you will see while Reviewer #2 indicated that they were satisfied with the revisions, Reviewer #3 still raised significant concerns about the data analysis. Regarding these remaining issues, we have additionally consulted with an expert in the field and shared your preliminary point-by-point response with them. As you will see below, the advisor made constructive suggestions, and we would ask you to address the remaining concerns of Reviewer #3 in a revised version of the manuscript according to the external advisor's recommendations.

Reviewer #2:

The authors have responded well to my comments.

Reviewer #3:

The revised version of the paper "A methylation clock model of mild SARS-CoV-2 infection provides insight into immune dysregulation" has improved. However, a number of major concerns still remain:
1) The authors did not address my concern about their approach to regress out cell-type fractions in the M-value basis. In fact, in Methods the authors still state "We further regressed out cell type proportions by linear regression in the M-value space and created the cell-type corrected beta value matrix for some downstream analysis". I tried to explain to the authors that statistically speaking this is problematic because the natural exact relationship that exists between beta-values and cell-type proportions (a beta-value in tissue can be expressed exactly as a linear sum of cell-type proportions and beta-values in the actual cell-types) is "destroyed" when you transform beta-values to M-values. Hence, the "regressing out" of cell-type proportions in the M-value space leads to a biased residual matrix. In any case, the author's statement in Methods plainly contradicts their responses provided in their rebuttal letter, because in the rebuttal they categorically stated that they only used M-values for batch correction. In fact, as written in Methods, one gets the impression that the authors have regressed out cell-type fractions in the M-value basis and then corrected for cell-type fractions again when performing DMS-type analyses. This again is poor scholarship, because you don't need and you should never adjust for the same factors twice. I understand that how exactly you perform these analysis may not greatly affect results on this particular dataset, yet this is not a valid reason for not doing things properly. On other datasets, the specifics of the method could greatly impact results. The important thing here is to retain a certain level of scholarship.

2) A similar comment applies to the author's refusal to regenerate a new EPIC DNAm reference matrix for blood from the Roy et al data. Whilst I agree that replacing the values in the older 450k DNAm reference matrix with EPIC-derived ones, should work, it again reflects poor scientific reasoning and scholarship. That the authors express so much concern about differences in 450k and EPIC arrays, raises the question why they don't derive a new DNAm reference matrix from Roy et al data, which is EPIC, thus circumventing their original concern. Indeed, given that not all 450k probes are on EPIC arrays, isn't the loss of probes a bigger concern than very small shifts in the mean DNAm values between 450k and EPIC? It seems to me that the authors are just cherry-picking their concerns, whilst ignoring others. Again, whilst I agree that all of this will not greatly affect downstream analyses in this particular study, it is generally speaking poor scholarship to replace the entries of a 450k DNAm reference matrix with values from an EPIC array because those values also derive from *different sets of samples*, so you are not altering the values simply by a technical difference but also by a biological difference (e.g. different ages).
3) Finally, I was also not persuaded by the author's argument in relation to Fig.3a. For the task of predicting time since infection, I would strongly argue that it is the absolute error estimate that matters. A Pearson correlation is not applicable to ONE new sample for which you need to predict the time from infection. However, the difference between predicted time and actual time is applicable to ONE new sample. Hence, based on Fig.3a, I find it very troubling that the MSE is quite large, for instance, even for samples at 30/40 days, we can see that the error is a whopping 10 days. How is such a large error applicable in clinical practice? I honestly don't see how this advances the field.

External advisor:

Rev 3 point 1
A large body of literature of modelling cell-type proportions in DNA methylation are performed in the beta-value space (e.g. https://bmcbioinformatics.biomedcentral.com/articles/10.1186/s12859-017-1511-5,https://bmcbioinformatics.biomedcentral.com/articles/10.1186/1471-2105-13-86,https://bmcbioinformatics.biomedcentral.com/articles/10.1186/s12859-016-1140-4, https://www.nature.com/articles/s41467-019-11052-9,https://genomebiology.biomedcentral.com/articles/10.1186/s13059-017-1182-6, https://www.nature.com/articles/nmeth.3809). This supports the general stance of the reviewer that cell-type modelling of DNA methylation data is performed in the beta-value space. Many of these studies make use of methylation array data, where M-value are easily accessible, but were not chosen for modelling cell type mixtures, despite it being clear that differential methylation calling should be performed in the M-value space (especially when using models assuming normality). This is very likely because the additivity of cell-type specific DNA methylation in the beta-value space is much easier to model. So, even if

the authors show that there modelling of cell-type proportions in the M-value space (in their B2M2B model) is similar to the modelling in the beta-value space (B_regress), the question remains why the authors prefer to show the B2M2B results instead of the B_regress results given that the majority of the cell-type deconvolution has been done in the beta-value space. My advice would that the authors redo figure 2A based on the B_regress model (which should not affect the final results in a major way), and state that performed the correction in the beta-value space as this is what is commonly done, and for a later study present results on the B2M2B vs the B_regress approaches to start a discussion of the whether cell-type modelling should be performed in the M-value vs beta-value space (as the comparison is not the major focus of the study).

Rev 3 Point 2
With regards to the suitability of replacing 450k cell-type specific CpGs with EPIC values, I believe that both the reviewer and authors agree that this will have minimal effect. However, in order to satisfy the reviewer concern that while what the authors did sounds very reasonable, they should show some evidence that it should. This could be in the form of citing a previous publication where a similar approach was done, or performing analysis similar to ANOVA to show that indeed more than X% of CpGs are different between groups, or create a scatter plot of the 450k values against the EPIC values and report the correlation, or simply create a heatmap similar to Roy et al Fig 1e to show that visual cell-type specificity is maintained. I do not think this would constitute a major amount of work and should reasonably address the reviewers minor concern.

External Advisor (Reviewer #4)

Comment #1

A large body of literature of modelling cell-type proportions in DNA methylation are performed in the beta-value space (e.g. https://bmcbioinformatics.biomedcentral.com/articles/10.1186/s12859-017-1511-5,https://bmcbioinformatics.biomedcentral.com/articles/10.1186/1471-2105-13-86,https://bmcbioinformatics.biomedcentral.com/articles/10.1186/s12859-016-1140-4, https://www.nature.com/articles/s41467-019-11052-9,https://genomebiology.biomedcentral.com/articles/10.1186/s13059-017-1182-6, https://www.nature.com/articles/nmeth.3809). This supports the general stance of the reviewer that cell-type modelling of DNA methylation data is performed in the beta-value space. Many of these studies make use of methylation array data, where M-value are easily accessible, but were not chosen for modelling cell type mixtures, despite it being clear that differential methylation calling should be performed in the M-value space (especially when using models assuming normality). This is very likely because the additivity of cell-type specific DNA methylation in the beta-value space is much easier to model. So, even if the authors show that there modelling of cell-type proportions in the M-value space (in their B2M2B model) is similar to the modelling in the beta-value space (B_regress), the question remains why the authors prefer to show the B2M2B results instead of the B_regress results given that the majority of the cell-type deconvolution has been done in the beta-value space. My advice would that the authors redo figure 2A based on the B_regress model (which should not affect the final results in a major way), and state that performed the correction in the beta-value space as this is what is commonly done, and for a later study present results on the B2M2B vs the B_regress approaches to start a discussion of the whether cell-type modelling should be performed in the M-value vs beta-value space (as the comparison is not the major focus of the study).

Authors' Response: The goal of the analysis shown in Fig. 2 in the manuscript is to achieve the best clustering possible for summarization of the patterns of response and for supporting biological interpretation. We have performed the B_regress analysis as suggested by the reviewer. Although the re-analysis does not change the conclusions of the manuscript, we find that the clustering obtained with our original B2M2B analysis is objectively better than that obtained with a B_regress analysis. We judge the quality of the analysis in two ways: 1) are the clusters better distinguished and 2) are the clusters more biologically coherent (i.e. does annotation of the clusters--for example TF binding sites--show better or worse discrimination among the clusters). For point #1, we determined total within-cluster variances (SSE), shown in the table below. Smaller values indicate a better fit. As can be seen from the table below, the B2M2B clustering achieves a smaller value. This may seem somewhat counterintuitive, but clustering solves an NP-hard combinatorial problem with a greedy heuristic algorithm. Our results suggest that the

application of this greedy algorithm to B2M2B provides a better solution to the global optimization problem.   For point #2, if we look at the TF annotation of each cluster in the original B2M2B analysis vs. the B_regress analysis, the B_regress analysis fails to identify any unique TFs significantly associated with Clusters 2, 6 and 7, whereas annotation of the original B2M2B analysis identifies unique TFs for all 7 clusters. Furthermore there were only 45 unique TFs with the B_regress analysis and 61 with the B2M2B. These findings indicate that the B2M2B analysis is better because the clusters obtained are more distinct and show TF annotation results that distinguish each cluster (Figs. 1a and 2a below).

We appreciate that the B_regress analysis is more common and that some readers (and the reviewers) would like to know what the results were with B_regress analysis. However, while it does not alter the conclusions of the manuscript, we believe the clustering and annotation results using B_regress analysis are inferior. Therefore, we propose to leave the B2M2B analysis in the main figure and include the B_regress analysis and annotation as a supplementary figure. The methods have been revised to explain the rationale for the B2M2B analysis and to cite the alternative results.

Normalization version	SSE
B2M2B (current)	**29850**
B_regress	30730

Table 1. In order to evaluate the clustering obtained using the B2M2B method (corresponding to the original analysis in Fig. 2 of the manuscript) and the B_regress method requested by the reviewers, we determined total within-cluster variance (SSE) for each approach. We utilized the standard implementation of hierarchical clustering embedded in R the with ward.D2 option to minimize the total within-cluster variance. We calculated the dynamic time warping distance as the pairwise distance for any two DMS. We find that the clustering obtained from the original analysis is objectively tighter than the clustering obtained using the B_regress approach.

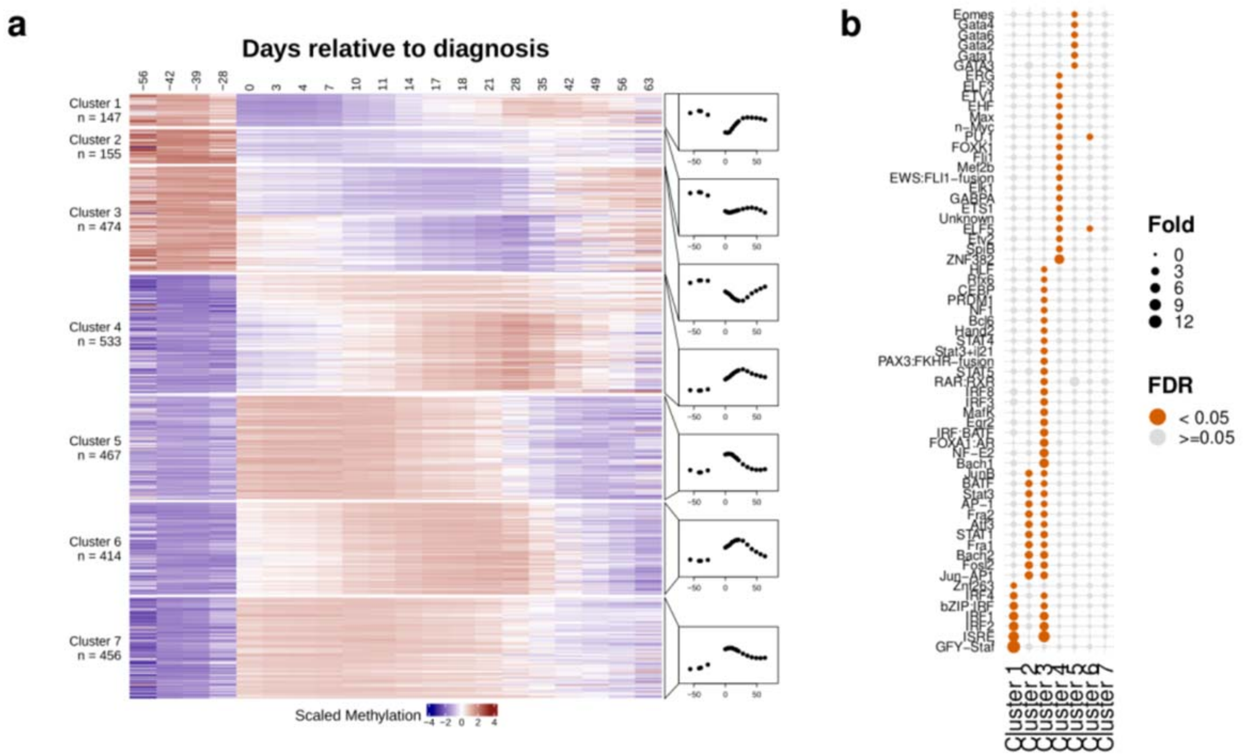

Figure 1. This is a remake of Figure 2 in the manuscript using the B_regress analysis requested by the reviewer. **a**, Z-scored levels at DMS clustered by temporal trajectory relative to the first PCR-positive test. Plotted is the average of each cluster over time. **b**, Enrichment of TFBS by cluster within a 200-bp window centered at each DMS, FDR <.05 for at least one cluster. Fold, fold enrichment. Unlike the results obtained with the B2M2B analysis shown in Fig. 2 in the original version and reproduced below, the TF enrichment indicates that the results with B_regress are clusters that have less biological coherence. Using B_regress, as shown above, there is less distinction of the TFs between clusters and several of the clusters in the TF enrichment lack any unique annotation at all.

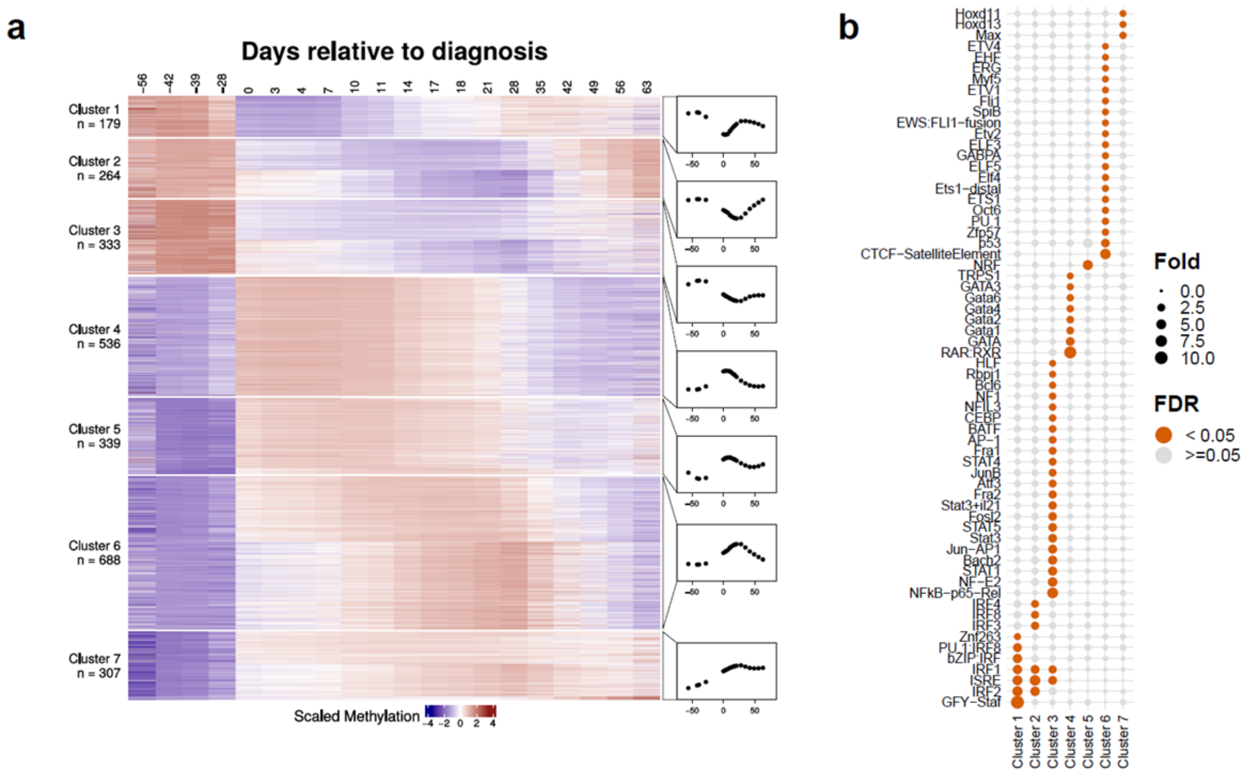

Figure 2. For comparison to Fig. 1 above, we reproduce the Fig. 2 from the original manuscript, which includes the B2M2B clustering and TF annotation.

Comment #2:
With regards to the suitability of replacing 450k cell-type specific CpGs with EPIC values, I believe that both the reviewer and authors agree that this will have minimal effect. However, in order to satisfy the reviewer concern that while what the authors did sounds very reasonable, they should show some evidence that it should. This could be in the form of citing a previous publication where a similar approach was done, or performing analysis similar to ANOVA to show that indeed more than X% of CpGs are different between groups, or create a scatter plot of the 450k values against the EPIC values and report the correlation, or simply create a heatmap similar to Roy et al Fig 1e to show that visual cell-type specificity is maintained. I do not think this would constitute a major amount of work and should reasonably address the reviewers minor concern.

Authors' Response: Roy et al Fig 1e (Fig. 3 below) is not a heatmap of a basis matrix. In particular it does not differentiate CD4 and CD8 T cells and also contains no cell-type specific hypermethylated probes (which are valuable for deconvolution). As such, a direct comparison between our analysis and that shown in Roy et al. Fig. 1e is difficult to interpret. In keeping with the spirit of the reviewer's request, we compare the basis we used to the standard Houseman et al. basis for the 450K array (Houseman et al., 2012; Fig. 4 below). It is clear that the cell-type specificity blocks are preserved and in some cases actually improved. In particular, we find that the hypomethylated values are generally lower in the new basis. In addition, as suggested by

the reviewer, we also show a plot of the correlation of the 450K values against the EPIC array (below) which shows nearly perfect correlation.

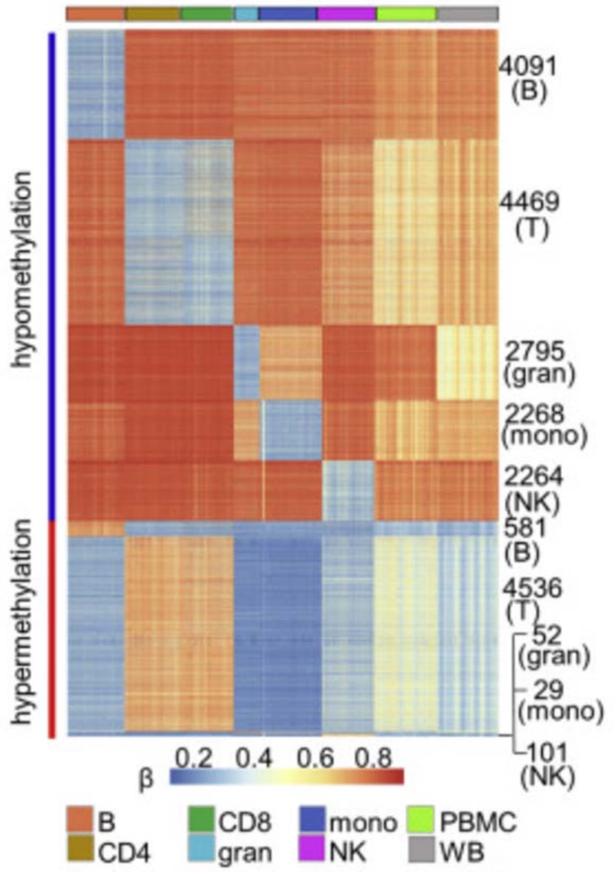

Fig 3. Fig 1e from Roy et al. [1]

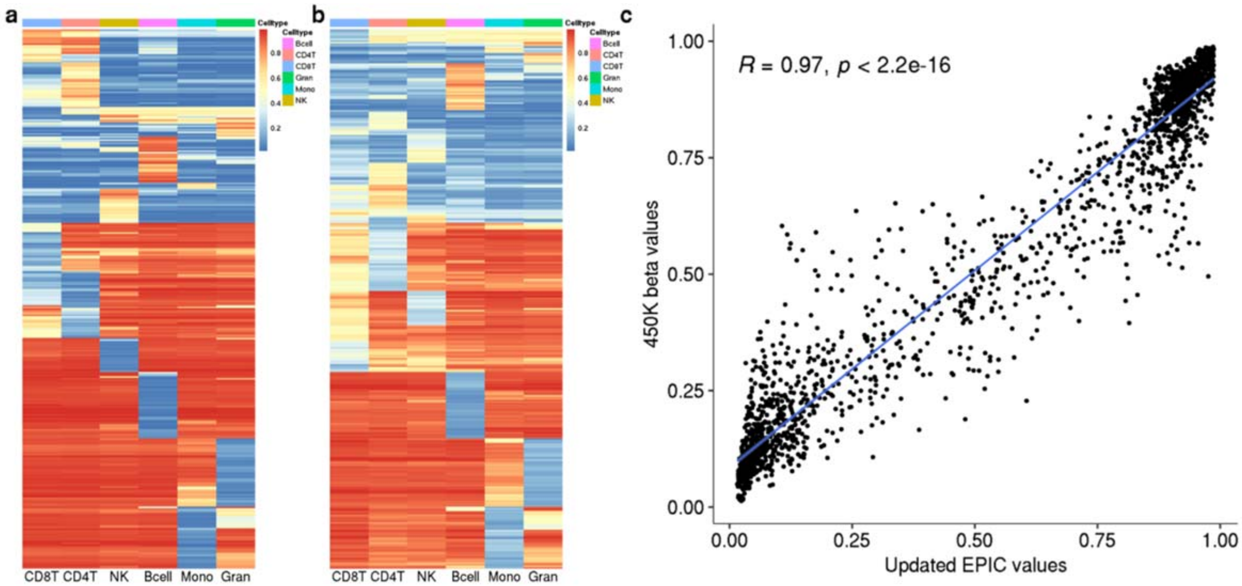

Fig 4. **a**, Heatmap of the updated basis matrix for the EPIC array using the method and data presented in the current manuscript. **b**, For comparison, the heatmap using the standard Houseman et al. basis (Houseman et al., 2012) for the 450K array is shown. The method utilized in the manuscript generates somewhat better cell type discrimination **c**, Scatter plot of the 450K values against the updated EPIC basis values showing high correlation.

Reference

1. Roy, Roshni, et al. "DNA methylation signatures reveal that distinct combinations of transcription factors specify human immune cell epigenetic identity." *Immunity* 54.11 (2021): 2465-2480
2. Houseman EA, Accomando WP, Koestler DC, Christensen BC, Marsit CJ, Nelson HH, Wiencke JK, Kelsey KT (2012) DNA methylation arrays as surrogate measures of cell mixture distribution. BMC Bioinformatics 13: 86

Revised responses to previous review from Referee #3

The revised version of the paper "A methylation clock model of mild SARS-CoV-2 infection provides insight into immune dysregulation" has improved. However, a number of major concerns still remain:

1) The authors did not address my concern about their approach to regress out cell-type fractions in the M-value basis. In fact, in Methods the authors still state "We further regressed out cell type proportions by linear regression in the M-value space and created the cell-type corrected beta value matrix for some downstream analysis".

Authors' Response: All differential methylation analysis was performed in B-value space. Modeling was performed on B-values following batch correction with ComBat. The only analysis performed in M value space was the matrix processing for clustering shown in main Fig. 2A. This temporal trajectory clustering analysis was performed using B2M2B (Table 1 below) because we wanted to eliminate cell type proportion as a confounder. This regression was done in M-space in order to more accurately conform to the assumptions of linear regression (B values are beta distributed and have more variance at intermediate values in comparison to the extremes). As we demonstrate below, our cell-type correction pipeline and the one suggested by the reviewer (B_regress in Table 2 below) produce similar results (Fig. 5 below).

**Name**	**Normalization Pipeline**
Raw	No regression
B2M2B (current)	Raw->converted to M value-> regress out the cell type proportion -> converted to beta values.
B_regress	Raw -> regress out the cell type proportion directly in the beta value space

Table 2. Three normalization pipelines.

“Raw” represents the methylation matrix in the beta value space without any regression. “B2M2B” represents the methylation matrix that was currently adopted for the trajectory clustering in Fig. 2A after the following conversion: Raw->converted to M value-> regress out the cell type proportion -> converted to beta values. “B_regress” represents the methylation matrix regressing out the cell type proportion in the beta value space (Raw -> regress out the cell type proportion directly in the beta value space).

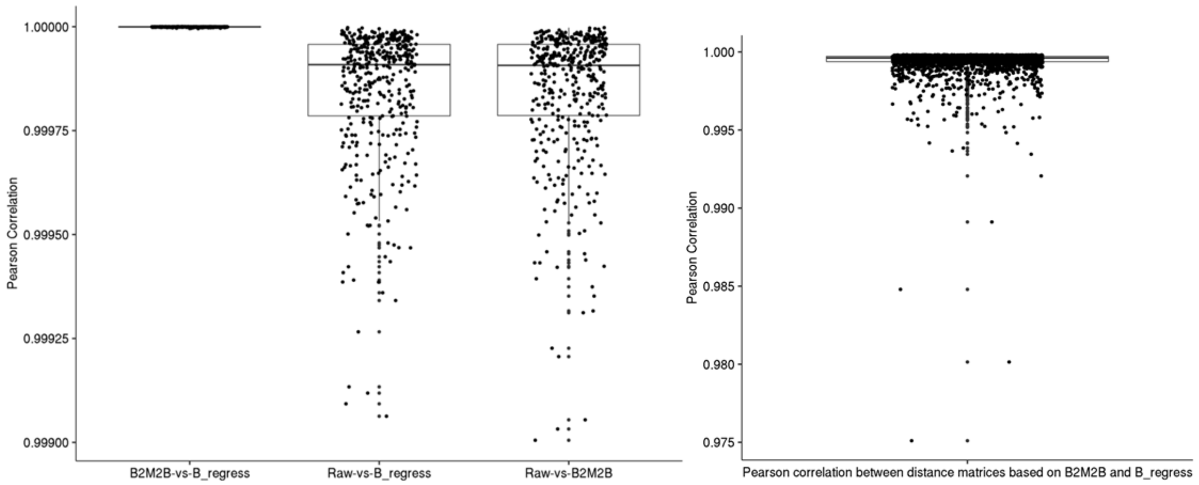

Figure 5. Left Panel: Pearson correlations between corresponding methylation samples across all 707,361 CpGs following different normalization pipelines. Right Panel: Comparison of distance matrices (dynamic warping distance), the measure used for the clustering in Fig. 2A, calculated using B2M2B and B_regress. This panel demonstrates that there is little difference using either pipeline for generating the data used in the clustering of the regulated sites shown in the manuscript.

The goal of the analysis shown in Fig. 2 in the manuscript is to achieve the best clustering possible for summarization of the patterns of response and for supporting biological interpretation. We have performed the B_regress analysis as suggested by the reviewer. Although the re-analysis does not change the conclusions of the manuscript, we find that the clustering obtained with our original B2M2B analysis is objectively better than that obtained with a B_regress analysis. We judge the quality of the analysis in two ways: 1) are the clusters better distinguished and 2) are the clusters more biologically coherent (i.e. does annotation of the clusters--for example TF binding sites--show better or worse discrimination among the clusters). For point #1, we determined total within-cluster variances (SSE), shown in the table below. Smaller values indicate a better fit. As can be seen from Table1 above, the B2M2B clustering assignment is better. For point #2, if we look at the TF annotation of each cluster in the original B2M2B analysis vs. the B_regress analysis, the B_regress analysis fails to identify any unique TFs significantly associated with Clusters 2, 6 and 7, whereas annotation of the original B2M2B analysis identifies unique TFs for all 7 clusters. Furthermore there were only 45 unique TFs with

the B_regress analysis and 61 with the B2M2B (Fig. 1b and 2b above). These findings all support the formulation that the B2M2B analysis is better because the clusters obtained are more distinct and show more distinct TF annotation results.

This may seem somewhat counterintuitive, but clustering solves an NP-hard combinatorial problem with a greedy heuristic algorithm. What our results suggest is that the application of this greedy algorithm to B2M2B provides a better solution to the global optimization problem.

We appreciate that the B_regress analysis is more common and that some readers would like to know what the results were with B_regress analysis. However, while it does not alter the conclusions of the manuscript, we believe the clustering based on B_regress analysis is inferior. Therefore, we propose to leave the B2M2B analysis in the main figure and include the B_regress analysis and annotation as a supplementary figure. The methods have been revised to explain the rationale for the B2M2B analysis and to cite the alternative results.

I tried to explain to the authors that statistically speaking this is problematic because the natural exact relationship that exists between beta-values and cell-type proportions (a beta-value in tissue can be expressed exactly as a linear sum of cell-type proportions and beta-values in the actual cell-types) is "destroyed" when you transform beta-values to M-values.

Authors' Response: This is a technical detail that could be reasonably argued in different ways. For example, one could also argue that doing least squares regression in beta space, as the reviewer insists, is not valid as the residuals are not independent of the mean. This study (https://bmcbioinformatics.biomedcentral.com/articles/10.1186/1471-2105-11-587) argues that M value analysis is theoretically preferred. Generally, logit transforming proportion values is a standard approach for dealing with heteroscedasticity and a proper treatment of beta regression is quite complex (https://cran.r-project.org/web/packages/betareg/vignettes/betareg.pdf). Thus there are many possible approaches and different theoretical arguments for the validity of both the beta space analysis preferred by the reviewer and M-space analysis we performed for the clustering figure in 2A. We prefer M-value for the temporal trajectory clustering due to a distribution for this specific analysis and because the clustering we obtain is objectively better. Moreover, as we show in the response to the previous comment and to Reviewer #4 comment 1 above, the clustering obtained with the B2M2B analysis is better.

Hence, the "regressing out" of cell-type proportions in the M-value space leads to a biased residual matrix. In any case, the author's statement in Methods plainly contradicts their responses provided in their rebuttal letter, because in the rebuttal they categorically stated that they only used M-values for batch correction. In fact, as written in Methods, one gets the impression that the authors have regressed out cell-type fractions in the M-value basis and then corrected for cell-type fractions again when performing DMS-type analyses. This

again is poor scholarship, because you don't need and you should never adjust for the same factors twice.

Authors' Response: We did not correct for cell type fractions twice. We have reviewed the methods and revised the wording to ensure that it is clear the M type regression was used only for the Fig. 2A analysis.The relevant sub-section of the Methods now reads:

We estimated the proportions of six major cell types (B cells, Granulocytes, Monocytes, NK cells, CD4 T cells and CD8 T cells) using standard reference-based method (Houseman et al, 2012). We took the original CellType450K basis matrix and replaced the values with those from (Roy et al, 2021) (Illumina Methylation microarray). This was done to help remove bias induced by the platform inconsistency. We compared cell type specificity obtained with the updated basis matrix to that obtained using the standard Houseman et al. basis (Houseman et al., 2012). We found that the cell-type specificity blocks were preserved and in some cases actually improved in the updated matrix. In particular, we find that the hypomethylated values are generally lower in the new basis (Appendix Figure S9a-b). The overall correlation of the standard basis values against the updated basis values is nearly perfect (Appendix Figure S9c).
The differential methylation site analysis was performed on raw beta values using these cell type proportions as covariates (see Materials and Methods, Sub-section 5). For clustering analysis, we created a cell type-corrected matrix by regressing out cell type proportions first (see our elaboration in Sub-section 7). The machine learning models used the raw beta value matrix (see Sub-section 10).

I understand that how exactly you perform these analysis may not greatly affect results on this particular dataset, yet this is not a valid reason for not doing things properly. On other datasets, the specifics of the method could greatly impact results. The important thing here is to retain a certain level of scholarship

Authors' Response: The reviewer asks us to ascertain that our analysis, which is acknowledged to "not greatly affect results on this particular dataset" must be appropriate for datasets we are not analyzing in this study. We are not presenting a tool for general use. If the reviewer believes, as implied, that the scientific results are valid using the analysis performed, there is no reasonable grounds to insist on a different analysis.

2) A similar comment applies to the author's refusal to regenerate a new EPIC DNAm reference matrix for blood from the Roy et al data. Whilst I agree that replacing the values in the older 450k DNAm reference matrix with EPIC-derived ones, should work, it again reflects poor scientific reasoning and scholarship. That the authors express so much concern about differences in 450k and EPIC arrays, raises the question why they don't derive a new DNAm reference matrix from Roy et al data, which is EPIC, thus circumventing their original concern. Indeed, given that not all 450k probes are on EPIC arrays, isn't the loss of probes a bigger concern than very small shifts in the mean DNAm values between 450k and EPIC? It seems to me that the authors are just cherry-picking their concerns, whilst ignoring others. Again, whilst I agree that all of this will not greatly

affect downstream analyses in this particular study, it is generally speaking poor scholarship to replace the entries of a 450k DNAm reference matrix with values from an EPIC array because those values also derive from *different sets of samples*, so you are not altering the values simply by a technical difference but also by a biological difference (e.g. different ages).

Authors' Response: We respectfully disagree with this point. Generating a basis matrix essentially amounts to probe selection (as the values are typically taken to be the mean). However, given just a full reference matrix, feature selection is not a well specified optimization problem. Instead, heuristic algorithms are used in this step (Koestler 2016 DOI: 10.1186/s12859-016-0943-7; Salas 2018 DOI: 10.1186/s13059-018-1448-7) and any new basis matrix has to be extensively validated on datasets with known ground truth. This would be entirely outside the scope of our study. Furthermore, this issue is not pertinent to our study, as acknowledged by the reviewer when stating " that all of this will not greatly affect downstream analyses in this particular study."

To provide additional evidence for the appropriateness of the analysis we perform, please see the response to comment #2 of Reviewer #4 above.

3) Finally, I was also not persuaded by the author's argument in relation to Fig.3a. For the task of predicting time since infection, I would strongly argue that it is the absolute error estimate that matters. A Pearson correlation is not applicable to ONE new sample for which you need to predict the time from infection. However, the difference between predicted time and actual time is applicable to ONE new sample. Hence, based on Fig.3a, I find it very troubling that the MSE is quite large, for instance, even for samples at 30/40 days, we can see that the error is a whopping 10 days. How is such a large error applicable in clinical practice? I honestly don't see how this advances the field.

Authors' Response: The reviewer raised a technical issue in the initial manuscript review. As the reviewer now raises only a new concern about clinical utility, we assume the response to the previous technical comment is in fact satisfactory to the reviewer. This concern about clinical value was not raised in the previous review, so it could not have been previously addressed.

In any event, we make no claims in the manuscript about the use of our time since infection model in clinical practice. We do not know whether predicting time since infection would have clinical practice value. We feel that the importance of this analysis is to demonstrate that this relatively short time scale prediction is statistically feasible. Given that we have few samples at longer time points, our study provides a lower bound on the duration of the "predictable" period. This is of interest as it informs potential mechanisms.

2nd Revision - Editorial Decision 20th Feb 2023

Thank you again for sending us your revised manuscript. We are now satisfied with the modifications made and I am pleased to inform you that your paper has been accepted for publication.

EMBO Press Author Checklist

Corresponding Author Name: Stuart C. Sealfon and Maria Chikina	
Journal Submitted to: Molecular Systems Biology	
Manuscript Number: MSB-2022-11361R	

USEFUL LINKS FOR COMPLETING THIS FORM
The EMBO Journal - Author Guidelines
EMBO Reports - Author Guidelines
Molecular Systems Biology - Author Guidelines
EMBO Molecular Medicine - Author Guidelines

Reporting Checklist for Life Science Articles (updated January
This checklist is adapted from Materials Design Analysis Reporting (MDAR) Checklist for Authors. MDAR establishes a minimum set of requirements in transparent reporting in the life sciences (see Statement of Task: 10.31222/osf.io/9sm4x). Please follow the journal's guidelines in preparing your
Please note that a copy of this checklist will be published alongside your article.

Abridged guidelines for figures
1. Data
The data shown in figures should satisfy the following conditions:
- the data were obtained and processed according to the field's best practice and are presented to reflect the results of the experiments in an accurate and unbiased manner.
- ideally, figure panels should include only measurements that are directly comparable to each other and obtained with the same assay.
- plots include clearly labeled error bars for independent experiments and sample sizes. Unless justified, error bars should not be shown for technical
- if n<5, the individual data points from each experiment should be plotted. Any statistical test employed should be justified.
- Source Data should be included to report the data underlying figures according to the guidelines set out in the authorship guidelines on Data

2. Captions
Each figure caption should contain the following information, for each panel where they are relevant:
- a specification of the experimental system investigated (eg cell line, species name).
- the assay(s) and method(s) used to carry out the reported observations and measurements.
- an explicit mention of the biological and chemical entity(ies) that are being measured.
- an explicit mention of the biological and chemical entity(ies) that are altered/varied/perturbed in a controlled manner.
- the exact sample size (n) for each experimental group/condition, given as a number, not a range;
- a description of the sample collection allowing the reader to understand whether the samples represent technical or biological replicates (including how many animals, litters, cultures, etc.).
- a statement of how many times the experiment shown was independently replicated in the laboratory.
- definitions of statistical methods and measures:
  - common tests, such as t-test (please specify whether paired vs. unpaired), simple χ2 tests, Wilcoxon and Mann-Whitney tests, can be unambiguously identified by name only, but more complex techniques should be described in the methods section;
  - are tests one-sided or two-sided?
  - are there adjustments for multiple comparisons?
  - exact statistical test results, e.g., P values = x but not P values < x;
  - definition of 'center values' as median or average;
  - definition of error bars as s.d. or s.e.m.

Please complete ALL of the questions below.
Select "Not Applicable" only when the requested information is not relevant for your study.

Materials

Newly Created Materials	Information included in the manuscript?	In which section is the information available? (Reagents and Tools Table, Materials and Methods, Figures, Data Availability Section)
New materials and reagents need to be available; do any restrictions apply?	Yes	Data Availability Section

Antibodies	Information included in the manuscript?	In which section is the information available? (Reagents and Tools Table, Materials and Methods, Figures, Data Availability Section)
For **antibodies** provide the following information:<br>- Commercial antibodies: RRID (if possible) or supplier name, catalogue number and or/clone number<br>- Non-commercial: RRID or citation	Not Applicable	

DNA and RNA sequences	Information included in the manuscript?	In which section is the information available? (Reagents and Tools Table, Materials and Methods, Figures, Data Availability Section)
**Short novel DNA or RNA including primers, probes**: provide the sequences.	Yes	Data Availability Section

Cell materials	Information included in the manuscript?	In which section is the information available? (Reagents and Tools Table, Materials and Methods, Figures, Data Availability Section)
**Cell lines:** Provide species information, strain. Provide accession number in repository **OR** supplier name, catalog number, clone number, and/**OR** RRID.	Not Applicable	
**Primary cultures:** Provide species, strain, sex of origin, genetic modification status.	Not Applicable	
Report if the cell lines were recently **authenticated** (e.g., by STR profiling) and tested for mycoplasma contamination.	Not Applicable	

Experimental animals	Information included in the manuscript?	In which section is the information available? (Reagents and Tools Table, Materials and Methods, Figures, Data Availability Section)
**Laboratory animals or Model organisms:** Provide species, strain, sex, age, genetic modification status. Provide accession number in repository **OR** supplier name, catalog number, clone number, **OR** RRID.	Not Applicable	
**Animal observed in or captured from the field:** Provide species, sex, and age where possible.	Not Applicable	
Please detail **housing and husbandry conditions**.	Not Applicable	

Plants and microbes	Information included in the manuscript?	In which section is the information available? (Reagents and Tools Table, Materials and Methods, Figures, Data Availability Section)
**Plants:** provide species and strain, ecotype and cultivar where relevant, unique accession number if available, and source (including location for collected wild specimens).	Not Applicable	
**Microbes:** provide species and strain, unique accession number if available, and source.	Not Applicable	

Human research participants	Information included in the manuscript?	In which section is the information available? (Reagents and Tools Table, Materials and Methods, Figures, Data Availability Section)
If collected and within the bounds of privacy constraints report on age, sex and gender or ethnicity for all study participants.	Yes	Figure EV1

Core facilities	Information included in the manuscript?	In which section is the information available? (Reagents and Tools Table, Materials and Methods, Figures, Data Availability Section)
If your work benefited from core facilities, was their service mentioned in the acknowledgments section?	Yes	Acknowledgements

Design

Study protocol	Information included in the manuscript?	In which section is the information available? (Reagents and Tools Table, Materials and Methods, Figures, Data Availability Section)
If study protocol has been **pre-registered, provide DOI in the manuscript**. For clinical trials, provide the trial registration number **OR** cite DOI.	Not Applicable	
Report the **clinical trial registration number** (at ClinicalTrials.gov or equivalent), where applicable.	Not Applicable	

Laboratory protocol	Information included in the manuscript?	In which section is the information available? (Reagents and Tools Table, Materials and Methods, Figures, Data Availability Section)
Provide DOI OR other citation details if **external detailed step-by-step protocols** are available.	Not Applicable	

Experimental study design and statistics	Information included in the manuscript?	In which section is the information available? (Reagents and Tools Table, Materials and Methods, Figures, Data Availability Section)
Include a statement about **sample size** estimate even if no statistical methods were used.	Yes	Figure EV1
Were any steps taken to minimize the effects of subjective bias when allocating animals/samples to treatment (e.g. **randomization procedure**)? If yes, have they been described?	Not Applicable	
Include a statement about **blinding** even if no blinding was done.	Yes	Materials and Methods
Describe **inclusion/exclusion criteria** if samples or animals were excluded from the analysis. Were the criteria pre-established?<br><br>If sample or data points were omitted from analysis, report if this was due to attrition or intentional exclusion and provide justification.	Yes	Materials and Methods (Methylation subsection: exclusion of outliers)
For every figure, are **statistical tests** justified as appropriate? Do the data meet the assumptions of the tests (e.g., normal distribution)? Describe any methods used to assess it. Is there an estimate of variation within each group of data? Is the variance similar between the groups that are being statistically compared?	Yes	Materials and Methods; Figure legends

Sample definition and in-laboratory replication	Information included in the manuscript?	In which section is the information available? (Reagents and Tools Table, Materials and Methods, Figures, Data Availability Section)
In the figure legends: state number of times the experiment was **replicated** in laboratory.	Not Applicable	
In the figure legends: define whether data describe **technical or biological replicates**.	Not Applicable	

Ethics

Ethics	Information included in the manuscript?	In which section is the information available? (Reagents and Tools Table, Materials and Methods, Figures, Data Availability Section)
Studies involving **human participants**: State details of **authority granting ethics approval** (IRB or equivalent committee(s), provide reference number for approval.	Yes	Materials and Methods
Studies involving **human participants**: Include a statement confirming that **informed consent** was obtained from all subjects and that the experiments conformed to the principles set out in the WMA Declaration of Helsinki and the Department of Health and Human Services Belmont Report.	Yes	Materials and Methods
Studies involving **human participants:** For publication of **patient photos**, include a statement confirming that consent to publish was obtained.	Not Applicable	
Studies involving experimental **animals**: State details of **authority granting ethics approva**l (IRB or equivalent committee(s), provide reference number for approval. Include a statement of compliance with ethical regulations.	Not Applicable	
Studies involving **specimen and field samples:** State if relevant **permits** obtained, provide details of authority approving study; if none were required, explain why.	Yes	Materials and Methods

Dual Use Research of Concern (DURC)	Information included in the manuscript?	In which section is the information available? (Reagents and Tools Table, Materials and Methods, Figures, Data Availability Section)
Could your study fall under dual use research restrictions? Please check biosecurity documents and list of **select agents and toxins** (CDC): https://www.selectagents.gov/sat/list.htm	Not Applicable	
If you used a select agent, is the security level of the lab appropriate and reported in the manuscript?	Not Applicable	
If a study is subject to dual use research of concern regulations, is the name of the **authority granting approval and reference number** for the regulatory approval provided in the manuscript?	Not Applicable	

Reporting

The MDAR framework recommends adoption of discipline-specific guidelines, established and endorsed through community initiatives. Journals have their own policy about requiring specific guidelines and recommendations to complement MDAR.

Adherence to community standards	Information included in the manuscript?	In which section is the information available? (Reagents and Tools Table, Materials and Methods, Figures, Data Availability Section)
State if relevant guidelines or checklists (e.g., **ICMJE, MIBBI, ARRIVE, PRISMA**) have been followed or provided.	Not Applicable	
For **tumor marker prognostic studies,** we recommend that you follow the **REMARK** reporting guidelines (see link list at top right). See author guidelines, under 'Reporting Guidelines'. Please confirm you have followed these guidelines.	Not Applicable	
For **phase II and III randomized controlled trials**, please refer to the **CONSORT** flow diagram (see link list at top right) and submit the CONSORT checklist (see link list at top right) with your submission. See author guidelines, under 'Reporting Guidelines'. Please confirm you have submitted this list.	Not Applicable	

Data Availability

Data availability	Information included in the manuscript?	In which section is the information available? (Reagents and Tools Table, Materials and Methods, Figures, Data Availability Section)
Have **primary datasets** been deposited according to the journal's guidelines (see 'Data Deposition' section) and the respective accession numbers provided in the Data Availability Section?	Yes	Data Availability Section
Were **human clinical and genomic datasets** deposited in a public access-controlled repository in accordance to ethical obligations to the patients and to the applicable consent agreement?	Yes	Data Availability Section
Are **computational models** that are central and integral to a study available without restrictions in a machine-readable form? Were the relevant accession numbers or links provided?	Yes	N/A
If publicly available data were reused, provide the respective **data citations in the reference list**.	Yes	Results; Figure 5e; Appendix Figure S4; Table EV8
